# Multi-dimensional impact assessment for priority setting of agricultural technologies: An application of TOPSIS for the drylands of sub-Saharan Africa and South Asia

**DOI:** 10.1371/journal.pone.0314007

**Published:** 2024-11-21

**Authors:** Sika Gbegbelegbe, Arega Alene, Nedumaran Swamikannu, Aymen Frija

**Affiliations:** 1 International Institute of Tropical Agriculture (IITA)–Malawi, Lilongwe, Malawi; 2 International Crops Research Institute for the Semi-Arid Tropics (ICRISAT), Patancheru, Hyderabad, Telangana, India; 3 International Center for Agricultural Research in the Dry Areas (ICARDA), Tunis, Tunisia; Nepal Agricultural Research Council, NEPAL

## Abstract

The importance for multi-dimensional priority-setting of agricultural innovations is growing, given that agricultural technologies usually play multiple roles for smallholder farmers. This study assesses agricultural technologies based on their multi-dimensional impacts in the drylands of sub-Saharan Africa and South Asia. The study applies the Technique for Order Preference by Similarity to an Ideal Solution (TOPSIS) to a set of promising agricultural technologies and uses three outcome criteria: the benefit-cost ratio, poverty reduction, and nutrition security. The technologies are related to important cereals and grain legumes grown in these regions: sorghum, pearl millet, and finger millet; groundnut, cowpea, chickpea, lentil, pigeon pea, and soybean. The results show that the top technologies based on individual criteria can differ from the top technologies identified using a combination of criteria. For example, in semi-arid southern Africa, a promising technology which involves integrated pest management for cowpea ranks among the top five technologies which can reduce poverty. However, the analysis involving TOPSIS shows that nutrition security is more important in that region compared to poverty. As such, the top 5 technologies with the highest multi-dimensional impact for semi-arid southern Africa do not involve a cowpea technology; rather, they are all related to pigeon pea, a nutritious grain legume which is currently more consumed in that region compared to cowpea. One limitation of this study is that it did not consider all the roles of agricultural technologies in the drylands of sub-Saharan Africa and South Asia; this should be considered in future studies involving TOPSIS or other MCDM techniques. Nevertheless, the study shows that TOPSIS can successfully be used for multi-dimensional ex-ante impact assessment of agricultural technologies, and thus can support the prioritization of investments targeting agricultural research for development.

## 1 Introduction

Priority setting for agricultural innovations involves a selection process over multiple research activities on innovations. Over the years, the Consultative Group on International Agricultural Research (CGIAR) in collaboration with national research and development partners has conducted priority-setting exercises to guide the allocation of limited resources across agricultural innovations aimed at achieving positive impacts on food and nutrition security, poverty, and natural resource management [1]. The outcome of prioritization exercises has usually been a ranking of research programs, agricultural technologies, or agricultural policy options which can enhance various societal outcomes [2–4].

Past priority setting efforts have used one or two criteria like economic benefits (cost-benefit) [3], poverty reduction potential [3], the value of production, and even import dependence [2, 4]. However, the CGIAR Strategy and Results Framework for 2016–2030 (SRF 2016–2030) aims to positively influence multiple outcomes at once; these outcomes include poverty, food security, nutritional security, and natural resource systems & ecosystems services. Hence, new methodologies are needed that can consider multiple outcomes/criteria at once for priority-setting of improved agricultural technologies.

This study uses the Multi-Criteria Decision Making (MCDM) approach for multi-dimensional ex-ante impact analysis of agricultural technologies. The MCDM approach is used to rank options based on multiple criteria at once. The approach was first applied to the field of ‘energy and environment’, where it was often used to support the optimum selection of energy systems [5–7]. Then, its use was extended to other fields including supply chain management [7–9], operations research [7], software security [7, 10, 11], software performance and reliability [7, 12, 13], tourism management [7] and more recently adaptation to climate extremes [14, 15].

The MCDM approach involves two methods: discrete Multi-Attribute Decision-Making (MADM) and continuous Multi-Objectives Decision-Making (MODM) [7]. The discrete MADM method implies a finite number of alternatives/options/technologies, and the aim is to identify the alternative which will provide the greatest benefits [16]. The continuous MODM method involves an infinite number of alternatives, and the aim here is to design an alternative within well-defined constraints [16]. The discrete MADM has been more commonly used in agriculture compared to the continuous MODM [17, 18]. Some of the specific techniques used in MCDM include the Analytic Hierarchy Process (AHP) and the Technique for Order of Preference by Similarity to Ideal Solution (TOPSIS) [7]. In the agricultural field, the AHP has been applied more often to mapping land suitability for agricultural activities [19–24] or mapping vulnerabilities to specific threats [25–28]. On the other hand, TOPSIS has been more often used to identify optimal green energy sources [29–34]; it has also been used for ex post assessment of transformational changes at various spatial scales [35–37].

To the best of our knowledge, this study is the first that uses MCDM to quantity the multi-dimensional impact of technologies on multiple societal outcomes such as poverty and nutrition security. The study uses the discrete MADM method as it applies TOPSIS to selected criteria and derives a multi-dimensional ranking system for a finite number of promising agricultural technologies targeting the drylands of sub-Saharan Africa and South Asia. The technologies are related to the main cereals and grain legumes grown in these drylands: sorghum, pearl millet, and finger millet; groundnut, cowpea, chickpea, lentil, pigeon pea, and soybean. The rest of the paper is organized as follows. Section 2 provides a description of the methodology used in this study, whereas Section 3 describes the results for the study. Section 4 provides a discussion on the results and the final section concludes with a key summary of the study findings and implications for future studies.

## 2 Methods and data

### 2.1 Methodological framework for specific criteria

**Fig 1:**
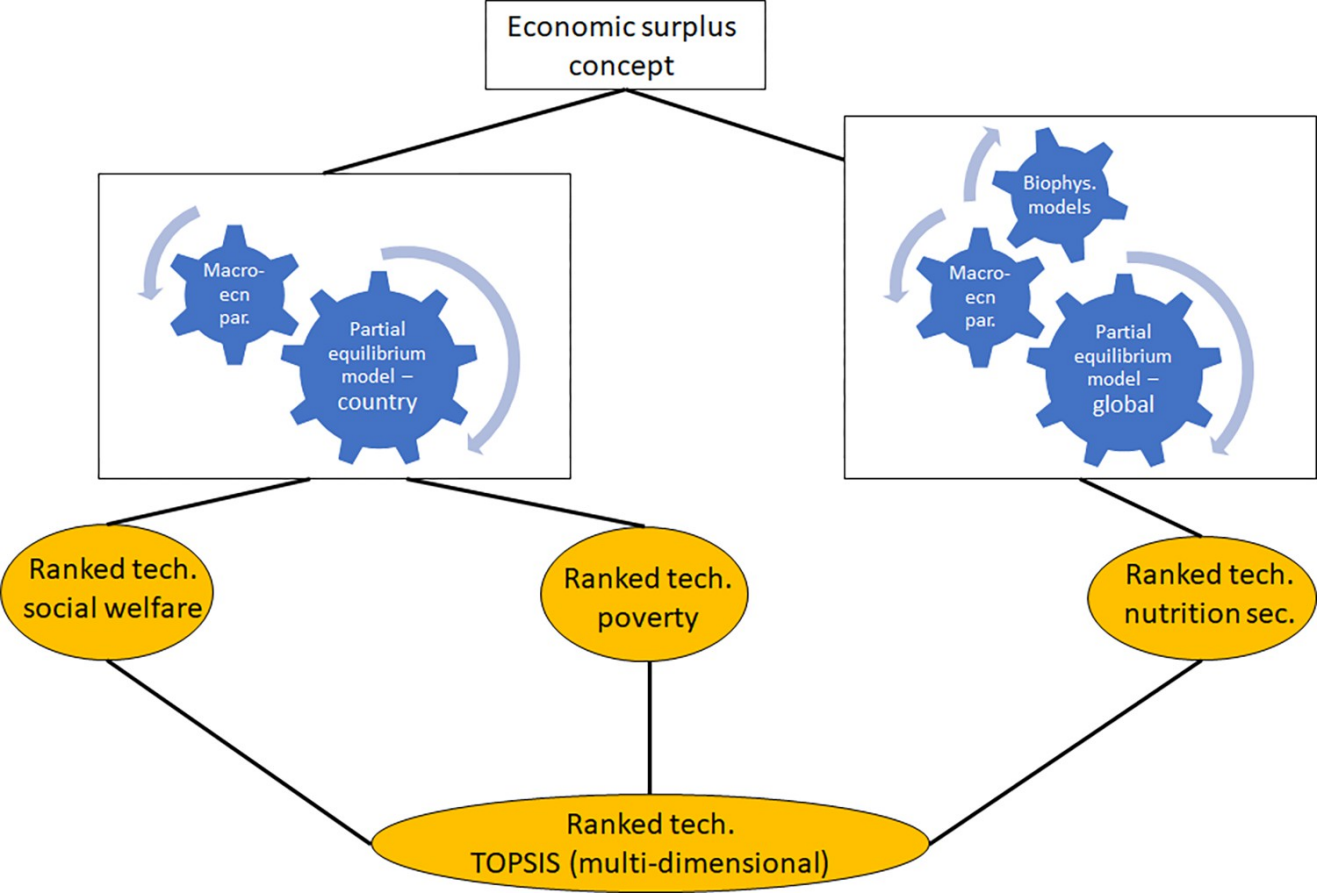
Methodological framework. Source: authors.

For this study, two dimensions of the role played by agricultural technologies are considered: welfare and nutrition security. Two criteria are used for welfare: Benefit-Cost Ratio (BCR) and poverty reduction. One criterion for nutrition security is used: number of malnourished children. All technologies are first ranked based on their potential impact on each criterion, namely the BCR, poverty and nutrition security. Afterwards, TOPSIS is used to provide a ranking which combines the three criteria (Fig 1). In studies where criteria importance is subjective, TOPSIS is usually combined with the AHP to enhance weight accuracy [30, 38, 39]. However, in this study, the criteria weights are defined using a purely quantitative approach; as such, the AHP is not required for criteria weight determination.

The economic surplus concept is used to quantify the impact of agricultural technologies on the benefit-cost ratio, poverty, and nutrition security (Fig 1). The economic surplus concept is a proven concept for quantifying the potential economic benefits/losses that agricultural technologies can bring to producers, consumers, and governments [40]. These benefits/losses can be measured through general or partial equilibrium models. Unlike general equilibrium models, partial equilibrium models do not account for the feedback effect of improved technologies on incomes for farmers and other actors along agricultural value chains. Hence, partial equilibrium models, which are used in this study, provide a gross estimate of the change in social welfare brought by improved agricultural technologies [40]. The changes that one agricultural technology brings to the production of a target crop can be used to estimate changes in poverty and nutrition security.

For this study, a country-level partial equilibrium model is used to quantify the impact of the technologies on the benefit-cost ratio and poverty, whereas a global partial equilibrium model, the International Model for the Policy Analysis of Agricultural Commodities and Trade (IMPACT) is used to quantify the impact of the technologies on nutrition security (Fig 1). In the global partial equilibrium model, elasticity values for demand and production change slightly over time to reflect changes in tastes and preferences for consumers and changes in production flexibility for producers [41]. In addition, the future demand for the target crops changes due to rising population and changing incomes. Also, the future production of the crops changes due to exogenous productivity changes reflecting technological change affecting all crops in a country, and endogenous change in water availability. All such changes in elasticity values, rising population, changing incomes and exogenous changes in production are assumed to be non-existent in the country-level partial equilibrium model.

In the country-level partial equilibrium model, the benefit-cost ratio (BCR) in social welfare is the ratio of social welfare to research and dissemination costs; its value is always positive; when it’s above one, economic benefits outweigh costs. the BCR is measured based on Alene et al. [3] and Alene et al. [42]:

$$BCR=\frac{\sum_{t=1}^{T}\sum_{n=1}^{N}\frac{{\Delta ES}_{t,n}}{{\left(1+r\right)}^{t}}}{\sum_{t=1}^{T}\frac{C_{t}}{{\left(1+r\right)}^{t}}}$$

Where:

BCR = benefit-cost ratio

Δ*ES*_*t*,*c*_ = change in economic surplus (producers, consumers, exporters importers and government) brought by the agricultural technology in country ‘*c*’ at time ‘*t*’

*r* = standard interest rate, 10%

*C*_*t*_ = research dissemination and extension costs at time ‘*t*’ for target environment

*n* = country in target environment

*t* = year t which goes from 1 to 25 years reflecting time between development of technology and year when the maximum adoption level is achieved

Here, the change in economic surplus is computed using the approach described by Alene et al. (2018), where it is assumed that regional produce prices are influenced by regional production and are also subject to seasonal fluctuations. If countries were assumed to be price-takers, a different approach would have been used. The poverty effects from an agricultural technology are also measured based on Alene et al. [3], as follows:

$$\Delta P_{t=T}=\left(\frac{\Delta {ES}_{t=T}}{AgGDP}*100\%\right)\left(\frac{\Delta P/P}{\Delta Y/Y}\right)(P)$$

Where:

Δ*P*_*t* = *T*_ = change in the number of poor people at year when maximum adoption of a technology has been achieved

Δ*ES*_*t* = *T*_ = total change in economic surplus at year when maximum adoption of a technology has been achieved

*Ag GDP* = agricultural value added at time *t* = 1

$\frac{\Delta P/P}{\Delta Y/Y}$ = poverty elasticity reflecting the percentage change in poverty when agricultural productivity increases by 1%

*P* = number of poor people at time *t* = 1

In the global partial equilibrium (IMPACT), nutrition security in a country is measured by the number of malnourished children which is estimated through various indicators that include per capita kilocalorie availability, relative life expectancy for women compared to men at a national scale, the proportion of females enrolled in secondary school, and the proportion of the population which has access to safe water [41]. Here, agricultural technologies influence the number of children malnourished by changing per capita kilocalorie availability under a scenario where future climate is assumed to be the same as that of the 2000s. More specifically, the number of malnourished children is measured based on [41] as follows:

$${MC}_{t}={PopC}_{t}\left(\frac{{PMC}_{t}}{100}\right)$$

Where:

$${PMC}_{t}={PMC}_{t-1}+\frac{\Delta MC/MC}{\Delta Kcal/Kcal}(log\frac{{PcKcal}_{t}}{{PcKcal}_{t-1}})+LfExpC({LfExp}_{t}-{LfExp}_{t-1})+SchlC({Schl}_{t}-{Schl}_{t-1})+WaterC({Water}_{t}-{Water}_{t-1})$$

*MC*_*t*_ = Total number of malnourished children in year ‘t’

*PopC*_*t*_ = number of malnourished children under 5 years of age in year ‘t’–national

*PMC*_*t*_ = percentage of malnourished children under 5 years of age in year ‘t’–national

*PMC*_*t*−1_ = percentage of malnourished children under 5 years of age in year ‘t-1’–national

$\frac{\Delta MC/MC}{\Delta Kcal/Kcal}$ = elasticity of children’s malnourishment relative to kilocalorie availability; value is ‘-25.24’

*PcKcal*_*t*_ = per capita kilocalorie availability based on national food supply in year ‘t’

*PcKcal*_*t*−1_ = per capita kilocalorie availability based on national food supply in year ‘t-1’

*LfExpC* = coefficient on children’s malnourishment relative life expectancy for women; value is ‘-71.755094’

*LfExp*_*t*_ = ratio of female over male for life expectancy at birth in year ‘t’

*LfExp*_*t*−1_ = ratio of female over male for life expectancy at birth in year ‘t-1’

*SchlC* = coefficient on children’s malnourishment relative to total female enrollment in secondary school; value is ‘-0.219831’

*Schl*_*t*_ = percent of total female enrollment in secondary school in year ‘t’–national

*Schl*_*t*−1_ = percent of total female enrollment in secondary school in year ‘t-1’–national

*WaterC* = coefficient on children’s malnourishment relative to the share of the population with access to safe water

*Water*_*t*_ = share of the population with access to safe water in year ‘t’–national

*Water*_*t*−1_ = share of the population with access to safe water in year ‘t-1’–national

### 2.2 Multi-criteria ranking using the TOPSIS

TOPSIS was applied to all three criteria to identify the technology which is closest to a positive ideal solution that encompasses all three criteria [43]. First, criteria weights were assigned to each criterion, based on the importance of each criterion in a target region. Then, TOPSIS was applied using six computational steps. In the first step, the results obtained for each criterion were normalized to facilitate comparisons across criteria. The second step applied the pre-identified weights to the matrix obtained in step 2. The third step involved computing the positive-ideal solution which reflects the best possible solution related to a technology set; it also involves computing the negative-deal solution which reflects the worst possible solution related to the technology set. The fourth step involved estimating the Euclidean distances between each proposed technology and the positive- and negative-ideal solutions. These distances were used in the fifth step to compute the closeness index which reflects the relative distance of each technology to the positive-ideal solution. The last step consists of identifying the best technologies using the closeness index: the higher the index, the better the technology [43]. For this study, all computations were done in Microsoft Excel.

**2.2.1 Determination of criteria weights.** The share of agriculture in Gross Domestic Product (GDP) in 2014/16 was used to assess the importance of the BCR for a target environment. For the poverty and nutrition security criteria, poverty headcount and child stunting headcount around 2015 were used to assess their importance (Table 1). The agricultural GDP data was retrieved from the World Bank [44]; poverty data was obtained from Wood et al. [45] and WorldPop [46]; and child stunting data was retrieved from the Center for International Earth Science Information Network [47].

**Table 1:** Estimated criteria weights.

	**Values in 2015 (%)**		
	Share of AgGDP in GDP (2014/16)	Poverty headcount (2015)	Child stunting (2015)
Europe & Central Asia	2	1	9
East Asia & Pacific	5	8	21
Latin America & Caribbean	5	5	18
South Asia	15	25	38
Sub-Saharan Africa	16	47	33
World	4	15	-
Dry sub-humid Eastern Africa	25	33	44
Semi-arid Eastern Africa	27	27	43
Dry sub-humid Western Africa	23	37	35
Semi-arid Western Africa	22	46	39
Dry sub-humid Southern Africa	6	32	42
Semi-arid Southern Africa	3	21	30
Dry sub-humid Southern Asia	15	36	59
Semi-arid Southern Asia	14	32	54
	**Ratios to compute weights**		
	Share of AgGDP in GDP	Poverty headcount	Child stunting
Dry sub-humid Eastern Africa	1.52	0.69	1.34
Semi-arid Eastern Africa	1.67	0.58	1.32
Dry sub-humid Western Africa	1.40	0.78	1.07
Semi-arid Western Africa	1.38	0.98	1.18
Dry sub-humid Southern Africa	0.36	0.68	1.28
Semi-arid Southern Africa	0.20	0.45	0.92
Dry sub-humid Southern Asia	0.97	1.46	1.54
Semi-arid Southern Asia	0.94	1.30	1.40
	**Normalized weights**		
	Share of AgGDP in GDP / Benefit-cost ratio	Poverty headcount / Poverty	Child stunting / Child malnutrition
Dry sub-humid Eastern Africa	0.43	0.20	0.38
Semi-arid Eastern Africa	0.47	0.16	0.37
Dry sub-humid Western Africa	0.43	0.24	0.33
Semi-arid Western Africa	0.39	0.28	0.33
Dry sub-humid Southern Africa	0.15	0.29	0.55
Semi-arid Southern Africa	0.13	0.29	0.58
Dry sub-humid Southern Asia	0.24	0.37	0.39
Semi-arid Southern Asia	0.26	0.36	0.38

Source: authors computations using data from the World Bank (https://data.worldbank.org/indicator) for agricultural GDP (AgGDP); data from Wood et al. [45] and WorldPop (https://www.worldpop.org/) for poverty; and data from the Center for International Earth Science Information Network [47] for child stunting

Based on estimates on the contribution of agriculture to Gross Domestic Product (GDP) in 2014/16, South Asia and sub-Saharan Africa had the highest shares compared to other parts of the world (Table 1). In addition, agriculture’s contribution to GDP in the drylands of western and eastern Africa (i.e. about 25%) was much higher than that of sub-Saharan Africa. This result suggests that the importance of agriculture, as a source of livelihood, is more pronounced in the drylands of western and eastern Africa compared to sub-Saharan Africa as a region. By contrast, the share of agriculture in GDP is smaller in the drylands of southern Africa compared to sub-Saharan Africa; such result reflects that other sectors namely, the industrial (especially mining) and/or services sectors, might be a more important source of livelihood in these regions. The drylands of South Asia have similar shares when compared to the region of South Asia (Table 1).

For poverty, the headcount in the drylands of South Asia is much higher than that of South Asia. More specifically, the poverty headcount in semi-arid and dry sub-humid South Asia is 32% and 36%, respectively. This contrasts with 25% for south Asia. The drylands in sub-Saharan Africa tend to register lower poverty headcounts compared to the average for the region. For example, the poverty headcount in dry sub-humid eastern Africa is 33% which is much lower than 47%, the poverty headcount for sub-Saharan Africa (Table 1).

For child stunting, the 2015 headcount was about 33% and 38% in sub-Saharan Africa and South Asia, respectively. Here, the drylands of South Asia have much higher stunting rates compared to the average for South Asia. The same also applies for the drylands of sub-Saharan Africa: most dryland regions have higher child stunting rates compared to the average for the region (Table 1).

The estimates for child stunting headcount, poverty headcount and the share of agriculture in GDP are divided by the estimates for sub-Saharan Africa to derive criteria weights. These ratios are then normalized to sum up to one. For example, for dry sub-humid eastern Africa, the share of agriculture in GDP is about 1.52 times higher than for sub-Saharan Africa; similarly, the poverty headcount is about 0.7 times that for the region whereas the child stunting headcount is 1.34 times higher than that of the region (Table 1). These three ratios are summed up and normalized to derive normalized weights. Hence, given that the share of agriculture in GDP has the highest ratio (1.52), it also has the highest weight of 43%. The child stunting indicator has the second-highest ratio (1.34) and the second-highest weight of about 38%. The poverty indicator has a weight of 20%. These three weights are consistent with the fact that agriculture’s contribution to GDP in dry sub-humid eastern Africa is higher than that for sub-Saharan Africa; the poverty headcount is lower and child stunting is more severe. A similar approach is used to derive weights for all other dryland regions in sub-Saharan Africa. For the drylands of South Asia, comparisons are made relative to the region of South Asia (Table 1).

#### 2.2.2 The steps for TOPSIS

The first step in applying TOPSIS consists of generating the normalized decision matrix which contains an index value for each criterion for each technology. Each element *r*_*ij*_ in this first matrix containing the normalized indices is computed based on [43] as follows:

$$r_{ij}=\frac{a_{ij}}{\sqrt{\sum_{i=1}^{I}a_{ij}^{2}}}$$

Where:

*r*_*ij*_ = the normalized value for criterion ‘j’ and technology ‘i’

*a*_*ij*_ = the value for criterion ‘j’ and technology ‘i’

The second step consists of generating the weighted normalized matrix as described in [43]. In this step, the predefined criteria weights are applied to the *r*_*ij*_ matrix to generate a second matrix, i.e. the weighted normalized decision matrix, *v*_*ij*_:

$$v_{ij}=[\begin{array}{c} v_{11}\;v_{12}\;v_{13}\\ v_{21}\;v_{22}\;v_{23}\\ \vdots \;\vdots \;\vdots \\ v_{i1}\;v_{i2}\;v_{i3} \end{array}]=[\begin{array}{c} w_{1}r_{11}\;w_{2}r_{12}\;w_{3}r_{13}\\ w_{1}r_{21}\;w_{2}r_{22}\;w_{3}r_{23}\\ \vdots \;\vdots \;\vdots \\ w_{1}r_{i1}\;w_{2}r_{i2}\;w_{J}r_{IJ} \end{array}]$$

Where:

*w*_*J*_ = the estimated weight of criterion j

In the third step, the positive- and negative-ideal solutions are derived using the *v*_*ij*_ matrix. The positive-ideal solution includes the best outcome for each criterion. The negative-ideal solution incorporates the worst outcomes for each criterion. The next step in the TOPSIS method consists of estimating the Euclidean distances between each technology and the positive- and negative-ideal solutions. The Euclidean distance to the positive-ideal solution, Sij+, is estimated as follows based on [43]:

$$S_{i}^{+}=\sqrt{\sum_{j=1}^{3}{\left(v_{ij}-v_{ij}^{+}\right)}^{2}}$$

Where:

i = 1,2,3, … I

$v_{ij}^{+}$ = matrix for positive ideal solution

Similarly, the Euclidean distance, $S_{ij}^{-}$, between each technology and the negative-ideal solution is estimated as follows based on [43]:

$$S_{i}^{-}=\sqrt{\sum_{j=1}^{3}{\left(v_{ij}-v_{ij}^{-}\right)}^{2}}$$

Where:

i = 1,2,3, … I

$v_{ij}^{-}$ = matrix for the negative ideal solution

The fifth step in the TOPSIS method consists of calculating the relative distance of each technology to the positive-ideal solution; such distance is called the closeness index ($C_{i}^{+}$) and is computed as follows based on [43]:

$$C_{i}^{+}=\frac{S_{i}^{-}}{S_{i}^{-}+S_{i}^{+}}$$

Where:

i = 1,2,3, … I

The closeness index is between 0 and 1. The higher the closeness index, the closer a technology is to the positive-ideal solution. The closeness index is then used to do a final ranking of all technologies in each mega-environment while simultaneously considering all criteria.

### 2.3 Description of technologies and associated data used for impact assessment

The methodological framework described in sections 2.1 and 2.2 was applied to improved technologies for the Grain Legumes and Dryland Cereals (GLDC) program; GLDC was a Challenge Research program (CRP) in the CGIAR [48]. In the GLDC CRP, target regions consist of the drylands of sub-Saharan Africa and South Asia (S1 to S8 Tables). These regions face soil constraints which hinder agricultural production; they are also prone to drought and are projected to experience an increase of more than 2 degree Celsius between 2005 and 2050 [49]. As such, improved technologies were identified which could tackle the current and emerging biotic and abiotic stresses affecting the growth of key staple and cash crops grown by communities in the drylands of SSA and South Asia [48]. The improved technologies were related to three dryland cereals, namely sorghum, pearl millet, and finger millet; they were also related to six grain legumes, namely groundnut, cowpea, chickpea, lentil, pigeon pea, and soybean (Tables A.1 to A.8) [48]. These crops have multi-purposes roles in dryland communities; they contribute to food and nutrition security; they also increase incomes through cash sales and provide animal fodder; the grain legumes in the drylands also play an important role in improving soil fertility [49]. Both the dryland cereals and grain legumes contribute positively toward carbon sequestration [50]. For this study, we are focusing on the roles of these crops on social welfare and nutrition security.

Agro-ecological characteristics were first used to derive target environments within the drylands of sub-Saharan Africa and South Asia [48]. These target environments include semi-arid and dry sub-humid West and Central Africa; semi-arid and dry sub-humid eastern Africa, semi-arid and dry sub-humid southern Africa; semi-arid and dry sub-humid South Asia [48].

For all improved technologies, technology adoption parameters were derived through consultation with 18 experts who are involved in developing the improved technologies and who are also knowledgeable about national processes on variety release and adoption pathways among farmers [3, 48]. The experts were grouped based on their expertise for the target crop. Each group worked together to generate and validate the data provided for each technology. The specific data provided by the experts were about the attributes and performance of the technologies in each target environment, and plausible adoption pathways for each proposed technology (S1 Table). For example, improved drought-tolerant cowpea with integrated crop management is targeted for semi-arid West and Central Africa (S1 Table). For this technology, the data gathered from the experts imply that the projected maximum adoption rate is 40% of cowpea acreage among farmers to be achieved within 10 years after release. The technology would increase cowpea yield by 70% with an 80% chance of success; it would reduce farm production cost per hectare by 10%. The research costs involved in developing the technology are about US$300,000 per year for 10 years; in addition, dissemination costs amount to US$50 per hectare (S1 Table).

Macro-level parameters were retrieved from FAOSTAT [51] and the World Bank [44] (S1 Table). More specifically, 2012 to 2014 data on prices, quantity and harvested area were retrieved from FAOSTAT. The agricultural GDP was derived by applying dryland shares in each target country to 2016 data on agricultural value-added retrieved from the World Bank (S1 Table). The share of drylands in each target country was retrieved from the European Space Agency [52] (S1 Table). Poverty rates from Wood et al. (2010) were applied to 2016 population data from WorldPop [53] to obtain the total population living below US$1.25 a day (S1 Table). All the IMPACT data used for quantifying the impact of technologies on child malnutrition is available in GitHub [54].

## 3 Results

### 3.1 Unidimensional ex ante impact assessment: BCR, poverty, and child malnutrition

In this section, we describe the results obtained from using one criterion at a time for the ex ante impact assessment of promising technologies targeting the drylands in Sub-Saharan Africa and South Asia.

#### 3.1.1 Unidimensional ex ante impact assessment: West and Central Africa

**Fig 2:**
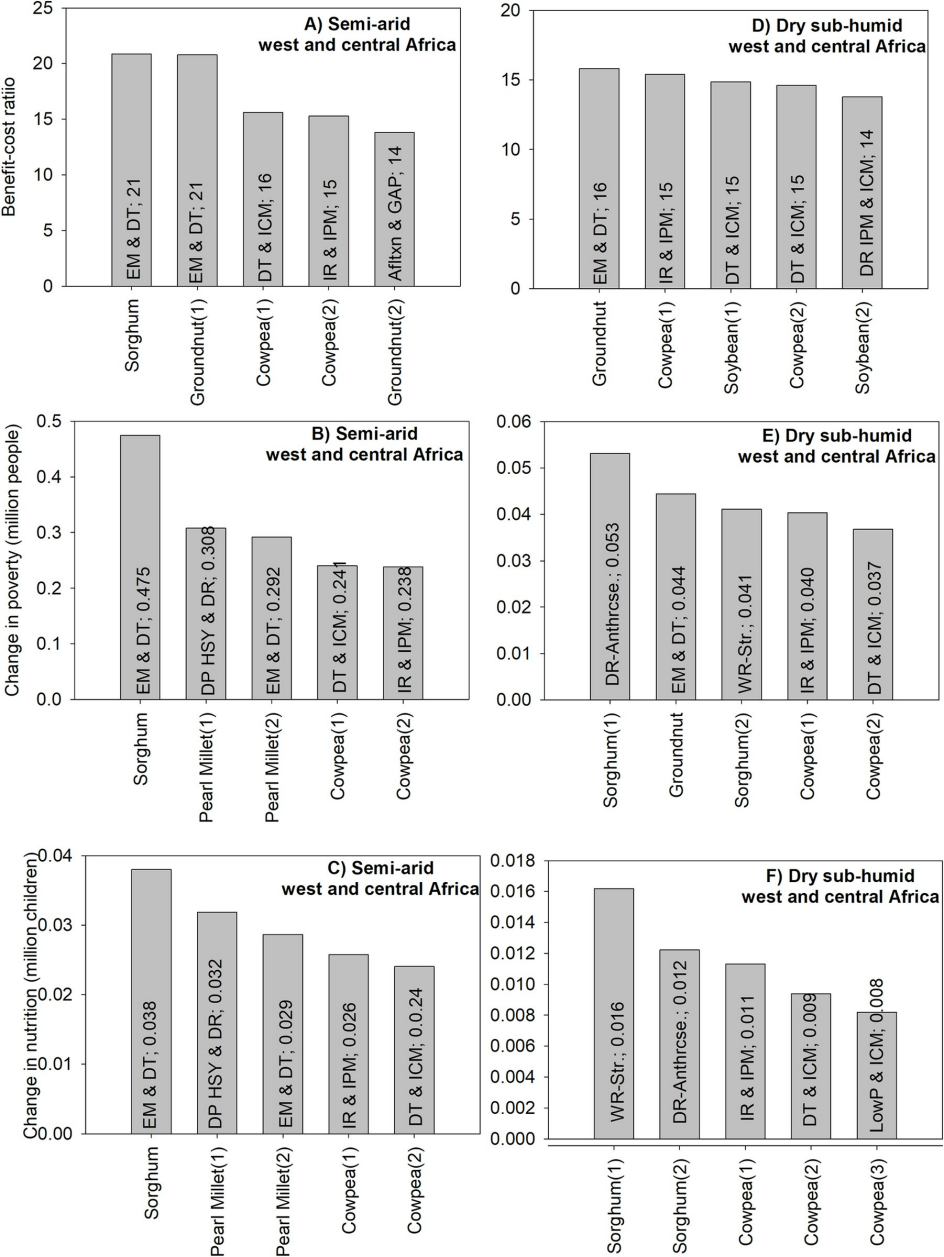
The five GLDC technologies with the highest impacts in west and central Africa. Source: authors’ computations. Note (alphabetical order): DP: dual-purpose; DR: disease resistance; DT: drought tolerance; EM: early maturity; HSY: high and stable yield; ICM: integrated crop management; IPM: integrated pest management; IR: insect resistance; LowP: Low P-tolerant varieties; WR-Anthrcse: resistance to anthracnose; WR-Str: resistance to Striga.

Based on the BCR criterion, the technology with the highest value in semi-arid West and Central Africa is drought-tolerant and early-maturing varieties of sorghum with a value of 21; this means that each dollar invested in generating and disseminating this improved technology would increase economic benefits by US$21. The other top technologies for BCR are related to groundnut and cowpea (Fig 2).

For poverty and nutrition security, the technology with the highest positive impacts also consists of drought-tolerant and early-maturing varieties of sorghum. The technology could reduce the number of poor people by about 475,000 (Fig 2) 10 years after being released (S1 Table); it also could reduce the number of malnourished children by about 38,000 (Fig 2). A comparison between the technologies ranked based on the BCR, poverty and nutrition security shows that three of the top five technologies linked to the BCR are also among the top five technologies linked to the other two criteria (Fig 2).

For dry sub-humid West and Central Africa, top technologies with high BCR are related to groundnut, cowpea and soybean (Fig 2). For the poverty criterion, the technologies with the highest positive impact are related to sorghum, groundnut and cowpea. For nutrition security, top technologies are related to sorghum and cowpea only (Fig 2).

The technology with the highest positive impact is not the same across all criteria for dry sub-humid west and central Africa. More specifically, early maturing and drought tolerant groundnut is the top technology for the BCR criterion; however, it is in second position for the poverty criterion and it’s not among the top five technologies for the nutrition security criterion. Despite this, two technologies are consistently among the top five across all criteria (Fig 2).

#### 3.1.2 Unidimensional ex ante impact assessment: Eastern Africa

**Fig 3:**
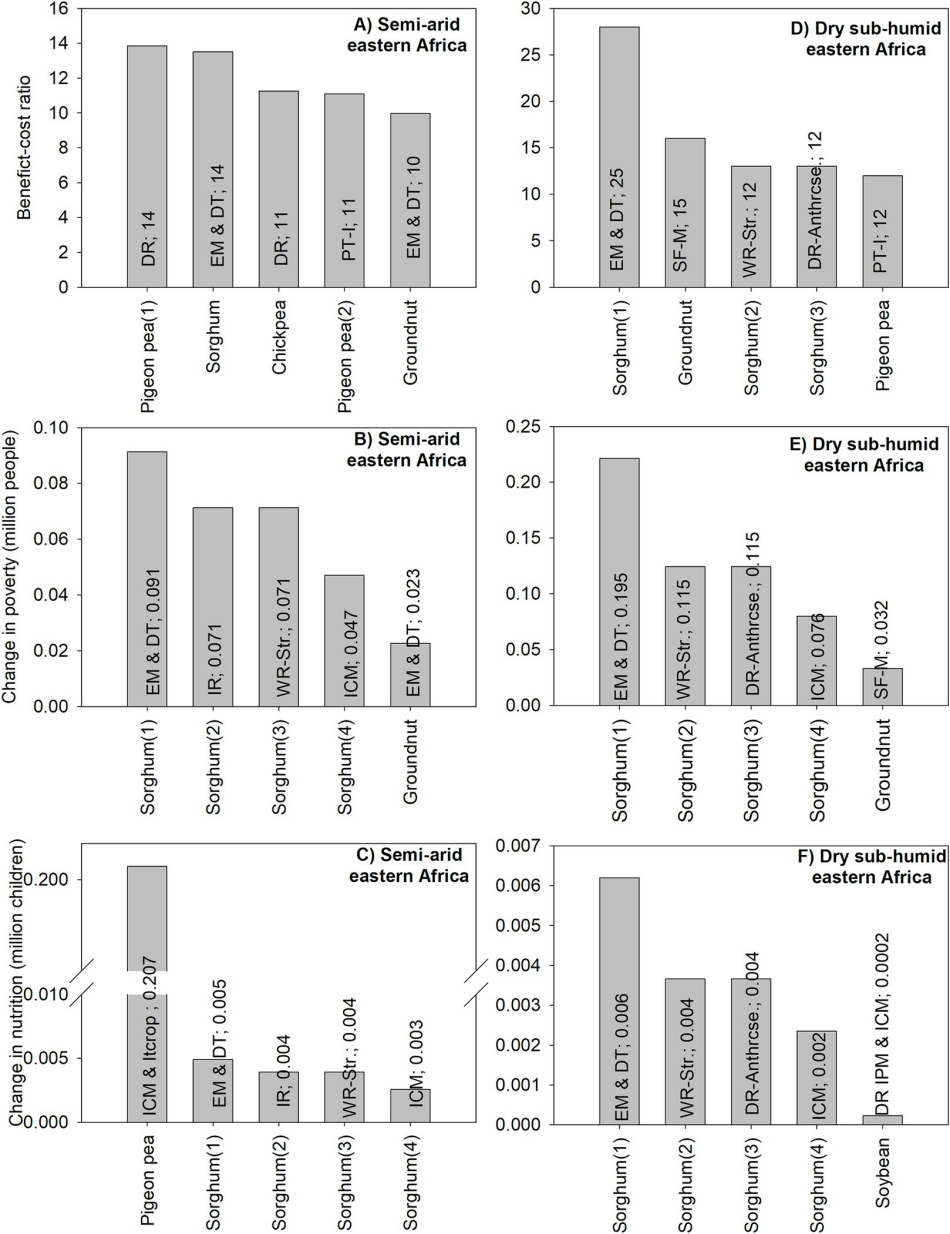
The five GLDC technologies with the highest positive impacts in eastern Africa. Source: authors’ computations. Note (alphabetical order): DR: disease resistance; DT: drought tolerance; EM: early maturity; ICM: integrated crop management; IPM: integrated pest management; IR: insect resistance; Itcrop: intercropping; PT-I: photo- and thermo-insensitive; PT-I: photo- and thermo-insensitive; SF-M: soil fertility management; WR-Anthrcse: resistance to anthracnose; WR-Str: resistance to Striga.

For semi-arid east Africa, the top two ranked technologies have a similar BCR, 14: one is related to sorghum and the other to pigeon pea. A comparison across all three criteria shows that early-maturing and drought-tolerant variety of sorghum is the top technology for both the benefit-cost ratio and poverty criteria; the technology is ranked second for the nutrition security criterion. In addition, the poverty and nutrition security criteria have in common four of the top five technologies (Fig 3).

For semi-arid east Africa, most technologies would increase the number of malnourished children (S11 Table) and such result reflects that some of the target technologies for this region do not involve staple food crops. For example, lentils do not figure among key staple crops for semi-arid east Africa. Hence, a new lentil technology would increase lentil yields and reduce per-unit production costs and make such crop more attractive for production. Hence, national acreage for lentil would increase to the detriment of the acreage of staple food crops; this could negatively affect nationwide food availability and nutrition security (Fig 3).

For dry sub-humid east Africa, the technology with the highest BCR would also have the highest positive impacts on poverty and nutrition security; it consists of early-maturing and drought-tolerant sorghum varieties and has a BCR of 25. The technology is also associated with a reduction of about 195 thousand people in the number of poor people and a reduction of about 6 thousand for the number of malnourished children. A comparison across all criteria shows similar rankings obtained with the poverty and nutrition security criteria (Fig 3).

#### 3.1.3 Unidimensional ex ante impact assessment: Southern Africa

**Fig 4:**
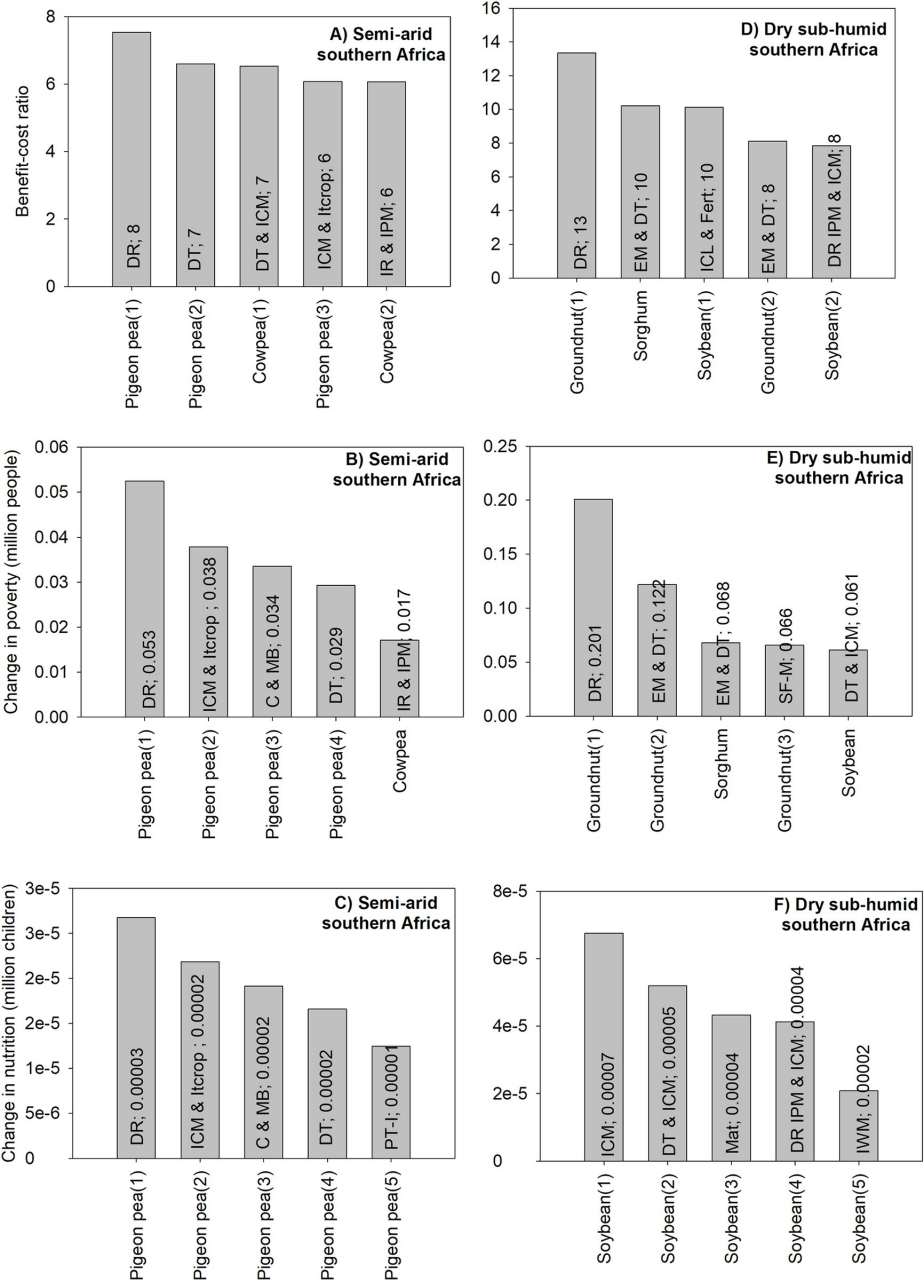
The five GLDC technologies with the highest positive impacts in southern Africa. Source: authors’ computations. Note (alphabetical order): C&MB: cleisto varieties and maintenance breeding; DR: disease resistance; DT: drought tolerance; EM: early maturity; ICL & Fert: Use of inoculant and fertilizers, especially Phosphorus; ICM: integrated crop management;; IPM: integrated pest management; IR: insect resistance; Itcrop: intercropping; IWM: integrated weed management; Mat: matching varieties with growing period; PT-I: photo- and hermos-insensitive; SF-M: soil fertility management.

For semi-arid southern Africa, the technology with the highest BCR would also have the highest positive impacts on poverty and nutrition security. The technology, disease-resistant pigeon pea, has a BCR of 8; it could lift about 53,000 people out of poverty and could reduce the number of malnourished children by about 30 (Fig 4).

A comparison across all criteria shows that two other technologies are found among the top five technologies for each criterion. They consist of drought-tolerant pigeon pea and integrated crop management combined with intercropping for pigeon pea. Also, the top four technologies based on the poverty criterion are identical to those related to the nutrition security criterion (Fig 4).

For dry sub-humid southern Africa, disease resistant varieties of groundnut, would have the highest positive impacts for BCR and poverty. The technology could increase economic benefits by about US$13 per dollar of investment in research and dissemination; it could also reduce the number of poor people by about 201,000. For nutrition security, all top five technologies involve only one crop, soybean (Fig 4).

A comparison across all three criteria shows that three technologies which are among the top five technologies for the benefit-cost ratio are also among the top 5 technologies for the poverty criterion (Fig 4).

#### 3.1.4 Unidimensional ex ante impact assessment: South Asia

**Fig 5:**
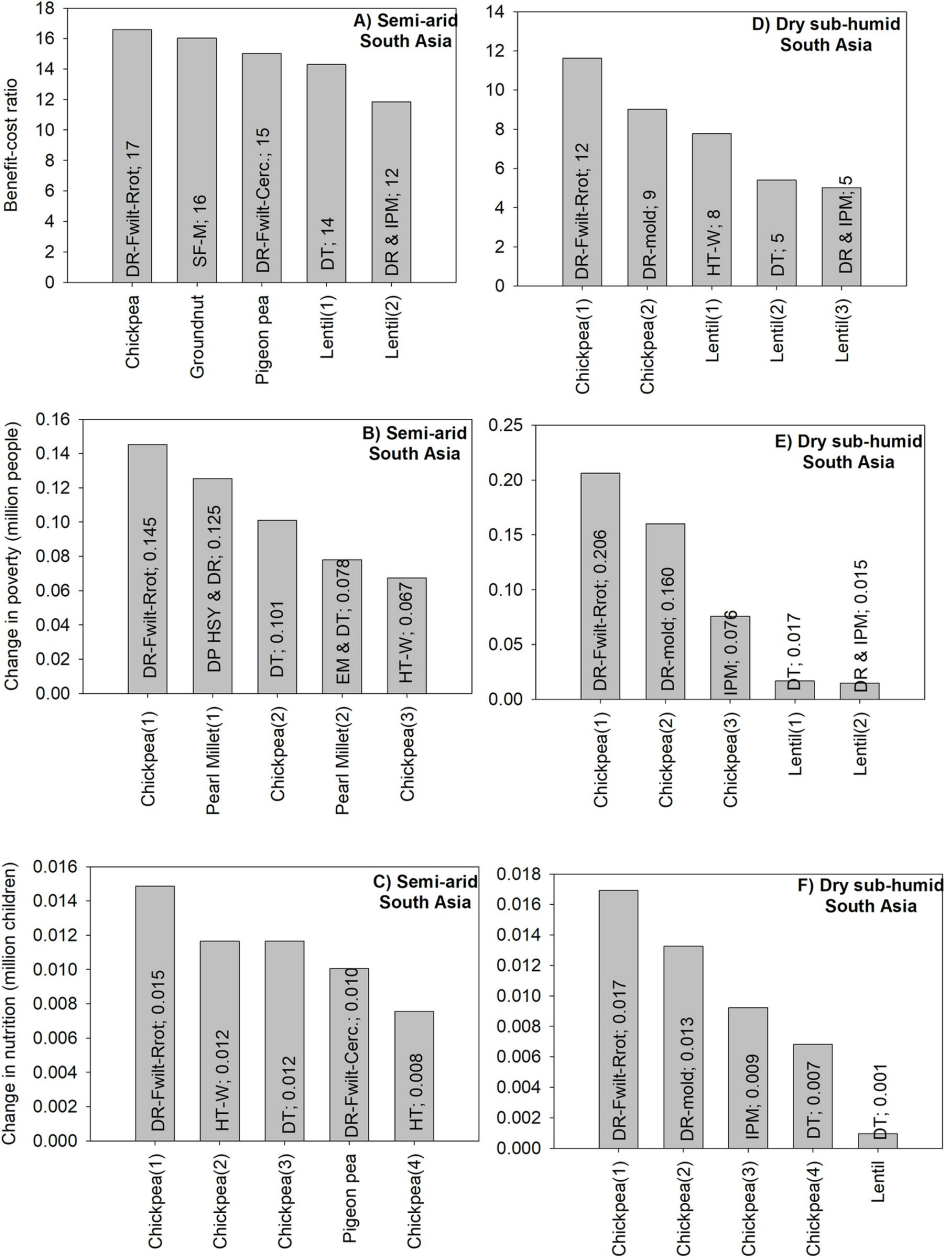
The five GLDC technologies with the highest positive impacts in South Asia. Source: authors’ computations. Note (alphabetical order): DP: dual-purpose; DR: disease resistance; DR-Fwilt-Rrot: Varieties resistant to Fusarium wilt and root rots; DR-mold: Botrytis gray mold-resistant varieties; DT: drought tolerance; HSY: high and stable yield; EM: early maturity; HT: heat tolerance; HT-W: herbicide tolerance for weed control; IPM: disease resistance and integrated pest management; SF-M: soil fertility management.

For semi-arid South Asia, the technology with the highest BCR would also have the highest positive impact on nutrition security; it consists of chickpea with resistance to Fusarium wilt and root rot. Such a technology could increase economic benefits by US$17 per dollar invested in research and dissemination (Fig 5). The technology is also number one for poverty and nutrition security. It could reduce the number of poor people by about 145000; it could also reduce the number of malnourished children by about 15,000. A comparison across all three criteria shows that all chickpea technologies which are among the top five technologies for poverty are also among the top five technologies for nutrition security. There is also a higher diversity of crops for the benefit-cost ratio compared to the other criteria (Fig 5).

For dry sub-humid south Asia, the technology with the highest benefit-cost ratio would also yield the highest benefits for poverty and nutrition security. Chickpea varieties resistant to Fusarium wilt and root rot would have the highest BCR with a value of 12. The technology could reduce the number of the number of poor people by about 206,000 and the number of malnourished children by 17,000. A comparison across all three criteria shows that the top two technologies are the same for all three criteria. In addition, for both poverty and nutrition security, the first three technologies are the same (Fig 5).

### 3.2 Multi-dimensional ex ante impact assessment of agricultural technologies: BCR, poverty, and child malnutrition

In this section, we apply TOPSIS to conduct the multi-dimensional ex ante impact assessment of promising technologies targeting the drylands of sub-Saharan Africa and South Asia.

#### 3.2.1 Multi-dimensional ex ante impact assessment: West and Central Africa

**Fig 6:**
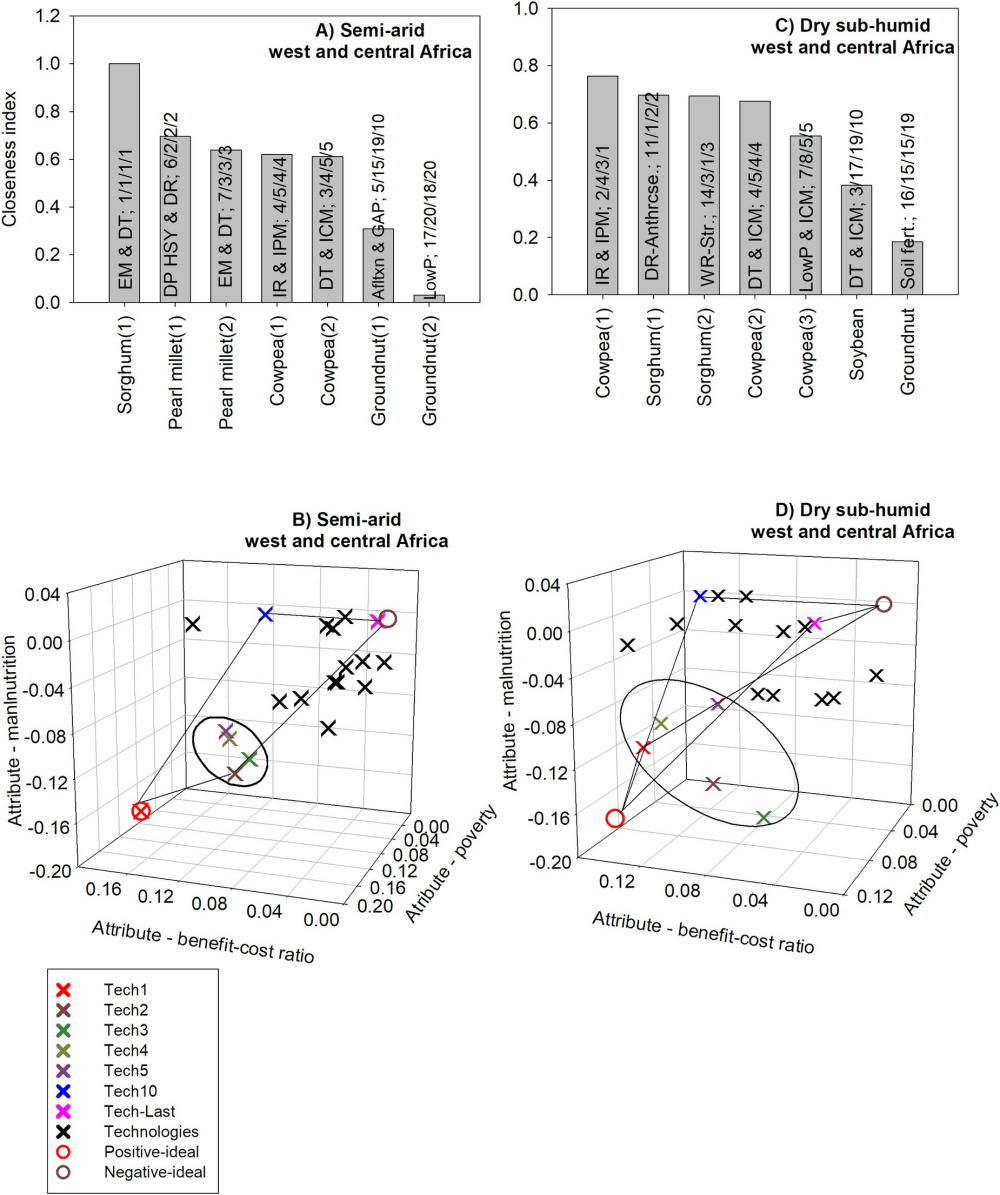
The five GLDC technologies with the highest positive multi-dimensional impacts in west and central Africa. Source: author’s computations. Note (alphabetical order): Afltxn & GAP: Aflatoxin management including Good Agricultural Practices; DP: dual-purpose; DR: disease resistance; DT: drought tolerance; EM: early maturity; HSY: high and stable yield; ICM: integrated crop management; IPM: integrated pest management; IR: insect resistance; LowP: Low P-tolerant varieties; Soil fert.: soil fertility management; WR-Anthrcse: resistance to anthracnose; WR-Str: resistance to Striga.

For semi-arid West and Central Africa, when considering the relative importance of the three criteria in the TOPSIS method, the technology with the highest closeness index consists of early-maturing and drought-tolerant varieties of sorghum. That technology was the number one technology for the BCR, poverty and child malnutrition criteria (Fig 6).

For this region, the BCR has the highest weight (39%) and both nutrition security (33%) and poverty (28%) have similar weights (Table 1); hence, all three criteria should matter for the multi-dimensional ranking of technologies, although the BCR should play a slightly more important role. For example, the technology ranked second for the BCR has a much lower impact on nutrition security and poverty compared to other technologies (S9 Table); it is ranked 15^th^ out of 20 for nutrition security and 9^th^ out of 20 for poverty (S9 Table). Hence, based on the closeness index, this technology is ranked sixth, and not second (S9 Table).

An analysis of the Euclidean distances between the top five technologies and the positive- and negative-ideal solutions shows that the technology consisting of early-maturing and drought-tolerant sorghum has the same coordinates as those for the positive-ideal solution for all three criteria. Such result reflects that this technology was ranked first for all three criteria. Apart from this technology, the other top four technologies based on the closeness index are also found to be the closest to the positive-ideal solution after the top technology for all three criteria. Similarly, the technology which was ranked last based on the closeness index is also the closest to the negative-ideal solution (Fig 6).

For dry sub-humid West and Central Africa, the technology with the highest closeness index consists of insect resistant cowpea combined with integrated pest management. This technology is ranked second based on the benefit-cost ratio criterion; it is ranked fourth and third based on poverty and child malnutrition criteria (Fig 6).

For dry sub-humid West and Central Africa, the benefit-cost ratio criterion has the highest weight (43%) followed by child malnutrition (33%) and then poverty (24%). One technology which consists of drought tolerance combined with integrated crop management for soybean is ranked 10^th^ out of 19 technologies based on the closeness index (Fig 6). This technology is ranked third based on the benefit-cost criterion; but it is ranked 17^th^ and last (19^th^) based on the poverty and child malnutrition criteria, respectively. Even though this technology is ranked highly for BCR, it cannot be among the top five technologies based on the closeness index since the latter are faring much better on nutrition security and poverty (Fig 6).

The technology which is ranked first based on the TOPSIS method is not the positive-ideal solution. Such result shows that this technology was not ranked first according to all three criteria. Similarly, the technology which is ranked last based on the TOPSIS method is the closest to the negative-ideal solution; but it is not the negative-ideal solution. This technology, which consists of soil fertility management for groundnut, is ranked 16^th^, 15^th^ and 15^th^, for the benefit-cost ratio, poverty and child malnutrition criteria, respectively (Fig 6a). If that technology was ranked 19^th^ for all three criteria, it would be the same as the negative-ideal solution.

#### 3.2.2 Multi-dimensional ex ante impact assessment: Eastern Africa

**Fig 7:**
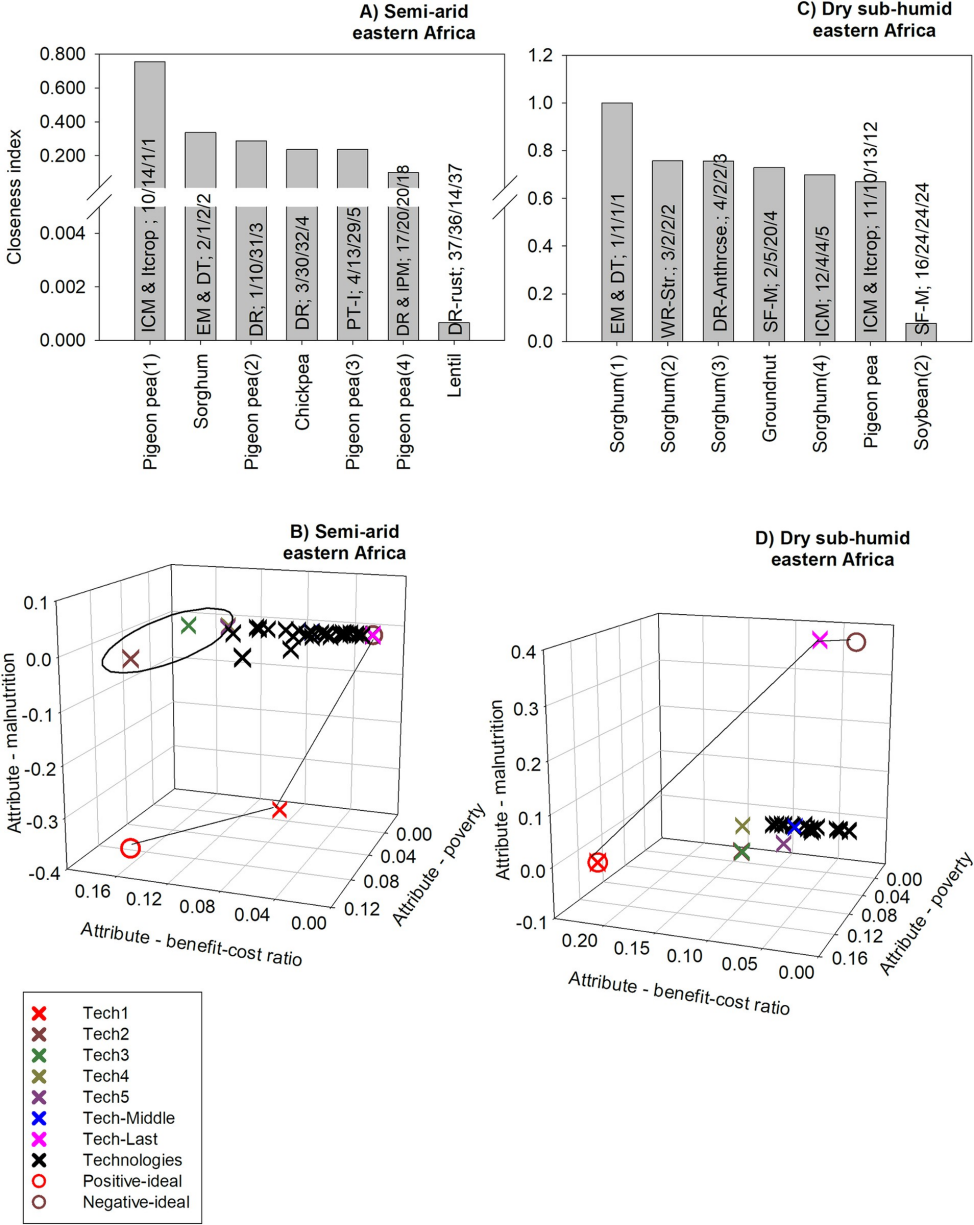
The five GLDC technologies with the highest positive multi-dimensional impacts in eastern Africa. Source: authors’ computations. Note (alphabetical order): DR: disease resistance; DT: drought tolerance; EM: early maturity; ICM: integrated crop management; IPM: integrated pest management; Itcrop: intercropping; PT-I: photo- and thermo-insensitive; SF-M: soil fertility management; Water Mng & Fert.: innovative water management and fertilizer regimes; WR-Anthrcse: resistance to anthracnose; WR-Str: resistance to Striga.

For semi-arid eastern Africa, the technology which is ranked first based on the closeness index consist of intercropping compatible varieties of pigeon pea combined with integrated crop management. This technology is ranked 10^th^, 14^th^ and 1^st^ based on the benefit-cost ratio, poverty, and child malnutrition criteria, respectively (Fig 7). Given that the benefit-cost ratio has the highest weight (47%) among all three criteria, it plays a more important role in the multi-dimensional ranking of the technologies. For example, the third and fourth best technologies based on the closeness index are ranked 1^st^ and 3^rd^ based on the benefit-cost criterion alone. Two technologies are ranked last (rank 37) based on the closeness index. They consist of disease-resistant (DR) varieties of lentil; one set of technologies involves rust resistance and the other involves resistance to Ascochyta blight (S11 Table). The two technologies are ranked last according to the benefit-cost ratio and before last for poverty; they are ranked 14th based on the child malnutrition criterion (S11 Table).

An analysis of the Euclidean distances to the positive and negative-ideal solutions reveals that most technologies are clustered around the lowest index for poverty and the highest index value for child malnutrition. This means that these technologies tend to have low impacts on poverty and nutrition security. Also, the technology which is ranked first based on the closeness index is quite far from the positive-ideal solution. This technology has the best index value for child malnutrition (lowest), but its index value for poverty is closer to zero, whereas its index value for the benefit-cost ratio criterion is about 0.10. The two technologies ranked last based on the closeness index have a closeness index that is almost zero (S11 Table); the negative-ideal solution has a closeness index of zero. Hence, these last two technologies do not perfectly match with the negative-ideal solution (Fig 7).

For dry sub-humid eastern Africa, the technology with the highest closeness index based on the closeness index consists of early-maturing and drought-tolerant varieties of sorghum. This technology is ranked first for all three criteria (Fig 7). Given that the benefit-cost ratio (43%) and child malnutrition (38%) criteria have high and similar weights for this region, the top-ranked technologies tend to have high ranks for the benefit-cost ratio and/or child malnutrition (Fig 7). The fifth technology based on the closeness index consists of integrated crop management (ICM) for sorghum; that technology is ranked 12^th^ based on the BCR and 4^th^ for each of poverty and nutrition security. The fourth technology based on the closeness index consists of soil fertility management for groundnut. This technology is ranked second for BCR, but 20^th^ and 5^th^ for nutrition security and poverty, respectively. The higher weight for the BCR explains why such technology is fourth based on the closeness index and is ahead of the technology consisting of integrated crop management (ICM) for sorghum (Fig 7).

Not surprisingly, the technology that is ranked first based on the TOPSIS method is the same as the positive-ideal solution. The technology ranked last based on the TOPSIS method consists of soil fertility management for soybean. This technology is closest to the negative-ideal solution and it is associated with a high positive index for child malnutrition; this result implies that the technology would substantially increase the number of malnourished children in the region. Such result reflects that soybean is not a key staple in the region (Fig 7).

#### 3.2.3 Multi-dimensional ex ante impact assessment: Southern Africa

**Fig 8:**
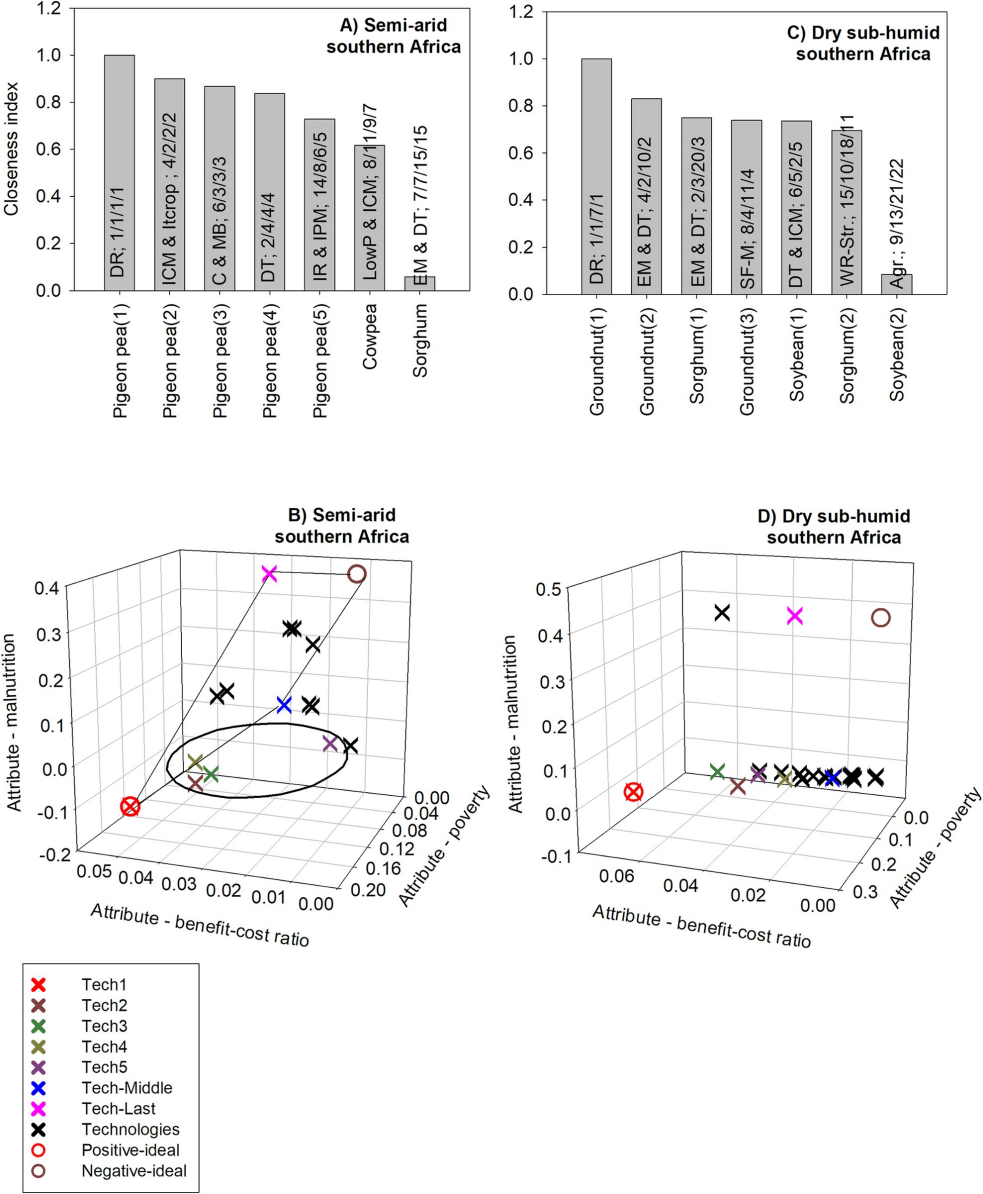
The five GLDC technologies with the highest positive multi-dimensional impacts in southern Africa. Source authors’ computations. Note (alphabetical order): Agr: optimum planting window; C&MB: cleisto varieties and maintenance breeding; DR: disease resistance; DR: disease resistance; DT: drought tolerance; DT: drought tolerance; IR: insect resistance; EM: early maturity; ICM: integrated crop management; ICM: integrated crop management; IPM: integrated pest management; Itcrop: intercropping; LowP: Low P-tolerant varieties; SF-M: soil fertility management; WR-Str: resistance to Striga.

For semi-arid southern Africa, the technology which is ranked first based on the closeness index consists of disease-resistant pigeon pea. Such a technology is also ranked first based on the benefit-cost ratio, poverty, and child malnutrition criteria. For this region, child malnutrition has a weight of around 58% compared to 29% for poverty and 13% for the benefit-cost ratio. Hence, nutrition security plays an important role in the ranking of technologies based on the closeness index (Fig 8). As a result, all technologies ranked based on the closeness index closely follow the ranking based on nutrition security only (S13 Table).

For semi-arid southern Africa, the technology with the highest closeness index is the same as the positive-ideal solution (Fig 8) based on Euclidean distances. The technology which is ranked last (15^th^) based on the closeness index is not the same as the negative-ideal solution since it is not ranked last based on BCR and poverty (it’s only ranked last for child malnutrition). For semi-arid southern Africa, some technologies have positive index values for child malnutrition and such results also reflect that some of the proposed technologies for this region would worsen child malnutrition (Fig 8).

For dry sub-humid southern Africa, the technology with the highest closeness index consists of disease-resistant groundnut. This technology was ranked first based on the benefit-cost ratio and poverty criteria; it is ranked 7^th^ according to the child malnutrition criterion. Here too, the child malnutrition criterion has a high weight (55%) compared to the poverty (29%) and benefit-cost ratio (15%) criteria, respectively. However, most technologies have a slight and similar impact on child malnutrition (S14 Table); hence, poverty is the defining criterion in ranking the technologies. As a result, the top technologies tend to have a high ranking for poverty (Fig 8). The technology which is ranked last (22^nd^) consists of improved agronomic practices for soybean (Fig 8).

The analysis of Euclidean distances to the positive- and negative-ideal solutions shows that most technologies are clustered around an index value of zero for each of the poverty and child malnutrition criterion. Only two technologies, including the one ranked last based on the TOPSIS method, have a high positive index value for child malnutrition; such result suggests that these technologies would substantially worsen child malnutrition. The technology which is ranked last is not identical to the negative-ideal solution because this technology is not last for all three criteria. The technology which is ranked first based on the TOPSIS method does not have a closeness index of 1 since it is ranked 7^th^ based on the child malnutrition criterion. Hence, it does not fit perfectly with the positive-ideal solution (Fig 8).

#### 3.2.4 Multi-dimensional ex ante impact assessment: South Asia

**Fig 9:**
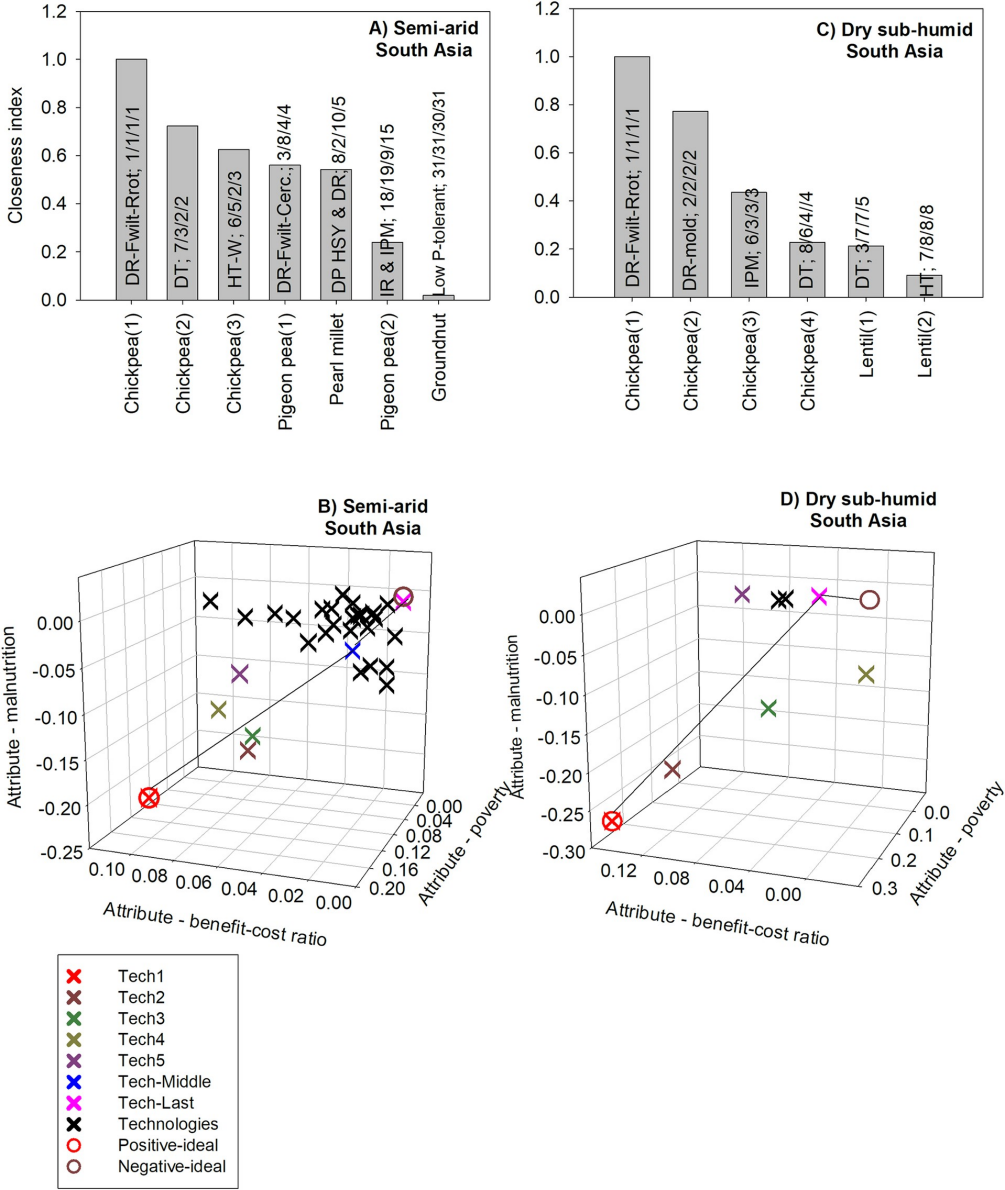
The five GLDC technologies with the highest positive multi-dimensional impacts in South Asia. Source: authors’ computations. Note (alphabetical order): DP: dual-purpose; DR: disease resistance; DR-Fwilt-Cerc: varieties resistant to Fusarium wilt and Cercospora leaf spot; DR-Fwilt-Rrot: varieties resistant to Fusarium wilt and root rots; DR-mold: Botrytis gray mold-resistant varieties; DT: drought tolerance; HSY: high and stable yield; HT: heat tolerance; HT-W: herbicide tolerance for weed control; ICM: integrated crop management; IPM: integrated pest management; Itcrop: intercropping; Low P-tolerant: low P-tolerant variety.

For semi-arid South Asia, the technology with the highest closeness index consists of disease-resistant chickpea. This technology is ranked first for all three criteria. For this region, child malnutrition and poverty have similar weights whereas the benefit-cost ratio has the lowest weight of about 26%. Hence the top-ranked technologies based on the TOPIS method tend to have high rankings for child malnutrition and poverty (Fig 9).

The Euclidean distances to the positive- and negative-ideal solutions show that the technology ranked first based on the TOPSIS method is also the positive-ideal solution. Here, the technology ranked last based on the TOPSIS method is ranked last for the BCR and poverty criteria; it’s ranked before last for child malnutrition. Hence, this technology is not identical to the negative-ideal solution (S15 Table). The middle-ranked technology is closer to the negative-ideal solution than it is to the positive-ideal solution (Fig 9).

For dry sub-humid south Asia, the technology which is ranked first based on the closeness index consists of disease-resistant chickpea (Fig 9). This technology is also ranked first based on the benefit-cost, poverty and child malnutrition criteria. For this region, nutrition security (39%) and poverty (37%) have similar weights, whereas the BCR has a weight of about 24%. Hence, the ranking of technologies based on the closeness index follows closely that for nutrition security and poverty. More specifically, the first four technologies based on the closeness index are the same as those for nutrition security. The next 3 technologies based on the closeness index have similar values of nutrition security; hence, the poverty criterion demarcates these technologies. The technology ranked last based on the closeness index also has the last ranking for both nutrition security and poverty (S16 Table).

The analysis of Euclidean distances to the positive- and negative-ideal solutions shows that the technology that is ranked first based on the closeness index also corresponds to the positive-ideal solution. However, the technology that is ranked last (8^th^) based on the closeness index is not the same as the negative-ideal solution, although it is closest to it. That technology consists of heat-tolerant lentil; it is ranked last based on the poverty and child malnutrition criteria, respectively; but it is ranked 7^th^ based on the benefit-cost criterion (Fig 9).

## 4 Discussion

The ex-ante impact of agricultural technologies has usually been conducted for specific criteria in a target environment. This study shows that with TOPSIS, an additional layer is considered, and technologies can be ranked on a multi-dimensional basis where multiple criteria are considered simultaneously. The study results show that promising technologies vary across regions based on the following factors: the magnitude of the benefit cost ratio linked to a specific technology; the magnitude of the people lifted out of poverty from the technology; the magnitude of the children lifted from malnutrition after the adoption of the technology; and the importance of each criterion (BCR, poverty, nutrition security) in the target region. For example, in the drylands of west and central Africa, agriculture’s contribution to GDP and child stunting are more important compared to poverty. Hence, the multi-dimensional ranking of technologies for this mega-environment prioritizes technologies with greater positive impacts on BCR and nutrition security. More specifically, drought tolerance for sorghum, millet, cowpea and groundnut would have the highest positive multi-dimensional impacts in semi-arid West and Central Africa. For dry sub-humid West and Central Africa, technologies with the highest positive multi-dimensional impact involve mainly cowpea and sorghum. Impact assessment studies in these two regions have usually focused on individual criteria and are primarily ex-post. The studies have shown that improved cowpea varieties have reduced poverty in Nigeria [55], whereas improved cowpea storage techniques have experienced an increase in adoption in western Africa, thus suggesting an improvement in nutrition security in the region [56]. Impact assessment studies dealing with other crops in this region have focused on soybean, groundnut, and millet [57, 58].

In eastern Africa, the BCR and nutrition security also have higher weights compared to the poverty criterion. Hence, for dry sub-humid eastern Africa, the technologies with the highest positive multi-dimensional impact primarily have more pronounced impacts on the BCR and nutrition security; they involve sorghum and soybean. For semi-arid eastern Africa, the best technologies are mainly related to pigeon pea. Past impact assessment studies for eastern Africa have usually focused on individual criteria, be it poverty or nutrition security. In addition, most studies have not assessed multiple technologies involving multiple crops. Nevertheless, one study that analyzed the impact of a 25% yield increase for both sorghum and millet in eastern and southern Africa found that sorghum improvement would generate higher benefits on poverty and food security than millet improvement [57]. In our study, sorghum and millet technologies were proposed for dry-sub-humid eastern Africa only and the results from applying the TOPSIS method show that sorghum technologies usually outweigh millet technologies in terms of multi-dimensional impact.

In southern Africa, nutrition security has a weight of more than 50%. Hence, TOPSIS prioritizes technologies that have a higher positive impact on nutrition security. For semi-arid southern Africa, technologies with the highest multi-dimensional impact primarily involve pigeon pea. For dry sub-humid southern Africa, drought tolerance for three crops namely groundnut, sorghum and soybean would have the highest positive multi-dimensional impact. The few impact assessment studies conducted for grain legumes and dryland cereals for countries in southern Africa have mainly focused on grain legumes and more specifically on soybean, groundnut, and cowpea [59–63].

For south Asia, nutrition security followed by poverty have higher weights compared to the BCR criterion. For semi-arid South Asia, disease resistance for chickpea, pigeon pea and pearl millet would have the highest positive multi-dimensional impact. For dry sub-humid south Asia, technologies with the greatest potential multi-dimensional impact involve chickpea or lentil. Most impact assessment studies for technologies related to grain legumes and dryland cereals for countries in South Asia have targeted only farm income as a criterion [57].

The study results show that improved agricultural technologies can generate substantial benefits. The results should guide researchers to prioritize breeding efforts given limited resources. However, as shown in various studies, agricultural technologies alone might not be enough to bring about substantial welfare improvements. Additional policy measures that promote market access or enhance credit access could further enhance nutrition security or generate high economic benefits [59, 64, 65].

In this study, criteria weights for TOPSIS were identified based on the relative importance of each criterion in a target region. Criteria weights play an important role in the identification of promising technologies. However, sensitivity analysis can be conducted to assess how the multi-dimensional ranking of the technologies would change with different weights. For example, if all criteria are assumed to have equal weights in each target country to reflect that these three criteria have the same importance, the ranking of technologies would change to reflect this. A recent application, TOPSIS_ShinyApp, was developed for such purpose [66]; the app uses pre-computed results for each criterion and then applies the TOPSIS method with pre-identified weights to provide a multi-dimensional ranking of promising technologies. The usefulness of the app lies in its ability to save time for researchers. More specifically, the tool can combine multiple criteria at a go; in addition, the app allows one to easily conduct sensitivity tests and see how differing weights influence the ranking of agricultural technologies. This app could be improved in the future to use machine learning in determining criteria weights.

## 5 Conclusion

This study applies the TOPSIS method for ex-ante impact assessment of agricultural technologies, which play a multidimensional role in the dryland regions where they matter. The agricultural technologies assessed in this study are important for food and nutrition security in the drylands of sub-Saharan Africa and south Asia; they are also important in natural resource management in addition to being an important source of livelihood. For this study, three outcome indicators were used to evaluate GLDC technology options: BCR, poverty and child malnutrition. Two steps were applied to assess the multi-dimensional impact of these technologies. The first step involved quantifying the impacts of these technologies on the outcome indicators and the second step involved applying the TOPSIS method to obtain a multi-dimensional ranking of the technologies. The results show that technologies with the highest positive multi-dimensional impacts vary across agro-ecologies partly based on the importance of each outcome indicator in the target regions. For example, in semi-arid west Africa, where child stunting and agriculture as a source of livelihood are more prominent, drought tolerance for sorghum, millet, cowpea, and groundnut would have the highest positive multi-dimensional impacts. Cowpea and groundnut are highly nutritious, and all four crops are highly traded in the region. However, in semi-arid southern Africa, nutrition security is more important compared to the BCR and poverty. Hence, technologies with the highest positive multi-dimensional impacts mainly involve pigeon pea, a highly nutritious crop which is also consumed in the region. The results from this study are important in supporting priority-setting of agricultural technologies. The study also provides a practical framework which can be used by socio-economic researchers to quantify the multi-dimensional impact of improved agricultural technologies. One study limitation is the focus on only two dimensions which are affected by agricultural technologies: welfare and nutritional security. Future studies targeting the drylands of SSA and South Asia should apply the TOPSIS method to consider the other important roles that dryland cereals and grain legumes technologies play on food security and on the environment (soil fertility and climate change mitigation).

## Supporting information

S1 Table Research, dissemination and adoption parameters for improved technologies—semi-arid West and Central Africa. Tech: 1: Drought-tolerant varieties and integrated crop management; 2: Insect- (aphid, thrips, pod sucking bug, maruca) resistant lines and integrated pest management including biological control; 3: Disease-resistant varieties and integrated crop management; 4: Striga-resistant varieties and integrated crop management; 5: Low P-tolerant varieties and integrated crop management; 6: Drought-tolerant/resistant variety and short-duration (early-maturing) variety; 7: Moderately-resistant variety (for short-duration variety) and highly- resistant variety (for medium- and long-duration varieties) to early and late leaf spot; 8: Rosette-resistant variety; 9: Low P-tolerant/efficient variety; 10: Pre and postharvest aflatoxin management practices including Good Agricultural Practices (GAP); 11: Soil fertility management for P and other nutrients (N, Ca) including chemical/organic fertilizers application; 12: Early-maturing, drought-tolerant hybrids which can give stable yields under severe drought conditions; 13: Genetically diverse dual-purpose hybrid parents/cultivars with high and stable yields with disease resistance (downy mildew and blast); 14: Biological control of millet head miner and resistant hybrid parents; 15: Integrated soil fertility management and identifying genotypes for low P tolerance; 16: OPVs with host plant resistance to Striga hermonthica; 17: Early-maturing varieties and hybrids with tolerance to drought; 18: Striga-resistant varieties and hybrids; 19: Cultivars adapted to low soil fertility/and with nutrient-use efficiency; 20: Stem borer/midge-tolerant cultivars.
(DOCX)

S2 Table Research, dissemination and adoption parameters for improved technologies–dry sub-humid West and Central Africa. Tech: 1: Striga-resistant varieties and integrated crop management; 2: Disease-resistant varieties and integrated crop management; 3: Drought-tolerant varieties and integrated crop management; 4: Insect- (aphid, thrips, pod sucking bug, maruca) resistant lines and integrated pest management including biological control; 5: Low P-tolerant varieties and integrated crop management; 6: Drought-tolerant/resistant variety and short-duration (early- maturing) variety; 7: Increased plant population; 8: Moderately-resistant variety (for short-duration variety) and highly-resistant variety (for medium- and long-duration varieties) to early and late leaf spot; 9: Pre and postharvest aflatoxin management practices including Good Agricultural Practices (GAP); 10: Rosette-resistant variety; 11: Soil fertility management for P and other nutrients (N, Ca) including chemical/organic fertilizers application; 12: Head bugs- and grain mold-tolerant cultivars; 13: Medium- to late-maturing anthracnose-resistant cultivars; 14: Striga-resistant varieties and hybrids; 15: Cultivars adapted to low soil fertility/and with nutrient-use efficiency; 16: Stem borer/midge-tolerant cultivars; 17: Drought-tolerant varieties and crop management and water conservation practices; 18: Disease-resistant varieties and integrated pest management and crop management practices; 19: Use of inoculant and fertilizers especially Phosphorus.
(DOCX)

S3 Table Research, dissemination and adoption parameters for improved technologies–semi-arid eastern Africa. * Fgr millet = Finger millet; Tech: 1: Ascochyta blight-resistant varieties; 2: Drought-tolerant varieties; 3: Fusarium wilt- and root rots-resistant varieties; 4; Waterlogging-tolerant varieties and management practices; 5: Striga-resistant varieties and integrated crop management; 6: Disease-resistant varieties and integrated crop management; 7: Drought-tolerant varieties and integrated crop management; 8: Insect- (aphid, thrips, pod sucking bug, maruca) resistant lines and integrated crop management; 9: Low P-tolerant varieties and integrated crop management; 10: Downy mildew- and smut-resistant dual-purpose OPVs and hybrid parents; 11: OPVs with host plant resistance to Striga hermonthica; 12: Early-maturing, drought-tolerant OPVs and hybrids which can give stable yields under severe drought conditions; 13: Varieties and hybrid parents with good establishment and that respond well to drought, especially terminal drought; 14: Validate and promote water management options; fertilizer regimes; 15: Drought-tolerant/resistant variety and short-duration (early-maturing) variety; 16: Low P-tolerant/efficient variety; 17: Moderately-resistant variety (for short-duration variety) and highly-resistant variety (for medium- and long-duration varieties) to early and late leaf spot; 18: Pre and postharvest aflatoxin management practices including Good Agricultural Practices (GAP); 19: Rosette-resistant variety; 20: Soil fertility management for P and other nutrients (N, Ca) including chemical/organic fertilizers application; 21: Ascochyta blight-resistant varieties; 22: Drought-tolerant varieties; 23: Rust-resistant varieties; 24: Weed management; 25: Genetically diverse dual-purpose hybrid parents/cultivars with high and stable yields with disease resistance (downy mildew and blast); 26: Early-maturing, drought-tolerant hybrids which can give stable yields under severe drought conditions; 27: Varieties and hybrid parents with good establishment and respond well to drought especially terminal drought; 28: Validate and promote water management options; fertilizer regimes; 29: Cleisto varieties and maintenance breeding to reduce varietal degeneration due to out crossing; 30: Drought-tolerant varieties; 31: Fusarium wilt- and Cercospora leaf spot-resistant varieties; 32: Intercropping compatible-varieties and integrated crop management options; 33: Varieties tolerant to pod borers, pod fly, pod bugs and integrated pest management; 34: Photo- and thermo-insensitive varieties; 35: early-maturing varieties and hybrids with tolerance to drought; 36: Integrated crop management options for soil fertility, water management, Striga, intercropping; 37: Stem borer/midge-tolerant cultivars; 38: Striga-resistant varieties and hybrids.
(DOCX)

S4 Table Research dissemination and adoption parameters for improved technologies–dry sub-humid eastern Africa. Tech: 1: Alectra-resistant varieties and integrated crop management; 2: Disease-resistant varieties and integrated crop management; 3: Drought-tolerant varieties and integrated crop management; 4: Lines resistant to insects (aphid, thrips, pod sucking bug, maruca) and integrated pest management including biological control; 5: Low P-tolerant varieties and integrated crop management; 6: Drought-tolerant/resistant variety and short-duration (early- maturing) variety; 7: Moderately-resistant (for short-duration variety) and highly- resistant variety (for medium- and long-duration varieties) to early and late leaf spot; 8: Pre and postharvest aflatoxin management practices including Good Agricultural Practices (GAP); 9: Rosette-resistant variety; 10: Soil fertility management for P and other nutrients (N, Ca) including chemical/organic fertilizers application; 11: Varieties resistant to Fusarium wilt and Cercospora leaf spot; 12: Cleisto varieties and maintenance breeding to reduce varietal degeneration due to outcrossing; 13: Drought-tolerant varieties; 14: Weed control; 15: Intercropping-compatible varieties and integrated crop management options; 16: Varieties tolerant to pod borers, pod fly, pod bugs and integrated pest management; 17: Photo- and thermo-insensitive varieties; 18: Varieties tolerant to warm temperatures; 19: Early-maturing varieties and hybrids with tolerance to drought; 20: Integrated crop management options for soil fertility, water management, Striga, intercropping; 21: Medium- to late-maturing anthracnose-resistant cultivars; 22: Varieties and hybrids with resistance to Striga; 23: Integrated soil fertility management; 24: Disease-resistant varieties and integrated pest management and crop management practices.
(DOCX)

S5 Table Research dissemination and adoption parameters for improved technologies–semi-arid southern Africa. Tech: 1: Alectra-resistant varieties and integrated crop management; 2: Disease-resistant varieties and integrated crop management; 3: Drought-tolerant varieties and integrated crop management; 4: Lines resistant to insects (aphid, thrips, pod sucking bug, maruca) and integrated pest management including biological control; 5: Low P-tolerant varieties and integrated crop management; 6: Cleisto varieties and maintenance breeding to reduce varietal degeneration due to outcrossing; 7: Drought-tolerant varieties; 8: Varieties resistant to Fusarium wilt and Cercospora leaf spot; 9: Intercropping-compatible varieties and integrated crop management options; 10: Photo- and thermo-insensitive varieties; 11: Varieties tolerant to pod borers, pod fly, pod bugs and integrated pest management; 12: Early-maturing varieties and hybrids with tolerance to drought; 13: Integrated crop management options for soil fertility, water management, Striga, intercropping; 14: Medium- to late-maturing anthracnose-resistant cultivars; 15: Varieties and hybrids with resistance to Striga.
(DOCX)

S6 Table Research dissemination and adoption parameters for improved technologies–dry sub-humid southern Africa. Tech: 1: Drought-tolerant varieties and integrated crop management; 2: Lines resistant to insects (aphid, thrip, pod sucking bug, maruca) and integrated crop management; 3: Disease-resistant varieties and integrated crop management; 4: Striga and Alectra-resistant varieties and integrated crop management; 5: Low P-tolerant varieties and integrated crop management; 6: Rosette-resistant variety; 7: Moderately-resistant variety (for short-duration variety) and highly-resistant variety (for medium- and long-duration varieties) to early and late leaf spot 8: Drought-tolerant/resistant variety and short-duration (early- maturing) variety; 9: Low P-tolerant/efficient variety; 10: Pre and postharvest aflatoxin management practices including Good Agricultural Practices (GAP); 11: Soil fertility management for P and other nutrients (N, Ca) including chemical/organic fertilizers application; 12: Early-maturing varieties and hybrids with tolerance to drought; 13: Integrated crop management options for soil fertility, water management, Striga, intercropping; 14: Medium- to late-maturing anthracnose-resistant cultivars; 15: Varieties and hybrids with resistance to Striga; 16: Drought-tolerant varieties and crop management and water conservation practices; 17: Establish optimum planting window and awareness creation; 18: Use of inoculant and fertilizers, especially Phosphorus; 19: Use of good quality seed, appropriate seeding rate and row spacing; 20: Disease-resistant varieties and integrated pest management and crop management practices; 21: Matching varieties that fit the growing period; 22: Breeding for resistance to parasitic weeds and integrated weed management.
(DOCX)

S7 Table Research dissemination and adoption parameters for improved technologies–semi-arid South Asia. Tech: 1: Varieties resistant to Fusarium wilt and root rots; 2: Drought-tolerant varieties; 3: Heat-tolerant varieties; 4: Pod borer-tolerant varieties and integrated pest management; 5; Herbicide-tolerant varieties to control weeds; 6: Genetically diverse dual-purpose hybrid parents/cultivars with high and stable yields; 7: Breeding for early-maturing, drought-tolerant OPVs and hybrids which can give stable yields under severe drought conditions; 8: Breeding for downy mildew- and smut-resistant dual-purpose OPVs and hybrid parents; 9: Integrated crop management; 10: Varieties resistant to diseases (foliar fungal, bud necrosis, soil borne); 11: Integrated crop management practices; 12: Low P-tolerant/efficient variety; 13: Pre and postharvest aflatoxin management practices including Good Agricultural Practices (GAP); 14: Soil fertility management for P and other nutrients (N, Ca) including chemical/organic fertilizers application; 15: Varieties resistant to wilt and root rots and integrated pest management; 16: Drought-tolerant varieties; 17: Heat-tolerant varieties; 18: Herbicide-tolerant varieties to control weeds; 19: Genetically diverse dual-purpose hybrid parents/cultivars with high and stable yields with disease resistance (downy mildew and blast); 20: Breeding for early-maturing, drought-tolerant hybrids which can give stable yields under severe drought conditions; 21: Integrated crop management; 22: Varieties resistant to Fusarium wilt and Cercospora leaf spot; 23: Varieties tolerant to pod borers, pod fly, pod bugs and integrated pest management; 24: Sterility mosaic disease-resistant varieties; 25: Intercropping compatible-varieties and integrated crop management options; 26: Drought-tolerant varieties; 27: Genetic base diversification; 28: Early-maturing varieties and hybrids with tolerance to drought; 29: Shoot fly-resistant cultivars; 30: Charcoal rot-resistant cultivars; 31: Cultivars tolerant to head bugs and grain mold.
(DOCX)

S8 Table Research dissemination and adoption parameters for improved technologies–dry sub-humid South Asia. Tech: 1: Botrytis gray mold-resistant varieties; 2: Varieties resistant to Fusarium wilt and root rots; 3: Pod borer-tolerant varieties and integrated pest management; 4: Drought-tolerant varieties; 5: Stemphylium blight-resistant varieties and integrated pest management; 6: Herbicide-tolerant varieties to control weeds; 7: Drought-tolerant varieties; 8: Heat-tolerant varieties.
(DOCX)

S9 TableEstimated closeness index and ranking of technologies in semi-arid west and central Africa.Tech: 1: Early-maturing varieties and hybrids with tolerance to drought; 2: Genetically diverse dual-purpose hybrid parents/cultivars with high and stable yields with disease resistance (downy mildew and blast); 3: Early-maturing, drought-tolerant hybrids which can give stable yields under severe drought conditions; 4: Insect- (aphid, thrips, pod sucking bug, maruca) resistant lines and integrated pest management including biological control; 5: Drought-tolerant varieties and integrated crop management; 6: Drought-tolerant/resistant variety and short-duration (early-maturing) variety; 7: Low P-tolerant varieties and integrated crop management; 8: Biological control of millet head miner and resistant hybrid parents; 9: Striga-resistant varieties and hybrids; 10: Pre and postharvest aflatoxin management practices including Good Agricultural Practices (GAP); 11: Striga-resistant varieties and integrated crop management; 12: Disease-resistant varieties and integrated crop management; 13: Integrated soil fertility management and identifying genotypes for low P tolerance; 14: Stem borer/midge-tolerant cultivars; 15: Cultivars adapted to low soil fertility/and with nutrient-use efficiency; 16: Rosette-resistant variety; 17: Moderately-resistant variety (for short-duration variety) and highly- resistant variety (for medium- and long-duration varieties) to early and late leaf spot; 18: OPVs with host plant resistance to Striga hermonthica; 19: Soil fertility management for P and other nutrients (N, Ca) including chemical/organic fertilizers application; 20: Low P-tolerant/efficient variety. For semi-arid west and central Africa, in the case of the first technology, the normalized index for the benefit-cost ratio criterion is 0.4532; it’s 0.6027 and -0.4722 for the poverty and child malnutrition criteria, respectively. The weighted normalized index for the benefit-cost ratio, poverty and child malnutrition criteria become 0.1763, 0.1664, and -0.1582, respectively. The matrix for the positive-ideal solution is [0.1763; 0.1664; -0.1581] whereas the matrix for the negative -ideal solution is [0.0153; 0.0048; 0.0000]. The Euclidean distances to the positive- and negative-ideal solutions for the first technology are computed as follow: $S_{1}^{+}=\sqrt{\sum_{j=1}^{3}{\left(v_{1j}-v_{1j}^{+}\right)}^{2}};\;S_{1}^{+}={\left(0.1763-0.1763\right)}^{2}+{\left(0.1664-0.1664\right)}^{2}+{\left(-0.1581+(-0.1581)\right)}^{2}=0\;S_{1}^{-}=\sqrt{\sum_{j=1}^{3}{\left(v_{1j}-v_{1j}^{-}\right)}^{2}}$; $rS_{1}^{+}={\left(0.1763-0.0153\right)}^{2}+{\left(0.1664-0.0048\right)}^{2}+{\left(-0.1581+0.0000\right)}^{2}=0.2776$ The closeness index for the first technology is then computed as: $C_{i}^{+}=\frac{S_{i}^{-}}{S_{i}^{-}+S_{i}^{+}}=\frac{0.2776}{\left(0.2776+0\right)}=1$.(DOCX)

S10 Table Estimated closeness index and ranking of technologies in dry sub-humid west and central Africa. Tech: 1: Insect- (aphid, thrips, pod sucking bug, maruca) resistant lines and integrated pest management including biological control; 2: Medium- to late-maturing anthracnose-resistant cultivars; 3: Striga-resistant varieties and hybrids; 4: Drought-tolerant varieties and integrated crop management; 5: Low P-tolerant varieties and integrated crop management; 6: Drought-tolerant/resistant variety and short-duration (early- maturing) variety; 7: Striga-resistant varieties and integrated crop management; 8: Disease-resistant varieties and integrated crop management; 9: Pre and postharvest aflatoxin management practices including Good Agricultural Practices (GAP); 10: Drought-tolerant varieties and crop management and water conservation practices; 11: Head bugs- and grain mold-tolerant cultivars; 12: Disease-resistant varieties and integrated pest management and crop management practices; 13: Cultivars adapted to low soil fertility/and with nutrient-use efficiency;14: Increased plant population; 15: Use of inoculant and fertilizers especially Phosphorus; 16: Rosette-resistant variety; 17: Stem borer/midge-tolerant cultivars; 18: Moderately-resistant variety (for short-duration variety) and highly-resistant variety (for medium- and long-duration varieties) to early and late leaf spot; 19: Soil fertility management for P and other nutrients (N, Ca) including chemical/organic fertilizers application.
(DOCX)

S11 Table Estimated closeness index and ranking of technologies in semi-arid eastern Africa. Tech: 1: Intercropping compatible-varieties and integrated crop management options; 2: Early-maturing varieties and hybrids with tolerance to drought; 3: Fusarium wilt- and Cercospora leaf spot-resistant varieties; 4: Ascochyta blight-resistant varieties; 5: Photo- and thermo-insensitive varieties;6: Stem borer/midge-tolerant cultivars; 7: Striga-resistant varieties and hybrids; 8: Drought-tolerant/resistant variety and short-duration (early-maturing) variety 9: Fusarium wilt- and root rots-resistant varieties; 10: Validate and promote water management options; fertilizer regimes; 11: Cleisto varieties and maintenance breeding to reduce varietal degeneration due to out crossing; 12: Drought-tolerant varieties; 13: Integrated crop management options for soil fertility, water management, Striga, intercropping; 14: Pre and postharvest aflatoxin management practices including Good Agricultural Practices (GAP); 15: Varieties and hybrid parents with good establishment and respond well to drought especially terminal drought; 16: Drought-tolerant varieties; 17: Validate and promote water management options; fertilizer regimes; 18: Varieties tolerant to pod borers, pod fly, pod bugs and integrated pest management; 19: Varieties and hybrid parents with good establishment and that respond well to drought, especially terminal drought; 20: Early-maturing, drought-tolerant hybrids which can give stable yields under severe drought conditions; 21: Drought-tolerant varieties and integrated crop management; 22: Insect- (aphid, thrips, pod sucking bug, maruca) resistant lines and integrated crop management; 23: Rosette-resistant variety; 24: Genetically diverse dual-purpose hybrid parents/cultivars with high and stable yields with disease resistance (downy mildew and blast); 25: Early-maturing, drought-tolerant OPVs and hybrids which can give stable yields under severe drought conditions; 26: Moderately-resistant variety (for short-duration variety) and highly-resistant variety (for medium- and long-duration varieties) to early and late leaf spot; 27: Soil fertility management for P and other nutrients (N, Ca) including chemical/organic fertilizers application; 28: Downy mildew- and smut-resistant dual-purpose OPVs and hybrid parents; 29: Low P-tolerant varieties and integrated crop management; 30: Striga-resistant varieties and integrated crop management; 31: Waterlogging-tolerant varieties and management practices; 32: OPVs with host plant resistance to Striga hermonthica; 33: Drought-tolerant varieties; 34: Low P-tolerant/efficient variety; 35: Disease-resistant varieties and integrated crop management; 36: Weed management; 37: Ascochyta blight-resistant varieties; 38: Rust-resistant varieties.
(DOCX)

S12 Table Estimated closeness index and ranking of technologies in dry sub-humid eastern Africa. **Tech:** 1: Early-maturing varieties and hybrids with tolerance to drought; 2: Varieties and hybrids with resistance to Striga; 3: Medium- to late-maturing anthracnose-resistant cultivars; 4: Soil fertility management for P and other nutrients (N, Ca) including chemical/organic fertilizers application; 5: Integrated crop management options for soil fertility, water management, Striga, intercropping; 6: Photo- and thermo-insensitive varieties; 7: Cleisto varieties and maintenance breeding to reduce varietal degeneration due to outcrossing; 8: Varieties resistant to Fusarium wilt and Cercospora leaf spot; 9: Drought-tolerant varieties; 10: Weed control; 11: Varieties tolerant to warm temperatures; 12: Intercropping-compatible varieties and integrated crop management options; 13: Pre and postharvest aflatoxin management practices including Good Agricultural Practices (GAP); 14: Disease-resistant varieties and integrated pest management and crop management practices; 15: Drought-tolerant/resistant variety and short-duration (early- maturing) variety; 16: Drought-tolerant varieties and integrated crop management; 17: Lines resistant to insects (aphid, thrips, pod sucking bug, maruca) and integrated pest management including biological control; 18: Varieties tolerant to pod borers, pod fly, pod bugs and integrated pest management; 19: Alectra-resistant varieties and integrated crop management; 20: Low P-tolerant varieties and integrated crop management; 21: Rosette-resistant variety; 22: Moderately-resistant (for short-duration variety) and highly- resistant variety (for medium- and long-duration varieties) to early and late leaf spot; 23: Disease-resistant varieties and integrated crop management; 24: Integrated soil fertility management.
(DOCX)

S13 Table Estimated closeness index and ranking of technologies in semi-arid southern Africa. Tech: 1: Varieties resistant to Fusarium wilt and Cercospora leaf spot; 2: Intercropping-compatible varieties and integrated crop management options; 3: Cleisto varieties and maintenance breeding to reduce varietal degeneration due to outcrossing; 4: Drought-tolerant varieties; 5: Varieties tolerant to pod borers, pod fly, pod bugs and integrated pest management; 6: Photo- and thermo-insensitive varieties; 7: Low P-tolerant varieties and integrated crop management; 8: Disease-resistant varieties and integrated crop management; 9: Alectra-resistant varieties and integrated crop management; 10: Drought-tolerant varieties and integrated crop management; 11: Lines resistant to insects (aphid, thrips, pod sucking bug, maruca) and integrated pest management including biological control; 12: Integrated crop management options for soil fertility, water management, Striga, intercropping; 13: Varieties and hybrids with resistance to Striga; 14: Medium- to late-maturing anthracnose-resistant cultivars; 15: Early-maturing varieties and hybrids with tolerance to drought.
(DOCX)

S14 Table Estimated closeness index and ranking of technologies in dry sub-humid southern Africa. Tech: 1: Rosette-resistant variety; 2: Drought-tolerant/resistant variety and short-duration (early- maturing) variety; 3: Early-maturing varieties and hybrids with tolerance to drought; 4: Soil fertility management for P and other nutrients (N, Ca) including chemical/organic fertilizers application; 5: Drought-tolerant varieties and crop management and water conservation practices; 6: Pre and postharvest aflatoxin management practices including Good Agricultural Practices (GAP); 7: Disease-resistant varieties and integrated pest management and crop management practices; 8: Use of good quality seed, appropriate seeding rate and row spacing; 9: Medium- to late-maturing anthracnose-resistant cultivars; 10: Integrated crop management options for soil fertility, water management, Striga, intercropping; 11: Varieties and hybrids with resistance to Striga; 12: Matching varieties that fit the growing period; 13: Lines resistant to insects (aphid, thrip, pod sucking bug, maruca) and integrated crop management; 14: Drought-tolerant varieties and integrated crop management; 15: Moderately-resistant variety (for short-duration variety) and highly-resistant variety (for medium- and long-duration varieties) to early and late leaf spot; 16: Disease-resistant varieties and integrated crop management; 17: Low P-tolerant varieties and integrated crop management; 18: Striga and Alectra-resistant varieties and integrated crop management; 19: Low P-tolerant/efficient variety; 20: Breeding for resistance to parasitic weeds and integrated weed management; 21: Use of inoculant and fertilizers, especially Phosphorus; 22: Establish optimum planting window and awareness creation.
(DOCX)

S15 Table Estimated closeness index and ranking of technologies in semi-arid south Asia. Tech: 1: Varieties resistant to Fusarium wilt and root rots; 2: Drought-tolerant varieties; 3: Herbicide-tolerant varieties to control weeds; 4: Varieties resistant to Fusarium wilt and Cercospora leaf spot; 5: Genetically diverse dual-purpose hybrid parents/cultivars with high and stable yields with disease resistance (downy mildew and blast); 6: Breeding for early-maturing, drought-tolerant hybrids which can give stable yields under severe drought conditions; 7: Heat-tolerant varieties; 8 Pod borer-tolerant varieties and integrated pest management; 9: Soil fertility management for P and other nutrients (N, Ca) including chemical/organic fertilizers application; 10: Integrated crop management; 11: Sterility mosaic disease-resistant varieties; 12: Intercropping compatible-varieties and integrated crop management options; 13: Drought-tolerant varieties; 14: Integrated crop management; 15: Varieties tolerant to pod borers, pod fly, pod bugs and integrated pest management; 16: Genetic base diversification; 17: Pre and postharvest aflatoxin management practices including Good Agricultural Practices (GAP); 18: Varieties resistant to wilt and root rots and integrated pest management; 19: Cultivars tolerant to head bugs and grain mold; 20: Shoot fly-resistant cultivars; 21: Charcoal rot-resistant cultivars; 22: Early-maturing varieties and hybrids with tolerance to drought; 23: Drought-tolerant varieties; 24: Varieties resistant to diseases (foliar fungal, bud necrosis, soil borne); 25: Genetically diverse dual-purpose hybrid parents/cultivars with high and stable yields; 26: Integrated crop management practices; 27: Breeding for early-maturing, drought-tolerant OPVs and hybrids which can give stable yields under severe drought conditions; 28: Breeding for downy mildew- and smut-resistant dual-purpose OPVs and hybrid parents; 29: Herbicide-tolerant varieties to control weeds; 30: Heat-tolerant varieties; 31: Low P-tolerant/efficient variety.
(DOCX)

S16 Table Estimated closeness index and ranking of technologies in dry sub-humid South Asia. Tech: 1: Varieties resistant to Fusarium wilt and root rots; 2: Botrytis gray mold-resistant varieties; 3: Pod borer-tolerant varieties and integrated pest management; 4: Drought-tolerant varieties; 5 Herbicide-tolerant varieties to control weeds; 6: Drought-tolerant varieties; 7: Stemphylium blight-resistant varieties and integrated pest management; 8: Heat-tolerant varieties.
(DOCX)
