# Supplementary material for: Multi-dimensional impact assessment for priority setting of agricultural technologies: An application of TOPSIS for the drylands of sub-Saharan Africa and South Asia
Source: PLoS One. 2024 Nov 21;19(11):e0314007. doi: 10.1371/journal.pone.0314007 (PMC11581267; doi:10.1371/journal.pone.0314007)
Supplement: S1 Table — Tech: 1: Drought-tolerant varieties and integrated crop management; 2: Insect- (aphid, thrips, pod sucking bug, maruca) resistant lines and integrated pest management including biological control; 3: Disease-resistant varieties and integrated crop management; 4: Striga-resistant varieties and integrated crop management; 5: Low P-tolerant varieties and integrated crop management; 6: Drought-tolerant/resistant variety and short-duration (early-maturing) variety; 7: Moderately-resistant variety (for short-duration variety) and highly- resistant variety (for medium- and long-duration varieties) to early and late leaf spot; 8: Rosette-resistant variety; 9: Low P-tolerant/efficient variety; 10: Pre and postharvest aflatoxin management practices including Good Agricultural Practices (GAP); 11: Soil fertility management for P and other nutrients (N, Ca) including chemical/organic fertilizers application; 12: Early-maturing, drought-tolerant hybrids which can give stable yields under severe drought conditions; 13: Genetically diverse dual-purpose hybrid parents/cultivars with high and stable yields with disease resistance (downy mildew and blast); 14: Biological control of millet head miner and resistant hybrid parents; 15: Integrated soil fertility management and identifying genotypes for low P tolerance; 16: OPVs with host plant resistance to Striga hermonthica; 17: Early-maturing varieties and hybrids with tolerance to drought; 18: Striga-resistant varieties and hybrids; 19: Cultivars adapted to low soil fertility/and with nutrient-use efficiency; 20: Stem borer/midge-tolerant cultivars. (DOCX) [file pone.0314007.s001.docx]

S1 Table: Research, dissemination and adoption parameters for improved technologies - semi-arid West and Central Africa

| Crop | Tech |  | Farm changes | | | | | | |  | Macro-level parameters | | | | |  | Research and dissemination costs | | |
| --- | --- | --- | --- | --- | --- | --- | --- | --- | --- | --- | --- | --- | --- | --- | --- | --- | --- | --- | --- |
|  |  |  | Max adoption (%) | Adoption years | Supply elas. | Demand elas. | Yield change (%) | Cost change (%) | Probability of success |  | Price (US$/ton) | Quantity (mil. tons) | Area harvested (mil. ha) | Poverty headcount (mil. people) | Ag. GDP (bil. US$) |  | Res. Years | Res. Costs (‘000 US$/year) | Diss. Cost (US$/ha) |
| Cowpea | 1 |  | 40 | 10 | 1.0 | -0.5 | 70 | 10 | 80 |  | 500 | 2.4 | 5.0 | 50 | 38 |  | 10 | 300 | 50 |
| Cowpea | 2 |  | 40 | 10 | 1.0 | -0.5 | 75 | 20 | 80 |  | 500 | 2.4 | 5.0 | 50 | 38 |  | 10 | 350 | 50 |
| Cowpea | 3 |  | 40 | 10 | 1.0 | -0.5 | 40 | 20 | 80 |  | 500 | 2.4 | 5.0 | 50 | 38 |  | 10 | 350 | 50 |
| Cowpea | 4 |  | 40 | 10 | 1.0 | -0.5 | 40 | 20 | 80 |  | 500 | 2.4 | 5.0 | 50 | 38 |  | 10 | 200 | 50 |
| Cowpea | 5 |  | 35 | 10 | 1.0 | -0.5 | 60 | 20 | 80 |  | 500 | 2.4 | 5.0 | 50 | 38 |  | 10 | 320 | 50 |
| Groundnuts | 6 |  | 40 | 10 | 1.0 | -0.5 | 45 | 30 | 80 |  | 811 | 1.9 | 1.4 | 50 | 38 |  | 10 | 390 | 50 |
| Groundnuts | 7 |  | 60 | 10 | 1.0 | -0.5 | 30 | 30 | 80 |  | 811 | 1.9 | 1.4 | 50 | 38 |  | 10 | 250 | 50 |
| Groundnuts | 8 |  | 40 | 10 | 1.0 | -0.5 | 30 | 30 | 95 |  | 811 | 1.9 | 1.4 | 50 | 38 |  | 10 | 250 | 50 |
| Groundnuts | 9 |  | 50 | 10 | 1.0 | -0.5 | 30 | 30 | 30 |  | 811 | 1.9 | 1.4 | 50 | 38 |  | 10 | 80 | 50 |
| Groundnuts | 10 |  | 40 | 7 | 1.0 | -0.5 | 20 | 5 | 70 |  | 811 | 1.9 | 1.4 | 50 | 38 |  | 5 | 150 | 75 |
| Groundnuts | 11 |  | 40 | 7 | 1.0 | -0.5 | 30 | 30 | 70 |  | 811 | 1.9 | 1.4 | 50 | 38 |  | 5 | 120 | 75 |
| Pearl millet | 12 |  | 40 | 10 | 1.0 | -0.5 | 60 | 15 | 60 |  | 303 | 8.3 | 7.6 | 50 | 38 |  | 10 | 500 | 50 |
| Pearl millet | 13 |  | 40 | 10 | 1.0 | -0.5 | 50 | 15 | 80 |  | 303 | 8.3 | 7.6 | 50 | 38 |  | 10 | 250 | 50 |
| Pearl millet | 14 |  | 40 | 10 | 1.0 | -0.5 | 40 | 15 | 70 |  | 303 | 8.3 | 7.6 | 50 | 38 |  | 10 | 400 | 75 |
| Pearl millet | 15 |  | 40 | 10 | 1.0 | -0.5 | 30 | 15 | 60 |  | 303 | 8.3 | 7.6 | 50 | 38 |  | 10 | 250 | 75 |
| Pearl millet | 16 |  | 40 | 10 | 1.0 | -0.5 | 20 | 15 | 60 |  | 303 | 8.3 | 7.6 | 50 | 38 |  | 10 | 250 | 50 |
| Sorghum | 17 |  | 60 | 10 | 1.0 | -0.4 | 85 | 10 | 90 |  | 152 | 7.3 | 6.6 | 50 | 38 |  | 5 | 317 | 50 |
| Sorghum | 18 |  | 60 | 10 | 1.0 | -0.4 | 50 | 10 | 70 |  | 152 | 7.3 | 6.6 | 50 | 38 |  | 7 | 267 | 50 |
| Sorghum | 19 |  | 60 | 15 | 1.0 | -0.4 | 30 | 10 | 70 |  | 152 | 7.3 | 6.6 | 50 | 38 |  | 7 | 267 | 50 |
| Sorghum | 20 |  | 60 | 10 | 1.0 | -0.4 | 30 | 10 | 80 |  | 152 | 7.3 | 6.6 | 50 | 38 |  | 10 | 217 | 50 |

Tech:

1: Drought-tolerant varieties and integrated crop management; 2: Insect- (aphid, thrips, pod sucking bug, maruca) resistant lines and integrated pest management including biological control; 3: Disease-resistant varieties and integrated crop management; 4: Striga-resistant varieties and integrated crop management; 5: Low P-tolerant varieties and integrated crop management; 6: Drought-tolerant/resistant variety and short-duration (early-maturing) variety; 7: Moderately-resistant variety (for short-duration variety) and highly- resistant variety (for medium- and long-duration varieties) to early and late leaf spot; 8: Rosette-resistant variety; 9: Low P-tolerant/efficient variety; 10: Pre and postharvest aflatoxin management practices including Good Agricultural Practices (GAP); 11: Soil fertility management for P and other nutrients (N, Ca) including chemical/organic fertilizers application; 12: Early-maturing, drought-tolerant hybrids which can give stable yields under severe drought conditions; 13: Genetically diverse dual-purpose hybrid parents/cultivars with high and stable yields with disease resistance (downy mildew and blast); 14: Biological control of millet head miner and resistant hybrid parents; 15: Integrated soil fertility management and identifying genotypes for low P tolerance; 16: OPVs with host plant resistance to Striga hermonthica; 17: Early-maturing varieties and hybrids with tolerance to drought; 18: Striga-resistant varieties and hybrids; 19: Cultivars adapted to low soil fertility/and with nutrient-use efficiency; 20: Stem borer/midge-tolerant cultivars
