# Supplementary material for: Multi-dimensional impact assessment for priority setting of agricultural technologies: An application of TOPSIS for the drylands of sub-Saharan Africa and South Asia
Source: PLoS One. 2024 Nov 21;19(11):e0314007. doi: 10.1371/journal.pone.0314007 (PMC11581267; doi:10.1371/journal.pone.0314007)
Supplement: S2 Table — Tech: 1: Striga-resistant varieties and integrated crop management; 2: Disease-resistant varieties and integrated crop management; 3: Drought-tolerant varieties and integrated crop management; 4: Insect- (aphid, thrips, pod sucking bug, maruca) resistant lines and integrated pest management including biological control; 5: Low P-tolerant varieties and integrated crop management; 6: Drought-tolerant/resistant variety and short-duration (early- maturing) variety; 7: Increased plant population; 8: Moderately-resistant variety (for short-duration variety) and highly-resistant variety (for medium- and long-duration varieties) to early and late leaf spot; 9: Pre and postharvest aflatoxin management practices including Good Agricultural Practices (GAP); 10: Rosette-resistant variety; 11: Soil fertility management for P and other nutrients (N, Ca) including chemical/organic fertilizers application; 12: Head bugs- and grain mold-tolerant cultivars; 13: Medium- to late-maturing anthracnose-resistant cultivars; 14: Striga-resistant varieties and hybrids; 15: Cultivars adapted to low soil fertility/and with nutrient-use efficiency; 16: Stem borer/midge-tolerant cultivars; 17: Drought-tolerant varieties and crop management and water conservation practices; 18: Disease-resistant varieties and integrated pest management and crop management practices; 19: Use of inoculant and fertilizers especially Phosphorus. (DOCX) [file pone.0314007.s002.docx]

S2 Table: Research, dissemination and adoption parameters for improved technologies – dry sub-humid West and Central Africa

| Crop | Tech |  | Farm changes | | | | | | |  | Macro-level parameters | | | | |  | Research and dissemination costs | | |
| --- | --- | --- | --- | --- | --- | --- | --- | --- | --- | --- | --- | --- | --- | --- | --- | --- | --- | --- | --- |
|  |  |  | Max adoption (%) | Adoption years | Supply elas. | Demand elas. | Yield change (%) | Cost change (%) | Probability of success |  | Price (US$/ton) | Quantity (mil. tons) | Area harvested (mil. ha) | Poverty headcount (mil. people) | Ag. GDP (bil. US$) |  | Res. Years | Res. Costs (‘000 US$/year) | Diss. Cost (US$/ha) |
| Cowpea | 1 |  | 40 | 10 | 1.0 | -0.5 | 40 | 20 | 80 |  | 500 | 1.1 | 1.4 | 22 | 33 |  | 10 | 200 | 50 |
| Cowpea | 2 |  | 40 | 10 | 1.0 | -0.5 | 40 | 20 | 80 |  | 500 | 1.1 | 1.4 | 22 | 33 |  | 10 | 350 | 50 |
| Cowpea | 3 |  | 40 | 10 | 1.0 | -0.5 | 50 | 10 | 80 |  | 500 | 1.1 | 1.4 | 22 | 33 |  | 10 | 300 | 50 |
| Cowpea | 4 |  | 40 | 10 | 1.0 | -0.5 | 60 | 20 | 80 |  | 500 | 1.1 | 1.4 | 22 | 33 |  | 10 | 350 | 50 |
| Cowpea | 5 |  | 35 | 10 | 1.0 | -0.5 | 50 | 20 | 80 |  | 500 | 1.1 | 1.4 | 22 | 33 |  | 10 | 320 | 50 |
| Groundnuts | 6 |  | 40 | 10 | 1.0 | -0.5 | 40 | 30 | 80 |  | 811 | 1.9 | 1.4 | 22 | 33 |  | 10 | 390 | 50 |
| Groundnuts | 7 |  | 30 | 7 | 1.0 | -0.4 | 40 | 40 | 80 |  | 811 | 1.9 | 1.4 | 22 | 33 |  | 5 | 200 | 75 |
| Groundnuts | 8 |  | 40 | 10 | 1.0 | -0.4 | 30 | 30 | 80 |  | 811 | 1.9 | 1.4 | 22 | 33 |  | 10 | 250 | 50 |
| Groundnuts | 9 |  | 40 | 7 | 1.0 | -0.5 | 20 | 5 | 70 |  | 811 | 1.9 | 1.4 | 22 | 33 |  | 5 | 150 | 75 |
| Groundnuts | 10 |  | 40 | 10 | 1.0 | -0.4 | 30 | 30 | 95 |  | 811 | 1.9 | 1.4 | 22 | 33 |  | 10 | 250 | 50 |
| Groundnuts | 11 |  | 40 | 7 | 1.0 | -0.5 | 30 | 30 | 70 |  | 811 | 1.9 | 1.4 | 22 | 33 |  | 5 | 120 | 75 |
| Sorghum | 12 |  | 60 | 10 | 1.0 | -0.4 | 30 | 10 | 70 |  | 152 | 4.1 | 3.4 | 22 | 33 |  | 7 | 293 | 50 |
| Sorghum | 13 |  | 60 | 10 | 1.0 | -0.4 | 40 | 10 | 90 |  | 152 | 4.1 | 3.4 | 22 | 33 |  | 5 | 243 | 50 |
| Sorghum | 14 |  | 60 | 10 | 1.0 | -0.4 | 40 | 10 | 70 |  | 152 | 4.1 | 3.4 | 22 | 33 |  | 5 | 293 | 50 |
| Sorghum | 15 |  | 60 | 15 | 1.0 | -0.4 | 25 | 10 | 90 |  | 152 | 4.1 | 3.4 | 22 | 33 |  | 7 | 393 | 50 |
| Sorghum | 16 |  | 60 | 10 | 1.0 | -0.4 | 25 | 10 | 80 |  | 152 | 4.1 | 3.4 | 22 | 33 |  | 10 | 343 | 50 |
| Soybean | 17 |  | 55 | 10 | 1.0 | -0.6 | 50 | 10 | 80 |  | 347 | 0.3 | 0.3 | 21 | 33 |  | 5 | 400 | 50 |
| Soybean | 18 |  | 55 | 10 | 1.0 | -0.6 | 40 | 10 | 80 |  | 347 | 0.3 | 0.3 | 21 | 33 |  | 5 | 200 | 50 |
| Soybean | 19 |  | 35 | 10 | 1.0 | -0.6 | 50 | 25 | 100 |  | 347 | 0.3 | 0.3 | 21 | 33 |  | 3 | 400 | 75 |

Tech:

1: Striga-resistant varieties and integrated crop management; 2: Disease-resistant varieties and integrated crop management; 3: Drought-tolerant varieties and integrated crop management; 4: Insect- (aphid, thrips, pod sucking bug, maruca) resistant lines and integrated pest management including biological control; 5: Low P-tolerant varieties and integrated crop management; 6: Drought-tolerant/resistant variety and short-duration (early- maturing) variety; 7: Increased plant population; 8: Moderately-resistant variety (for short-duration variety) and highly-resistant variety (for medium- and long-duration varieties) to early and late leaf spot; 9: Pre and postharvest aflatoxin management practices including Good Agricultural Practices (GAP); 10: Rosette-resistant variety; 11: Soil fertility management for P and other nutrients (N, Ca) including chemical/organic fertilizers application; 12: Head bugs- and grain mold-tolerant cultivars; 13: Medium- to late-maturing anthracnose-resistant cultivars; 14: Striga-resistant varieties and hybrids; 15: Cultivars adapted to low soil fertility/and with nutrient-use efficiency; 16: Stem borer/midge-tolerant cultivars; 17: Drought-tolerant varieties and crop management and water conservation practices; 18: Disease-resistant varieties and integrated pest management and crop management practices; 19: Use of inoculant and fertilizers especially Phosphorus
