# Supplementary material for: Multi-dimensional impact assessment for priority setting of agricultural technologies: An application of TOPSIS for the drylands of sub-Saharan Africa and South Asia
Source: PLoS One. 2024 Nov 21;19(11):e0314007. doi: 10.1371/journal.pone.0314007 (PMC11581267; doi:10.1371/journal.pone.0314007)
Supplement: S3 Table — * Fgr millet = Finger millet; Tech: 1: Ascochyta blight-resistant varieties; 2: Drought-tolerant varieties; 3: Fusarium wilt- and root rots-resistant varieties; 4; Waterlogging-tolerant varieties and management practices; 5: Striga-resistant varieties and integrated crop management; 6: Disease-resistant varieties and integrated crop management; 7: Drought-tolerant varieties and integrated crop management; 8: Insect- (aphid, thrips, pod sucking bug, maruca) resistant lines and integrated crop management; 9: Low P-tolerant varieties and integrated crop management; 10: Downy mildew- and smut-resistant dual-purpose OPVs and hybrid parents; 11: OPVs with host plant resistance to Striga hermonthica; 12: Early-maturing, drought-tolerant OPVs and hybrids which can give stable yields under severe drought conditions; 13: Varieties and hybrid parents with good establishment and that respond well to drought, especially terminal drought; 14: Validate and promote water management options; fertilizer regimes; 15: Drought-tolerant/resistant variety and short-duration (early-maturing) variety; 16: Low P-tolerant/efficient variety; 17: Moderately-resistant variety (for short-duration variety) and highly-resistant variety (for medium- and long-duration varieties) to early and late leaf spot; 18: Pre and postharvest aflatoxin management practices including Good Agricultural Practices (GAP); 19: Rosette-resistant variety; 20: Soil fertility management for P and other nutrients (N, Ca) including chemical/organic fertilizers application; 21: Ascochyta blight-resistant varieties; 22: Drought-tolerant varieties; 23: Rust-resistant varieties; 24: Weed management; 25: Genetically diverse dual-purpose hybrid parents/cultivars with high and stable yields with disease resistance (downy mildew and blast); 26: Early-maturing, drought-tolerant hybrids which can give stable yields under severe drought conditions; 27: Varieties and hybrid parents with good establishment and respond well to drought es [file pone.0314007.s003.docx]

S3 Table: Research, dissemination and adoption parameters for improved technologies – semi-arid eastern Africa

| Crop | Tech |  | Farm changes | | | | | | |  | Macro-level parameters | | | | |  | Research and dissemination costs | | |
| --- | --- | --- | --- | --- | --- | --- | --- | --- | --- | --- | --- | --- | --- | --- | --- | --- | --- | --- | --- |
|  |  |  | Max adoption (%) | Adoption years | Supply elas. | Demand elas. | Yield change (%) | Cost change (%) | Probability of success |  | Price (US$/ton) | Quantity (mil. tons) | Area harvested (mil. ha) | Poverty headcount (mil. people) | Ag. GDP (bil. US$) |  | Res. Years | Res. Costs (‘000 US$/year) | Diss. Cost (US$/ha) |
| Chickpea | 1 |  | 40 | 10 | 1.0 | -0.5 | 50 | 20 | 80 |  | 500 | 0.1 | 0.2 | 20 | 20 |  | 11 | 200 | 50 |
| Chickpea | 2 |  | 60 | 10 | 1.0 | -0.5 | 35 | 20 | 90 |  | 577 | 0.1 | 0.1 | 18 | 56 |  | 4 | 200 | 50 |
| Chickpea | 3 |  | 60 | 10 | 1.0 | -0.5 | 50 | 20 | 70 |  | 577 | 0.1 | 0.1 | 18 | 56 |  | 4 | 200 | 50 |
| Chickpea | 4 |  | 40 | 10 | 1.0 | -0.5 | 35 | 20 | 80 |  | 577 | 0.1 | 0.1 | 18 | 56 |  | 5 | 200 | 50 |
| Cowpea | 5 |  | 30 | 10 | 1.0 | -0.5 | 40 | 20 | 80 |  | 500 | 0.1 | 0.2 | 20 | 20 |  | 11 | 250 | 50 |
| Cowpea | 6 |  | 30 | 10 | 1.0 | -0.5 | 60 | 10 | 80 |  | 500 | 0.1 | 0.2 | 20 | 20 |  | 10 | 200 | 50 |
| Cowpea | 7 |  | 30 | 10 | 1.0 | -0.5 | 70 | 20 | 80 |  | 500 | 0.1 | 0.2 | 20 | 20 |  | 11 | 250 | 50 |
| Cowpea | 8 |  | 30 | 10 | 1.0 | -0.5 | 40 | 20 | 80 |  | 500 | 0.1 | 0.2 | 20 | 20 |  | 11 | 120 | 50 |
| Cowpea | 9 |  | 30 | 10 | 1.0 | -0.5 | 40 | 20 | 80 |  | 600 | 0.2 | 0.1 | 8 | 7 |  | 5 | 400 | 50 |
| Fgr millet* | 10 |  | 40 | 10 | 1.0 | -0.5 | 30 | 30 | 70 |  | 811 | 0.2 | 0.2 | 20 | 33 |  | 5 | 120 | 75 |
| Fgr millet | 11 |  | 40 | 10 | 1.0 | -0.5 | 60 | 10 | 70 |  | 152 | 2.8 | 3.7 | 20 | 20 |  | 5 | 178 | 50 |
| Fgr millet | 12 |  | 40 | 10 | 1.0 | -0.5 | 50 | 20 | 70 |  | 303 | 0.2 | 0.1 | 10 | 16 |  | 10 | 300 | 50 |
| Fgr millet | 13 |  | 40 | 10 | 1.0 | -0.5 | 50 | 20 | 80 |  | 303 | 0.2 | 0.1 | 10 | 16 |  | 10 | 200 | 50 |
| Fgr millet | 14 |  | 40 | 10 | 1.0 | -0.5 | 30 | 30 | 90 |  | 811 | 0.5 | 0.7 | 20 | 20 |  | 10 | 250 | 50 |
| Groundnuts | 15 |  | 40 | 10 | 1.0 | -0.5 | 30 | 30 | 40 |  | 811 | 0.5 | 0.7 | 20 | 20 |  | 10 | 80 | 50 |
| Groundnuts | 16 |  | 40 | 10 | 1.0 | -0.5 | 30 | 30 | 95 |  | 811 | 0.5 | 0.7 | 20 | 20 |  | 8 | 250 | 50 |
| Groundnuts | 17 |  | 40 | 10 | 1.0 | -0.5 | 10 | 30 | 20 |  | 811 | 0.5 | 0.7 | 20 | 20 |  | 10 | 50 | 75 |
| Groundnuts | 18 |  | 40 | 7 | 1.0 | -0.5 | 60 | 15 | 50 |  | 303 | 0.6 | 1.3 | 18 | 20 |  | 10 | 200 | 50 |
| Groundnuts | 19 |  | 40 | 10 | 1.0 | -0.5 | 20 | 5 | 70 |  | 811 | 0.2 | 0.2 | 20 | 33 |  | 5 | 150 | 75 |
| Groundnuts | 20 |  | 40 | 7 | 1.0 | -0.5 | 30 | 10 | 70 |  | 545 | 0.0 | 0.0 | 20 | 19 |  | 5 | 257 | 50 |
| Lentil | 21 |  | 60 | 10 | 1.0 | -0.5 | 20 | 10 | 50 |  | 545 | 0.0 | 0.0 | 20 | 19 |  | 6 | 257 | 50 |
| Lentil | 22 |  | 50 | 10 | 1.0 | -0.5 | 40 | 20 | 50 |  | 600 | 0.2 | 0.1 | 8 | 7 |  | 5 | 400 | 50 |
| Lentil | 23 |  | 60 | 10 | 1.0 | -0.5 | 15 | 10 | 80 |  | 545 | 0.0 | 0.0 | 20 | 19 |  | 4 | 257 | 50 |
| Lentil | 24 |  | 60 | 10 | 1.0 | -0.5 | 15 | 10 | 100 |  | 545 | 0.0 | 0.0 | 20 | 19 |  | 4 | 257 | 50 |
| Pearl millet | 25 |  | 40 | 10 | 1.0 | -0.5 | 50 | 20 | 50 |  | 303 | 0.2 | 0.1 | 10 | 16 |  | 10 | 200 | 50 |
| Pearl millet | 26 |  | 40 | 10 | 1.0 | -0.5 | 60 | 15 | 70 |  | 303 | 0.6 | 1.3 | 18 | 20 |  | 10 | 300 | 50 |
| Pearl millet | 27 |  | 40 | 10 | 1.0 | -0.5 | 60 | 15 | 80 |  | 303 | 0.6 | 1.3 | 18 | 20 |  | 10 | 200 | 50 |
| Pearl millet | 28 |  | 30 | 10 | 1.0 | -0.5 | 50 | 30 | 70 |  | 494 | 0.1 | 0.2 | 18 | 20 |  | 5 | 86 | 50 |
| Pigeon pea | 29 |  | 50 | 10 | 1.0 | -0.5 | 50 | 30 | 90 |  | 494 | 0.1 | 0.2 | 18 | 20 |  | 5 | 106 | 75 |
| Pigeon pea | 30 |  | 50 | 10 | 1.0 | -0.5 | 50 | 30 | 90 |  | 494 | 0.1 | 0.2 | 18 | 20 |  | 5 | 176 | 50 |
| Pigeon pea | 31 |  | 50 | 10 | 1.0 | -0.5 | 50 | 30 | 50 |  | 494 | 0.1 | 0.2 | 18 | 20 |  | 6 | 106 | 50 |
| Pigeon pea | 32 |  | 50 | 10 | 1.0 | -0.5 | 60 | 30 | 90 |  | 494 | 0.1 | 0.2 | 18 | 20 |  | 5 | 126 | 50 |
| Pigeon pea | 33 |  | 50 | 10 | 1.0 | -0.5 | 15 | 10 | 80 |  | 545 | 0.0 | 0.0 | 20 | 19 |  | 3 | 257 | 75 |
| Pigeon pea | 34 |  | 50 | 10 | 1.0 | -0.5 | 40 | 10 | 70 |  | 347 | 0.0 | 0.0 | 18 | 20 |  | 4 | 300 | 50 |
| Sorghum | 35 |  | 40 | 10 | 1.0 | -0.4 | 85 | 10 | 90 |  | 152 | 2.8 | 3.7 | 20 | 20 |  | 5 | 250 | 50 |
| Sorghum | 36 |  | 40 | 10 | 1.0 | -0.4 | 60 | 10 | 70 |  | 152 | 2.8 | 3.7 | 20 | 20 |  | 5 | 228 | 50 |
| Sorghum | 37 |  | 60 | 10 | 1.0 | -0.4 | 60 | 10 | 70 |  | 152 | 2.8 | 3.7 | 20 | 20 |  | 3 | 178 | 75 |
| Sorghum | 38 |  | 60 | 10 | 1.0 | -0.4 | 50 | 30 | 80 |  | 494 | 0.1 | 0.2 | 18 | 20 |  | 5 | 126 | 50 |
|  |  |  |  |  |  |  |  |  |  |  |  |  |  |  |  |  |  |  |  |

* Fgr millet = Finger millet

Tech:

1: Ascochyta blight-resistant varieties; 2: Drought-tolerant varieties; 3: Fusarium wilt- and root rots-resistant varieties; 4; Waterlogging-tolerant varieties and management practices; 5: Striga-resistant varieties and integrated crop management; 6: Disease-resistant varieties and integrated crop management; 7: Drought-tolerant varieties and integrated crop management; 8: Insect- (aphid, thrips, pod sucking bug, maruca) resistant lines and integrated crop management; 9: Low P-tolerant varieties and integrated crop management; 10: Downy mildew- and smut-resistant dual-purpose OPVs and hybrid parents; 11: OPVs with host plant resistance to Striga hermonthica; 12: Early-maturing, drought-tolerant OPVs and hybrids which can give stable yields under severe drought conditions; 13: Varieties and hybrid parents with good establishment and that respond well to drought, especially terminal drought; 14: Validate and promote water management options; fertilizer regimes; 15: Drought-tolerant/resistant variety and short-duration (early-maturing) variety; 16: Low P-tolerant/efficient variety; 17: Moderately-resistant variety (for short-duration variety) and highly-resistant variety (for medium- and long-duration varieties) to early and late leaf spot; 18: Pre and postharvest aflatoxin management practices including Good Agricultural Practices (GAP); 19: Rosette-resistant variety; 20: Soil fertility management for P and other nutrients (N, Ca) including chemical/organic fertilizers application; 21: Ascochyta blight-resistant varieties; 22: Drought-tolerant varieties; 23: Rust-resistant varieties; 24: Weed management; 25: Genetically diverse dual-purpose hybrid parents/cultivars with high and stable yields with disease resistance (downy mildew and blast); 26: Early-maturing, drought-tolerant hybrids which can give stable yields under severe drought conditions; 27: Varieties and hybrid parents with good establishment and respond well to drought especially terminal drought; 28: Validate and promote water management options; fertilizer regimes; 29: Cleisto varieties and maintenance breeding to reduce varietal degeneration due to out crossing; 30: Drought-tolerant varieties; 31: Fusarium wilt- and Cercospora leaf spot-resistant varieties; 32: Intercropping compatible-varieties and integrated crop management options; 33: Varieties tolerant to pod borers, pod fly, pod bugs and integrated pest management; 34: Photo- and thermo-insensitive varieties; 35: early-maturing varieties and hybrids with tolerance to drought; 36: Integrated crop management options for soil fertility, water management, Striga, intercropping; 37: Stem borer/midge-tolerant cultivars; 38: Striga-resistant varieties and hybrids
