# Supplementary material for: Multi-dimensional impact assessment for priority setting of agricultural technologies: An application of TOPSIS for the drylands of sub-Saharan Africa and South Asia
Source: PLoS One. 2024 Nov 21;19(11):e0314007. doi: 10.1371/journal.pone.0314007 (PMC11581267; doi:10.1371/journal.pone.0314007)
Supplement: S4 Table — Tech: 1: Alectra-resistant varieties and integrated crop management; 2: Disease-resistant varieties and integrated crop management; 3: Drought-tolerant varieties and integrated crop management; 4: Lines resistant to insects (aphid, thrips, pod sucking bug, maruca) and integrated pest management including biological control; 5: Low P-tolerant varieties and integrated crop management; 6: Drought-tolerant/resistant variety and short-duration (early- maturing) variety; 7: Moderately-resistant (for short-duration variety) and highly- resistant variety (for medium- and long-duration varieties) to early and late leaf spot; 8: Pre and postharvest aflatoxin management practices including Good Agricultural Practices (GAP); 9: Rosette-resistant variety; 10: Soil fertility management for P and other nutrients (N, Ca) including chemical/organic fertilizers application; 11: Varieties resistant to Fusarium wilt and Cercospora leaf spot; 12: Cleisto varieties and maintenance breeding to reduce varietal degeneration due to outcrossing; 13: Drought-tolerant varieties; 14: Weed control; 15: Intercropping-compatible varieties and integrated crop management options; 16: Varieties tolerant to pod borers, pod fly, pod bugs and integrated pest management; 17: Photo- and thermo-insensitive varieties; 18: Varieties tolerant to warm temperatures; 19: Early-maturing varieties and hybrids with tolerance to drought; 20: Integrated crop management options for soil fertility, water management, Striga, intercropping; 21: Medium- to late-maturing anthracnose-resistant cultivars; 22: Varieties and hybrids with resistance to Striga; 23: Integrated soil fertility management; 24: Disease-resistant varieties and integrated pest management and crop management practices. (DOCX) [file pone.0314007.s004.docx]

S4 Table: research dissemination and adoption parameters for improved technologies – dry sub-humid eastern Africa

| Crop | Tech |  | Farm changes | | | | | | |  | Macro-level parameters | | | | |  | Research and dissemination costs | | |
| --- | --- | --- | --- | --- | --- | --- | --- | --- | --- | --- | --- | --- | --- | --- | --- | --- | --- | --- | --- |
|  |  |  | Max adoption (%) | Adoption years | Supply elas. | Demand elas. | Yield change (%) | Cost change (%) | Probability of success |  | Price (US$/ton) | Quantity (mil. tons) | Area harvested (mil. ha) | Poverty headcount (mil. people) | Ag. GDP (bil. US$) |  | Res. Years | Res. Costs (‘000 US$/year) | Diss. Cost (US$/ha) |
| Cowpea | 1 |  | 40 | 10 | 1.0 | -0.5 | 40 | 20 | 80 |  | 500 | 0.1 | 0.2 | 31 | 14 |  | 10 | 120 | 50 |
| Cowpea | 2 |  | 40 | 10 | 1.0 | -0.5 | 40 | 20 | 80 |  | 500 | 0.1 | 0.2 | 31 | 14 |  | 10 | 250 | 50 |
| Cowpea | 3 |  | 40 | 10 | 1.0 | -0.5 | 60 | 10 | 80 |  | 500 | 0.1 | 0.2 | 31 | 14 |  | 10 | 200 | 50 |
| Cowpea | 4 |  | 40 | 10 | 1.0 | -0.5 | 70 | 20 | 80 |  | 500 | 0.1 | 0.2 | 31 | 14 |  | 10 | 250 | 50 |
| Cowpea | 5 |  | 30 | 10 | 1.0 | -0.5 | 50 | 20 | 80 |  | 500 | 0.1 | 0.2 | 31 | 14 |  | 10 | 200 | 50 |
| Groundnuts | 6 |  | 40 | 10 | 1.0 | -0.4 | 40 | 30 | 80 |  | 811 | 0.3 | 0.4 | 31 | 14 |  | 10 | 250 | 50 |
| Groundnuts | 7 |  | 40 | 10 | 1.0 | -0.4 | 30 | 30 | 90 |  | 811 | 0.3 | 0.4 | 31 | 14 |  | 10 | 250 | 50 |
| Groundnuts | 8 |  | 40 | 7 | 1.0 | -0.5 | 20 | 5 | 70 |  | 811 | 0.3 | 0.3 | 31 | 14 |  | 5 | 150 | 75 |
| Groundnuts | 9 |  | 40 | 10 | 1.0 | -0.4 | 30 | 30 | 95 |  | 811 | 0.3 | 0.4 | 31 | 14 |  | 8 | 250 | 50 |
| Groundnuts | 10 |  | 40 | 7 | 1.0 | -0.5 | 50 | 30 | 70 |  | 811 | 0.3 | 0.3 | 31 | 14 |  | 5 | 120 | 75 |
| Pigeon pea | 11 |  | 50 | 10 | 1.0 | -0.5 | 50 | 30 | 70 |  | 494 | 0.1 | 0.2 | 25 | 11 |  | 5 | 69 | 50 |
| Pigeon pea | 12 |  | 50 | 10 | 1.0 | -0.5 | 50 | 30 | 80 |  | 494 | 0.1 | 0.2 | 25 | 11 |  | 5 | 119 | 50 |
| Pigeon pea | 13 |  | 50 | 10 | 1.0 | -0.5 | 50 | 30 | 70 |  | 494 | 0.1 | 0.2 | 25 | 11 |  | 5 | 89 | 50 |
| Pigeon pea | 14 |  | 50 | 10 | 1.0 | -0.5 | 50 | 30 | 80 |  | 494 | 0.1 | 0.2 | 25 | 11 |  | 4 | 39 | 75 |
| Pigeon pea | 15 |  | 50 | 10 | 1.0 | -0.5 | 50 | 30 | 90 |  | 494 | 0.1 | 0.2 | 25 | 11 |  | 5 | 125 | 75 |
| Pigeon pea | 16 |  | 50 | 10 | 1.0 | -0.5 | 50 | 30 | 50 |  | 494 | 0.1 | 0.2 | 25 | 11 |  | 6 | 105 | 50 |
| Pigeon pea | 17 |  | 50 | 10 | 1.0 | -0.5 | 50 | 30 | 90 |  | 494 | 0.1 | 0.2 | 25 | 11 |  | 5 | 169 | 50 |
| Pigeon pea | 18 |  | 50 | 10 | 1.0 | -0.5 | 50 | 30 | 60 |  | 494 | 0.1 | 0.2 | 25 | 11 |  | 5 | 49 | 50 |
| Sorghum | 19 |  | 60 | 10 | 1.0 | -0.4 | 80 | 10 | 90 |  | 152 | 1.9 | 1.5 | 31 | 14 |  | 5 | 328 | 50 |
| Sorghum | 20 |  | 40 | 10 | 1.0 | -0.4 | 60 | 10 | 75 |  | 152 | 1.9 | 1.5 | 31 | 14 |  | 3 | 178 | 75 |
| Sorghum | 21 |  | 60 | 10 | 1.0 | -0.4 | 60 | 10 | 75 |  | 152 | 1.9 | 1.5 | 31 | 14 |  | 5 | 228 | 50 |
| Sorghum | 22 |  | 60 | 10 | 1.0 | -0.4 | 60 | 10 | 75 |  | 152 | 1.9 | 1.5 | 31 | 14 |  | 5 | 178 | 50 |
| Soybean | 23 |  | 30 | 10 | 1.0 | -0.6 | 60 | 10 | 80 |  | 347 | 0.04 | 0.03 | 31 | 14 |  | 3 | 250 | 75 |
| Soybean | 24 |  | 55 | 10 | 1.0 | -0.6 | 60 | 10 | 80 |  | 347 | 0.04 | 0.03 | 31 | 14 |  | 4 | 300 | 50 |

Tech:

1: Alectra-resistant varieties and integrated crop management; 2: Disease-resistant varieties and integrated crop management; 3: Drought-tolerant varieties and integrated crop management; 4: Lines resistant to insects (aphid, thrips, pod sucking bug, maruca) and integrated pest management including biological control; 5: Low P-tolerant varieties and integrated crop management; 6: Drought-tolerant/resistant variety and short-duration (early- maturing) variety; 7: Moderately-resistant (for short-duration variety) and highly- resistant variety (for medium- and long-duration varieties) to early and late leaf spot; 8: Pre and postharvest aflatoxin management practices including Good Agricultural Practices (GAP); 9: Rosette-resistant variety; 10: Soil fertility management for P and other nutrients (N, Ca) including chemical/organic fertilizers application; 11: Varieties resistant to Fusarium wilt and Cercospora leaf spot; 12: Cleisto varieties and maintenance breeding to reduce varietal degeneration due to outcrossing; 13: Drought-tolerant varieties; 14: Weed control; 15: Intercropping-compatible varieties and integrated crop management options; 16: Varieties tolerant to pod borers, pod fly, pod bugs and integrated pest management; 17: Photo- and thermo-insensitive varieties; 18: Varieties tolerant to warm temperatures ; 19: Early-maturing varieties and hybrids with tolerance to drought; 20: Integrated crop management options for soil fertility, water management, Striga, intercropping; 21: Medium- to late-maturing anthracnose-resistant cultivars; 22: Varieties and hybrids with resistance to Striga; 23: Integrated soil fertility management; 24: Disease-resistant varieties and integrated pest management and crop management practices
