# Supplementary material for: Multi-dimensional impact assessment for priority setting of agricultural technologies: An application of TOPSIS for the drylands of sub-Saharan Africa and South Asia
Source: PLoS One. 2024 Nov 21;19(11):e0314007. doi: 10.1371/journal.pone.0314007 (PMC11581267; doi:10.1371/journal.pone.0314007)
Supplement: S5 Table — Tech: 1: Alectra-resistant varieties and integrated crop management; 2: Disease-resistant varieties and integrated crop management; 3: Drought-tolerant varieties and integrated crop management; 4: Lines resistant to insects (aphid, thrips, pod sucking bug, maruca) and integrated pest management including biological control; 5: Low P-tolerant varieties and integrated crop management; 6: Cleisto varieties and maintenance breeding to reduce varietal degeneration due to outcrossing; 7: Drought-tolerant varieties; 8: Varieties resistant to Fusarium wilt and Cercospora leaf spot; 9: Intercropping-compatible varieties and integrated crop management options; 10: Photo- and thermo-insensitive varieties; 11: Varieties tolerant to pod borers, pod fly, pod bugs and integrated pest management; 12: Early-maturing varieties and hybrids with tolerance to drought; 13: Integrated crop management options for soil fertility, water management, Striga, intercropping; 14: Medium- to late-maturing anthracnose-resistant cultivars; 15: Varieties and hybrids with resistance to Striga. (DOCX) [file pone.0314007.s005.docx]

S5 Table: research dissemination and adoption parameters for improved technologies – semi-arid southern Africa

| Crop | Tech |  | Farm changes | | | | | | |  | Macro-level parameters | | | | |  | Research and dissemination costs | | |
| --- | --- | --- | --- | --- | --- | --- | --- | --- | --- | --- | --- | --- | --- | --- | --- | --- | --- | --- | --- |
|  |  |  | Max adoption (%) | Adoption years | Supply elas. | Demand elas. | Yield change (%) | Cost change (%) | Probability of success |  | Price (US$/ton) | Quantity (mil. tons) | Area harvested (mil. ha) | Poverty headcount (mil. people) | Ag. GDP (bil. US$) |  | Res. Years | Res. Costs (‘000 US$/year) | Diss. Cost (US$/ha) |
| Cowpea | 1 |  | 30 | 10 | 1.0 | -0.5 | 40 | 20 | 80 |  | 500 | 0.1 | 0.2 | 12 | 5 |  | 10 | 120 | 50 |
| Cowpea | 2 |  | 30 | 10 | 1.0 | -0.5 | 50 | 20 | 80 |  | 500 | 0.1 | 0.2 | 12 | 5 |  | 10 | 250 | 50 |
| Cowpea | 3 |  | 30 | 10 | 1.0 | -0.5 | 70 | 10 | 80 |  | 500 | 0.1 | 0.2 | 12 | 5 |  | 10 | 200 | 50 |
| Cowpea | 4 |  | 30 | 10 | 1.0 | -0.5 | 80 | 20 | 80 |  | 500 | 0.1 | 0.2 | 12 | 5 |  | 10 | 250 | 50 |
| Cowpea | 5 |  | 25 | 10 | 1.0 | -0.5 | 60 | 20 | 80 |  | 500 | 0.1 | 0.2 | 12 | 5 |  | 10 | 200 | 50 |
| Pigeon pea | 6 |  | 50 | 10 | 1.0 | -0.5 | 50 | 30 | 80 |  | 494 | 0.0 | 0.0 | 5 | 0 |  | 5 | 121 | 50 |
| Pigeon pea | 7 |  | 50 | 10 | 1.0 | -0.5 | 50 | 30 | 70 |  | 494 | 0.0 | 0.0 | 5 | 0 |  | 5 | 71 | 50 |
| Pigeon pea | 8 |  | 50 | 10 | 1.0 | -0.5 | 60 | 30 | 90 |  | 494 | 0.0 | 0.0 | 5 | 0 |  | 5 | 161 | 50 |
| Pigeon pea | 9 |  | 50 | 10 | 1.0 | -0.5 | 50 | 30 | 90 |  | 494 | 0.0 | 0.0 | 5 | 0 |  | 5 | 91 | 75 |
| Pigeon pea | 10 |  | 50 | 10 | 1.0 | -0.5 | 30 | 30 | 90 |  | 494 | 0.0 | 0.0 | 5 | 0 |  | 3 | 181 | 50 |
| Pigeon pea | 11 |  | 50 | 10 | 1.0 | -0.5 | 40 | 30 | 50 |  | 494 | 0.0 | 0.0 | 5 | 0 |  | 6 | 81 | 50 |
| Sorghum | 12 |  | 60 | 10 | 1.0 | -0.4 | 80 | 10 | 90 |  | 152 | 0.2 | 0.3 | 15 | 6 |  | 5 | 275 | 50 |
| Sorghum | 13 |  | 50 | 10 | 1.0 | -0.4 | 60 | 10 | 75 |  | 152 | 0.2 | 0.3 | 15 | 6 |  | 3 | 178 | 75 |
| Sorghum | 14 |  | 60 | 10 | 1.0 | -0.4 | 60 | 10 | 75 |  | 152 | 0.2 | 0.3 | 15 | 6 |  | 5 | 278 | 50 |
| Sorghum | 15 |  | 60 | 10 | 1.0 | -0.4 | 60 | 10 | 75 |  | 152 | 0.2 | 0.3 | 15 | 6 |  | 5 | 228 | 50 |

Tech:

1: Alectra-resistant varieties and integrated crop management; 2: Disease-resistant varieties and integrated crop management; 3: Drought-tolerant varieties and integrated crop management; 4: Lines resistant to insects (aphid, thrips, pod sucking bug, maruca) and integrated pest management including biological control; 5: Low P-tolerant varieties and integrated crop management; 6: Cleisto varieties and maintenance breeding to reduce varietal degeneration due to outcrossing; 7: Drought-tolerant varieties; 8: Varieties resistant to Fusarium wilt and Cercospora leaf spot; 9: Intercropping-compatible varieties and integrated crop management options; 10: Photo- and thermo-insensitive varieties; 11: Varieties tolerant to pod borers, pod fly, pod bugs and integrated pest management; 12: Early-maturing varieties and hybrids with tolerance to drought; 13: Integrated crop management options for soil fertility, water management, Striga, intercropping; 14: Medium- to late-maturing anthracnose-resistant cultivars; 15: Varieties and hybrids with resistance to Striga
