# Supplementary material for: Multi-dimensional impact assessment for priority setting of agricultural technologies: An application of TOPSIS for the drylands of sub-Saharan Africa and South Asia
Source: PLoS One. 2024 Nov 21;19(11):e0314007. doi: 10.1371/journal.pone.0314007 (PMC11581267; doi:10.1371/journal.pone.0314007)
Supplement: S7 Table — Tech: 1: Varieties resistant to Fusarium wilt and root rots; 2: Drought-tolerant varieties; 3: Heat-tolerant varieties; 4: Pod borer-tolerant varieties and integrated pest management; 5; Herbicide-tolerant varieties to control weeds; 6: Genetically diverse dual-purpose hybrid parents/cultivars with high and stable yields; 7: Breeding for early-maturing, drought-tolerant OPVs and hybrids which can give stable yields under severe drought conditions; 8: Breeding for downy mildew- and smut-resistant dual-purpose OPVs and hybrid parents; 9: Integrated crop management; 10: Varieties resistant to diseases (foliar fungal, bud necrosis, soil borne); 11: Integrated crop management practices; 12: Low P-tolerant/efficient variety; 13: Pre and postharvest aflatoxin management practices including Good Agricultural Practices (GAP); 14: Soil fertility management for P and other nutrients (N, Ca) including chemical/organic fertilizers application; 15: Varieties resistant to wilt and root rots and integrated pest management; 16: Drought-tolerant varieties; 17: Heat-tolerant varieties; 18: Herbicide-tolerant varieties to control weeds; 19: Genetically diverse dual-purpose hybrid parents/cultivars with high and stable yields with disease resistance (downy mildew and blast); 20: Breeding for early-maturing, drought-tolerant hybrids which can give stable yields under severe drought conditions; 21: Integrated crop management; 22: Varieties resistant to Fusarium wilt and Cercospora leaf spot; 23: Varieties tolerant to pod borers, pod fly, pod bugs and integrated pest management; 24: Sterility mosaic disease-resistant varieties; 25: Intercropping compatible-varieties and integrated crop management options; 26: Drought-tolerant varieties; 27: Genetic base diversification; 28: Early-maturing varieties and hybrids with tolerance to drought; 29: Shoot fly-resistant cultivars; 30: Charcoal rot-resistant cultivars; 31: Cultivars tolerant to head bugs and grain mold. (DOCX) [file pone.0314007.s007.docx]

S7 Table: research dissemination and adoption parameters for improved technologies – semi-arid South Asia

| Crop | Tech |  | Farm changes | | | | | | |  | Macro-level parameters | | | | |  | Research and dissemination costs | | |
| --- | --- | --- | --- | --- | --- | --- | --- | --- | --- | --- | --- | --- | --- | --- | --- | --- | --- | --- | --- |
|  |  |  | Max adoption (%) | Adoption years | Supply elas. | Demand elas. | Yield change (%) | Cost change (%) | Probability of success |  | Price (US$/ton) | Quantity (mil. tons) | Area harvested (mil. ha) | Poverty headcount (mil. people) | Ag. GDP (bil. US$) |  | Res. Years | Res. Costs (‘000 US$/year) | Diss. Cost (US$/ha) |
| Chickpea | 1 |  | 40 | 10 | 1.0 | -0.5 | 40 | 20 | 90 |  | 577 | 3.3 | 4.4 | 148 | 133 |  | 5 | 300 | 50 |
| Chickpea | 2 |  | 40 | 10 | 1.0 | -0.5 | 35 | 20 | 80 |  | 577 | 3.3 | 4.4 | 148 | 133 |  | 5 | 300 | 50 |
| Chickpea | 3 |  | 40 | 10 | 1.0 | -0.5 | 20 | 20 | 90 |  | 577 | 3.3 | 4.4 | 148 | 133 |  | 5 | 300 | 50 |
| Chickpea | 4 |  | 40 | 10 | 1.0 | -0.5 | 30 | 20 | 50 |  | 577 | 3.3 | 4.4 | 148 | 133 |  | 10 | 300 | 50 |
| Chickpea | 5 |  | 40 | 10 | 1.0 | -0.5 | 35 | 20 | 80 |  | 577 | 3.3 | 4.4 | 148 | 133 |  | 5 | 300 | 50 |
| Fgr millet* | 6 |  | 40 | 10 | 1.0 | -0.5 | 30 | 20 | 70 |  | 303 | 1.2 | 0.8 | 149 | 133 |  | 10 | 300 | 50 |
| Fgr millet | 7 |  | 40 | 10 | 1.0 | -0.5 | 30 | 20 | 50 |  | 303 | 1.2 | 0.8 | 149 | 133 |  | 10 | 150 | 50 |
| Fgr millet | 8 |  | 40 | 10 | 1.0 | -0.5 | 30 | 20 | 80 |  | 303 | 1.2 | 0.8 | 149 | 133 |  | 10 | 100 | 50 |
| Fgr millet | 9 |  | 30 | 10 | 1.0 | -0.5 | 30 | 10 | 50 |  | 303 | 1.2 | 0.8 | 149 | 133 |  | 3 | 200 | 75 |
| Groundnuts | 10 |  | 40 | 10 | 1.0 | -0.5 | 30 | 30 | 90 |  | 811 | 1.9 | 1.4 | 148 | 133 |  | 8 | 511 | 50 |
| Groundnuts | 11 |  | 30 | 10 | 1.0 | -0.5 | 30 | 30 | 90 |  | 811 | 1.9 | 1.4 | 148 | 133 |  | 5 | 300 | 75 |
| Groundnuts | 12 |  | 40 | 10 | 1.0 | -0.5 | 25 | 30 | 40 |  | 811 | 1.9 | 1.4 | 148 | 133 |  | 8 | 106 | 50 |
| Groundnuts | 13 |  | 40 | 10 | 1.0 | -0.5 | 20 | 5 | 60 |  | 811 | 1.9 | 1.4 | 148 | 133 |  | 8 | 200 | 75 |
| Groundnuts | 14 |  | 40 | 7 | 1.0 | -0.5 | 40 | 30 | 70 |  | 811 | 1.9 | 1.4 | 148 | 133 |  | 5 | 200 | 75 |
| Lentil | 15 |  | 60 | 10 | 1.0 | -0.5 | 50 | 20 | 80 |  | 545 | 0.2 | 0.4 | 148 | 133 |  | 4 | 300 | 50 |
| Lentil | 16 |  | 60 | 10 | 1.0 | -0.5 | 60 | 20 | 80 |  | 545 | 0.2 | 0.4 | 148 | 133 |  | 5 | 300 | 50 |
| Lentil | 17 |  | 40 | 10 | 1.0 | -0.5 | 25 | 20 | 90 |  | 545 | 0.2 | 0.4 | 148 | 133 |  | 5 | 300 | 50 |
| Lentil | 18 |  | 30 | 10 | 1.0 | -0.5 | 35 | 20 | 90 |  | 545 | 0.2 | 0.4 | 148 | 133 |  | 5 | 300 | 50 |
| Pearl millet | 19 |  | 40 | 10 | 1.0 | -0.5 | 40 | 15 | 80 |  | 303 | 8.1 | 7.2 | 149 | 133 |  | 10 | 400 | 50 |
| Pearl millet | 20 |  | 40 | 10 | 1.0 | -0.5 | 40 | 15 | 50 |  | 303 | 8.1 | 7.2 | 149 | 133 |  | 10 | 150 | 50 |
| Pearl millet | 21 |  | 30 | 10 | 1.0 | -0.5 | 40 | 15 | 50 |  | 303 | 8.1 | 7.2 | 149 | 133 |  | 3 | 200 | 75 |
| Pigeon pea | 22 |  | 50 | 10 | 1.0 | -0.5 | 50 | 30 | 90 |  | 494 | 1.4 | 1.9 | 148 | 122 |  | 5 | 231 | 50 |
| Pigeon pea | 23 |  | 50 | 10 | 1.0 | -0.5 | 40 | 30 | 50 |  | 494 | 1.4 | 1.9 | 148 | 122 |  | 6 | 131 | 50 |
| Pigeon pea | 24 |  | 50 | 10 | 1.0 | -0.5 | 30 | 30 | 90 |  | 494 | 1.4 | 1.9 | 148 | 122 |  | 3 | 231 | 50 |
| Pigeon pea | 25 |  | 50 | 10 | 1.0 | -0.5 | 30 | 30 | 90 |  | 494 | 1.4 | 1.9 | 148 | 122 |  | 3 | 131 | 75 |
| Pigeon pea | 26 |  | 50 | 10 | 1.0 | -0.5 | 30 | 30 | 50 |  | 494 | 1.4 | 1.9 | 148 | 122 |  | 5 | 231 | 50 |
| Sorghum | 27 |  | 60 | 10 | 1.0 | -0.4 | 30 | 10 | 90 |  | 152 | 5.6 | 7.4 | 148 | 133 |  | 5 | 243 | 50 |
| Sorghum | 28 |  | 60 | 10 | 1.0 | -0.4 | 80 | 10 | 90 |  | 152 | 5.6 | 7.4 | 148 | 133 |  | 5 | 293 | 50 |
| Sorghum | 29 |  | 60 | 10 | 1.0 | -0.4 | 30 | 10 | 80 |  | 152 | 5.6 | 7.4 | 148 | 133 |  | 5 | 293 | 50 |
| Sorghum | 30 |  | 60 | 10 | 1.0 | -0.4 | 30 | 10 | 70 |  | 152 | 5.6 | 7.4 | 148 | 133 |  | 8 | 243 | 50 |
| Sorghum | 31 |  | 60 | 10 | 1.0 | -0.4 | 30 | 10 | 80 |  | 152 | 5.6 | 7.4 | 148 | 133 |  | 3 | 243 | 50 |

Tech:

1: Varieties resistant to Fusarium wilt and root rots; 2: Drought-tolerant varieties; 3: Heat-tolerant varieties; 4: Pod borer-tolerant varieties and integrated pest management; 5; Herbicide-tolerant varieties to control weeds; 6: Genetically diverse dual-purpose hybrid parents/cultivars with high and stable yields; 7: Breeding for early-maturing, drought-tolerant OPVs and hybrids which can give stable yields under severe drought conditions; 8: Breeding for downy mildew- and smut-resistant dual-purpose OPVs and hybrid parents; 9: Integrated crop management; 10: Varieties resistant to diseases (foliar fungal, bud necrosis, soil borne); 11: Integrated crop management practices; 12: Low P-tolerant/efficient variety; 13: Pre and postharvest aflatoxin management practices including Good Agricultural Practices (GAP); 14: Soil fertility management for P and other nutrients (N, Ca) including chemical/organic fertilizers application; 15: Varieties resistant to wilt and root rots and integrated pest management; 16: Drought-tolerant varieties; 17: Heat-tolerant varieties; 18: Herbicide-tolerant varieties to control weeds; 19: Genetically diverse dual-purpose hybrid parents/cultivars with high and stable yields with disease resistance (downy mildew and blast); 20: Breeding for early-maturing, drought-tolerant hybrids which can give stable yields under severe drought conditions; 21: Integrated crop management; 22: Varieties resistant to Fusarium wilt and Cercospora leaf spot; 23: Varieties tolerant to pod borers, pod fly, pod bugs and integrated pest management; 24: Sterility mosaic disease-resistant varieties; 25: Intercropping compatible-varieties and integrated crop management options; 26: Drought-tolerant varieties; 27: Genetic base diversification; 28: Early-maturing varieties and hybrids with tolerance to drought; 29: Shoot fly-resistant cultivars; 30: Charcoal rot-resistant cultivars; 31: Cultivars tolerant to head bugs and grain mold
