# Supplementary material for: Multi-dimensional impact assessment for priority setting of agricultural technologies: An application of TOPSIS for the drylands of sub-Saharan Africa and South Asia
Source: PLoS One. 2024 Nov 21;19(11):e0314007. doi: 10.1371/journal.pone.0314007 (PMC11581267; doi:10.1371/journal.pone.0314007)
Supplement: S8 Table — Tech: 1: Botrytis gray mold-resistant varieties; 2: Varieties resistant to Fusarium wilt and root rots; 3: Pod borer-tolerant varieties and integrated pest management; 4: Drought-tolerant varieties; 5: Stemphylium blight-resistant varieties and integrated pest management; 6: Herbicide-tolerant varieties to control weeds; 7: Drought-tolerant varieties; 8: Heat-tolerant varieties. (DOCX) [file pone.0314007.s008.docx]

S8 Table: research dissemination and adoption parameters for improved technologies – dry sub-humid south Asia

| Crop | Tech |  | Farm changes | | | | | | |  | Macro-level parameters | | | | |  | Research and dissemination costs | | |
| --- | --- | --- | --- | --- | --- | --- | --- | --- | --- | --- | --- | --- | --- | --- | --- | --- | --- | --- | --- |
|  |  |  | Max adoption (%) | Adoption years | Supply elas. | Demand elas. | Yield change (%) | Cost change (%) | Probability of success |  | Price (US$/ton) | Quantity (mil. tons) | Area harvested (mil. ha) | Poverty headcount (mil. people) | Ag. GDP (bil. US$) |  | Res. Years | Res. Costs (‘000 US$/year) | Diss. Cost (US$/ha) |
| Chickpea | 1 |  | 60 | 10 | 1.0 | -0.5 | 30 | 20 | 70 |  | 577 | 2.9 | 3.1 | 145 | 68 |  | 5 | 300 | 50 |
| Chickpea | 2 |  | 60 | 10 | 1.0 | -0.5 | 30 | 20 | 90 |  | 577 | 2.9 | 3.1 | 145 | 68 |  | 5 | 300 | 50 |
| Chickpea | 3 |  | 60 | 10 | 1.0 | -0.5 | 30 | 20 | 50 |  | 577 | 2.9 | 3.1 | 145 | 68 |  | 10 | 300 | 50 |
| Chickpea | 4 |  | 40 | 10 | 1.0 | -0.5 | 20 | 20 | 80 |  | 577 | 2.9 | 3.1 | 145 | 68 |  | 5 | 300 | 50 |
| Lentil | 5 |  | 60 | 10 | 1.0 | -0.5 | 20 | 10 | 70 |  | 545 | 0.5 | 0.7 | 145 | 68 |  | 4 | 300 | 50 |
| Lentil | 6 |  | 30 | 10 | 1.0 | -0.5 | 25 | 10 | 90 |  | 545 | 0.5 | 0.7 | 145 | 68 |  | 5 | 300 | 50 |
| Lentil | 7 |  | 60 | 10 | 1.0 | -0.5 | 20 | 10 | 80 |  | 545 | 0.5 | 0.7 | 145 | 68 |  | 5 | 300 | 50 |
| Lentil | 8 |  | 40 | 10 | 1.0 | -0.5 | 15 | 10 | 90 |  | 545 | 0.5 | 0.7 | 145 | 68 |  | 5 | 300 | 50 |

Tech:

1: Botrytis gray mold-resistant varieties; 2: Varieties resistant to Fusarium wilt and root rots; 3: Pod borer-tolerant varieties and integrated pest management; 4: Drought-tolerant varieties; 5: Stemphylium blight-resistant varieties and integrated pest management; 6: Herbicide-tolerant varieties to control weeds; 7: Drought-tolerant varieties; 8: Heat-tolerant varieties
