# Supplementary material for: Multi-dimensional impact assessment for priority setting of agricultural technologies: An application of TOPSIS for the drylands of sub-Saharan Africa and South Asia
Source: PLoS One. 2024 Nov 21;19(11):e0314007. doi: 10.1371/journal.pone.0314007 (PMC11581267; doi:10.1371/journal.pone.0314007)
Supplement: S9 Table — Tech: 1: Early-maturing varieties and hybrids with tolerance to drought; 2: Genetically diverse dual-purpose hybrid parents/cultivars with high and stable yields with disease resistance (downy mildew and blast); 3: Early-maturing, drought-tolerant hybrids which can give stable yields under severe drought conditions; 4: Insect- (aphid, thrips, pod sucking bug, maruca) resistant lines and integrated pest management including biological control; 5: Drought-tolerant varieties and integrated crop management; 6: Drought-tolerant/resistant variety and short-duration (early-maturing) variety; 7: Low P-tolerant varieties and integrated crop management; 8: Biological control of millet head miner and resistant hybrid parents; 9: Striga-resistant varieties and hybrids; 10: Pre and postharvest aflatoxin management practices including Good Agricultural Practices (GAP); 11: Striga-resistant varieties and integrated crop management; 12: Disease-resistant varieties and integrated crop management; 13: Integrated soil fertility management and identifying genotypes for low P tolerance; 14: Stem borer/midge-tolerant cultivars; 15: Cultivars adapted to low soil fertility/and with nutrient-use efficiency; 16: Rosette-resistant variety; 17: Moderately-resistant variety (for short-duration variety) and highly- resistant variety (for medium- and long-duration varieties) to early and late leaf spot; 18: OPVs with host plant resistance to Striga hermonthica; 19: Soil fertility management for P and other nutrients (N, Ca) including chemical/organic fertilizers application; 20: Low P-tolerant/efficient variety. For semi-arid west and central Africa, in the case of the first technology, the normalized index for the benefit-cost ratio criterion is 0.4532; it’s 0.6027 and -0.4722 for the poverty and child malnutrition criteria, respectively. The weighted normalized index for the benefit-cost ratio, poverty and child malnutrition criteria become 0.1763, 0.1664, and -0.1582, respectively. The matrix [file pone.0314007.s009.docx]

S9 Table: Estimated closeness index and ranking of technologies in semi-arid west and central Africa

| Crops | Tech |  | | Matrix aij: criteria values | | |  | Normalized decision matrix Rij | | |  | Normalized decision matrix Vij | | |  | Si+ | Si- | Ci |  | Rank | | | | |
| --- | --- | --- | --- | --- | --- | --- | --- | --- | --- | --- | --- | --- | --- | --- | --- | --- | --- | --- | --- | --- | --- | --- | --- | --- |
|  |  |  | | BCR | Pov | Maln |  | BCR | Pov | Maln |  | BCR | Pov | Maln |  |  |  |  |  | Ci | BCR | Pov | Maln |  |
| Sorghum | 1 |  | | 21 | 474550 | -37995 |  | 0.4261 | 0.5688 | -0.4722 |  | 0.1658 | 0.1570 | -0.1581 |  | 0.0000 | 0.2660 | 1.0000 |  | 1 | 1 | 1 | 1 |  |
| Pearl millet | 2 |  | | 14 | 308126 | -31888 |  | 0.2814 | 0.3693 | -0.3963 |  | 0.1095 | 0.1020 | -0.1327 |  | 0.0828 | 0.1894 | 0.6960 |  | 2 | 6 | 2 | 2 |  |
| Pearl millet | 3 |  | | 13 | 292181 | -28654 |  | 0.2553 | 0.3502 | -0.3561 |  | 0.0994 | 0.0967 | -0.1192 |  | 0.0978 | 0.1723 | 0.6379 |  | 3 | 7 | 3 | 3 |  |
| Cowpea | 4 |  | | 15 | 238387 | -25771 |  | 0.3119 | 0.2857 | -0.3203 |  | 0.1214 | 0.0789 | -0.1072 |  | 0.1033 | 0.1680 | 0.6192 |  | 4 | 4 | 5 | 4 |  |
| Cowpea | 5 |  | | 16 | 240504 | -24078 |  | 0.3187 | 0.2883 | -0.2992 |  | 0.1240 | 0.0796 | -0.1002 |  | 0.1053 | 0.1656 | 0.6113 |  | 5 | 3 | 4 | 5 |  |
| Groundnut | 6 |  | | 21 | 116790 | -2487 |  | 0.4243 | 0.1400 | -0.0309 |  | 0.1651 | 0.0386 | -0.0103 |  | 0.1893 | 0.1540 | 0.4485 |  | 6 | 2 | 9 | 15 |  |
| Cowpea | 7 |  | | 11 | 154000 | -18114 |  | 0.2295 | 0.1846 | -0.2251 |  | 0.0893 | 0.0510 | -0.0754 |  | 0.1547 | 0.1152 | 0.4268 |  | 7 | 8 | 8 | 7 |  |
| Pearl millet | 8 |  | | 6 | 195972 | -22226 |  | 0.1191 | 0.2349 | -0.2762 |  | 0.0464 | 0.0648 | -0.0925 |  | 0.1645 | 0.1144 | 0.4102 |  | 8 | 15 | 6 | 6 |  |
| Sorghum | 9 |  | | 9 | 193977 | -16189 |  | 0.1755 | 0.2325 | -0.2012 |  | 0.0683 | 0.0642 | -0.0674 |  | 0.1624 | 0.1042 | 0.3908 |  | 9 | 9 | 7 | 8 |  |
| Groundnut | 10 |  | | 14 | 66247 | 0 |  | 0.2818 | 0.0794 | 0.0000 |  | 0.1096 | 0.0219 | 0.0000 |  | 0.2154 | 0.0959 | 0.3082 |  | 10 | 5 | 15 | 19 |  |
| Cowpea | 11 |  | | 6 | 94526 | -13836 |  | 0.1292 | 0.1133 | -0.1719 |  | 0.0503 | 0.0313 | -0.0576 |  | 0.1982 | 0.0723 | 0.2674 |  | 11 | 12 | 13 | 10 |  |
| Cowpea | 12 |  | | 6 | 94526 | -13836 |  | 0.1241 | 0.1133 | -0.1719 |  | 0.0483 | 0.0313 | -0.0576 |  | 0.1993 | 0.0714 | 0.2637 |  | 12 | 13 | 13 | 10 |  |
| Pearl millet | 13 |  | | 3 | 105262 | -14235 |  | 0.0652 | 0.1262 | -0.1769 |  | 0.0254 | 0.0348 | -0.0592 |  | 0.2108 | 0.0670 | 0.2413 |  | 13 | 18 | 11 | 9 |  |
| Sorghum | 14 |  | | 5 | 112884 | -9958 |  | 0.1026 | 0.1353 | -0.1237 |  | 0.0399 | 0.0374 | -0.0414 |  | 0.2092 | 0.0580 | 0.2171 |  | 14 | 16 | 10 | 12 |  |
| Sorghum | 15 |  | | 4 | 97766 | -8651 |  | 0.0723 | 0.1172 | -0.1075 |  | 0.0281 | 0.0324 | -0.0360 |  | 0.2223 | 0.0470 | 0.1744 |  | 15 | 17 | 12 | 14 |  |
| Groundnut | 16 |  | | 8 | 39175 | -1996 |  | 0.1585 | 0.0470 | -0.0248 |  | 0.0617 | 0.0130 | -0.0083 |  | 0.2324 | 0.0479 | 0.1708 |  | 16 | 10 | 18 | 17 |  |
| Groundnut | 17 |  | | 7 | 49574 | -2433 |  | 0.1438 | 0.0594 | -0.0302 |  | 0.0560 | 0.0164 | -0.0101 |  | 0.2318 | 0.0435 | 0.1580 |  | 17 | 11 | 16 | 16 |  |
| Pearl millet | 18 |  | | 2 | 42584 | -9467 |  | 0.0390 | 0.0510 | -0.1177 |  | 0.0152 | 0.0141 | -0.0394 |  | 0.2392 | 0.0404 | 0.1445 |  | 18 | 20 | 17 | 13 |  |
| Groundnut | 19 |  | | 6 | 28852 | 0 |  | 0.1235 | 0.0346 | 0.0000 |  | 0.0481 | 0.0095 | 0.0000 |  | 0.2462 | 0.0332 | 0.1188 |  | 19 | 14 | 19 | 19 |  |
| Groundnut | 20 |  | | 3 | 15432 | -747 |  | 0.0588 | 0.0185 | -0.0093 |  | 0.0229 | 0.0051 | -0.0031 |  | 0.2598 | 0.0083 | 0.0310 |  | 20 | 19 | 20 | 18 |  |
| Estimated weights: | | | 0.3891 | | 0.2761 | 0.3348 |  |  |  |  |  |  |  |  |  |  |  |  |  |  |  |  |  |  |
| Positive-ideal solution: | | |  | |  |  |  |  |  |  |  | 0.1658 | 0.1570 | -0.1581 |  |  |  |  |  |  |  |  |  |  |
| Negative-ideal solution: | | | | | |  |  |  |  |  |  | 0.0152 | 0.0051 | 0.0000 |  |  |  |  |  |  |  |  |  |  |

Tech:

1: Early-maturing varieties and hybrids with tolerance to drought; 2: Genetically diverse dual-purpose hybrid parents/cultivars with high and stable yields with disease resistance (downy mildew and blast); 3: Early-maturing, drought-tolerant hybrids which can give stable yields under severe drought conditions; 4: Insect- (aphid, thrips, pod sucking bug, maruca) resistant lines and integrated pest management including biological control; 5: Drought-tolerant varieties and integrated crop management; 6: Drought-tolerant/resistant variety and short-duration (early-maturing) variety; 7: Low P-tolerant varieties and integrated crop management; 8: Biological control of millet head miner and resistant hybrid parents; 9: Striga-resistant varieties and hybrids; 10: Pre and postharvest aflatoxin management practices including Good Agricultural Practices (GAP); 11: Striga-resistant varieties and integrated crop management; 12: Disease-resistant varieties and integrated crop management; 13: Integrated soil fertility management and identifying genotypes for low P tolerance; 14: Stem borer/midge-tolerant cultivars; 15: Cultivars adapted to low soil fertility/and with nutrient-use efficiency; 16: Rosette-resistant variety; 17: Moderately-resistant variety (for short-duration variety) and highly- resistant variety (for medium- and long-duration varieties) to early and late leaf spot; 18: OPVs with host plant resistance to Striga hermonthica; 19: Soil fertility management for P and other nutrients (N, Ca) including chemical/organic fertilizers application; 20: Low P-tolerant/efficient variety

For semi-arid west and central Africa, in the case of the first technology, the normalized index for the benefit-cost ratio criterion is 0.4532; it’s 0.6027 and -0.4722 for the poverty and child malnutrition criteria, respectively. The weighted normalized index for the benefit-cost ratio, poverty and child malnutrition criteria become 0.1763, 0.1664, and -0.1582, respectively. The matrix for the positive-ideal solution is [0.1763; 0.1664; -0.1581] whereas the matrix for the negative -ideal solution is [0.0153; 0.0048; 0.0000]. The Euclidean distances to the positive- and negative-ideal solutions for the first technology are computed as follow:

$S_{1}^{+}=\sqrt{\sum_{j=1}^{3} \left( v_{1j}-v_{1j}^{+} \right)^{2}}$ ; $S_{1}^{+}=\left( 0.1763-0.1763 \right)^{2}+\left( 0.1664-0.1664 \right)^{2}+{(-0.1581+(-0.1581))}^{2}=0$

$S_{1}^{-}=\sqrt{\sum_{j=1}^{3} \left( v_{1j}-v_{1j}^{-} \right)^{2}}$ ; $S_{1}^{+}=\left( 0.1763-0.0153 \right)^{2}+\left( 0.1664-0.0048 \right)^{2}+{(-0.1581+0.0000)}^{2}=0.2776$

The closeness index for the first technology is then computed as: $C_{i}^{+}=\frac{S_{i}^{-}}{S_{i}^{-}+S_{i}^{+}}=\frac{0.2776}{(0.2776+0)}=1$
