# Supplementary material for: Multi-dimensional impact assessment for priority setting of agricultural technologies: An application of TOPSIS for the drylands of sub-Saharan Africa and South Asia
Source: PLoS One. 2024 Nov 21;19(11):e0314007. doi: 10.1371/journal.pone.0314007 (PMC11581267; doi:10.1371/journal.pone.0314007)
Supplement: S10 Table — Tech: 1: Insect- (aphid, thrips, pod sucking bug, maruca) resistant lines and integrated pest management including biological control; 2: Medium- to late-maturing anthracnose-resistant cultivars; 3: Striga-resistant varieties and hybrids; 4: Drought-tolerant varieties and integrated crop management; 5: Low P-tolerant varieties and integrated crop management; 6: Drought-tolerant/resistant variety and short-duration (early- maturing) variety; 7: Striga-resistant varieties and integrated crop management; 8: Disease-resistant varieties and integrated crop management; 9: Pre and postharvest aflatoxin management practices including Good Agricultural Practices (GAP); 10: Drought-tolerant varieties and crop management and water conservation practices; 11: Head bugs- and grain mold-tolerant cultivars; 12: Disease-resistant varieties and integrated pest management and crop management practices; 13: Cultivars adapted to low soil fertility/and with nutrient-use efficiency;14: Increased plant population; 15: Use of inoculant and fertilizers especially Phosphorus; 16: Rosette-resistant variety; 17: Stem borer/midge-tolerant cultivars; 18: Moderately-resistant variety (for short-duration variety) and highly-resistant variety (for medium- and long-duration varieties) to early and late leaf spot; 19: Soil fertility management for P and other nutrients (N, Ca) including chemical/organic fertilizers application. (DOCX) [file pone.0314007.s010.docx]

S10 Table: Estimated closeness index and ranking of technologies in dry sub-humid west and central Africa

| Crops | Tech |  | Matrix aij: criteria values | | |  | Normalized decision matrix Rij | | |  | Normalized decision matrix Vij | | |  | Si+ | Si- | Ci |  | Rank | | | |
| --- | --- | --- | --- | --- | --- | --- | --- | --- | --- | --- | --- | --- | --- | --- | --- | --- | --- | --- | --- | --- | --- | --- |
|  |  |  | BCR | Pov | Maln |  | BCR | Pov | Maln |  | BCR | Pov | Maln |  |  |  |  |  | Ci | BCR | Pov | Maln |
| Cowpea | 1 |  | 15 | 40382 | -11295 |  | 0.3314 | 0.3265 | -0.3664 |  | 0.1427 | 0.0785 | -0.1205 |  | 0.0579 | 0.1870 | 0.7635 |  | 1 | 2 | 4 | 3 |
| Sorghum | 2 |  | 9 | 53130 | -12216 |  | 0.1951 | 0.4296 | -0.3963 |  | 0.0840 | 0.1033 | -0.1303 |  | 0.0755 | 0.1738 | 0.6971 |  | 2 | 11 | 1 | 2 |
| Sorghum | 3 |  | 7 | 41087 | -16189 |  | 0.1502 | 0.3322 | -0.5252 |  | 0.0647 | 0.0799 | -0.1727 |  | 0.0851 | 0.1929 | 0.6938 |  | 3 | 14 | 3 | 1 |
| Cowpea | 4 |  | 15 | 36759 | -9389 |  | 0.3139 | 0.2972 | -0.3046 |  | 0.1352 | 0.0715 | -0.1001 |  | 0.0800 | 0.1664 | 0.6753 |  | 4 | 4 | 5 | 4 |
| Cowpea | 5 |  | 12 | 27043 | -8190 |  | 0.2505 | 0.2186 | -0.2657 |  | 0.1079 | 0.0526 | -0.0873 |  | 0.1065 | 0.1330 | 0.5552 |  | 5 | 8 | 8 | 5 |
| Groundnut | 6 |  | 16 | 44378 | -2139 |  | 0.3403 | 0.3588 | -0.0694 |  | 0.1465 | 0.0863 | -0.0228 |  | 0.1508 | 0.1509 | 0.5001 |  | 6 | 1 | 2 | 11 |
| Cowpea | 7 |  | 9 | 21611 | -7491 |  | 0.2010 | 0.1747 | -0.2430 |  | 0.0866 | 0.0420 | -0.0799 |  | 0.1263 | 0.1108 | 0.4672 |  | 7 | 10 | 10 | 6 |
| Cowpea | 8 |  | 8 | 21611 | -7491 |  | 0.1777 | 0.1747 | -0.2430 |  | 0.0766 | 0.0420 | -0.0799 |  | 0.1314 | 0.1048 | 0.4438 |  | 8 | 12 | 10 | 6 |
| Groundnut | 9 |  | 14 | 33064 | -925 |  | 0.2966 | 0.2673 | -0.0300 |  | 0.1278 | 0.0643 | -0.0099 |  | 0.1685 | 0.1225 | 0.4210 |  | 9 | 6 | 6 | 16 |
| Soybean | 10 |  | 15 | 9551 | 131 |  | 0.3196 | 0.0772 | 0.0042 |  | 0.1377 | 0.0186 | 0.0014 |  | 0.1938 | 0.1199 | 0.3823 |  | 10 | 3 | 17 | 19 |
| Sorghum | 11 |  | 4 | 27714 | -6918 |  | 0.0930 | 0.2241 | -0.2244 |  | 0.0401 | 0.0539 | -0.0738 |  | 0.1535 | 0.0892 | 0.3677 |  | 11 | 17 | 7 | 8 |
| Soybean | 12 |  | 14 | 7179 | 108 |  | 0.2968 | 0.0580 | 0.0035 |  | 0.1278 | 0.0140 | 0.0012 |  | 0.1963 | 0.1099 | 0.3589 |  | 12 | 5 | 18 | 18 |
| Sorghum | 13 |  | 4 | 26923 | -6763 |  | 0.0775 | 0.2177 | -0.2194 |  | 0.0334 | 0.0524 | -0.0721 |  | 0.1597 | 0.0857 | 0.3491 |  | 13 | 18 | 9 | 9 |
| Groundnut | 14 |  | 11 | 20406 | -1584 |  | 0.2380 | 0.1650 | -0.0514 |  | 0.1025 | 0.0397 | -0.0169 |  | 0.1739 | 0.0911 | 0.3437 |  | 14 | 9 | 12 | 14 |
| Soybean | 15 |  | 12 | 5762 | 107 |  | 0.2545 | 0.0466 | 0.0035 |  | 0.1096 | 0.0112 | 0.0011 |  | 0.2001 | 0.0916 | 0.3141 |  | 15 | 7 | 19 | 17 |
| Groundnut | 16 |  | 8 | 19546 | -1935 |  | 0.1668 | 0.1580 | -0.0628 |  | 0.0718 | 0.0380 | -0.0206 |  | 0.1815 | 0.0641 | 0.2610 |  | 16 | 13 | 13 | 12 |
| Sorghum | 17 |  | 2 | 11440 | -5843 |  | 0.0417 | 0.0925 | -0.1895 |  | 0.0179 | 0.0223 | -0.0623 |  | 0.1879 | 0.0647 | 0.2561 |  | 17 | 19 | 16 | 10 |
| Groundnut | 18 |  | 7 | 16450 | -1596 |  | 0.1404 | 0.1330 | -0.0518 |  | 0.0605 | 0.0320 | -0.0170 |  | 0.1916 | 0.0508 | 0.2095 |  | 18 | 15 | 14 | 13 |
| Groundnuts | 19 |  | 6 | 14400 | -1386 |  | 0.1301 | 0.1164 | -0.0450 |  | 0.0560 | 0.0280 | -0.0148 |  | 0.1970 | 0.0446 | 0.1848 |  | 19 | 16 | 15 | 15 |
| Estimated weights: | | | 0.4307 | 0.2405 | 0.3288 |  |  |  |  |  |  |  |  |  |  |  |  |  |  |  |  |  |
| Positive-ideal solution: | | |  |  |  |  |  |  |  |  | 0.1465 | 0.1033 | -0.1727 |  |  |  |  |  |  |  |  |  |
| Negative-ideal solution: | | | | |  |  |  |  |  |  | 0.0179 | 0.0112 | 0.0014 |  |  |  |  |  |  |  |  |  |

Tech:

1: Insect- (aphid, thrips, pod sucking bug, maruca) resistant lines and integrated pest management including biological control; 2: Medium- to late-maturing anthracnose-resistant cultivars; 3: Striga-resistant varieties and hybrids; 4: Drought-tolerant varieties and integrated crop management; 5: Low P-tolerant varieties and integrated crop management; 6: Drought-tolerant/resistant variety and short-duration (early- maturing) variety; 7: Striga-resistant varieties and integrated crop management; 8: Disease-resistant varieties and integrated crop management; 9: Pre and postharvest aflatoxin management practices including Good Agricultural Practices (GAP); 10: Drought-tolerant varieties and crop management and water conservation practices; 11: Head bugs- and grain mold-tolerant cultivars; 12: Disease-resistant varieties and integrated pest management and crop management practices; 13: Cultivars adapted to low soil fertility/and with nutrient-use efficiency;14: Increased plant population; 15: Use of inoculant and fertilizers especially Phosphorus; 16: Rosette-resistant variety; 17: Stem borer/midge-tolerant cultivars; 18: Moderately-resistant variety (for short-duration variety) and highly-resistant variety (for medium- and long-duration varieties) to early and late leaf spot; 19: Soil fertility management for P and other nutrients (N, Ca) including chemical/organic fertilizers application
