# Supplementary material for: Multi-dimensional impact assessment for priority setting of agricultural technologies: An application of TOPSIS for the drylands of sub-Saharan Africa and South Asia
Source: PLoS One. 2024 Nov 21;19(11):e0314007. doi: 10.1371/journal.pone.0314007 (PMC11581267; doi:10.1371/journal.pone.0314007)
Supplement: S11 Table — Tech: 1: Intercropping compatible-varieties and integrated crop management options; 2: Early-maturing varieties and hybrids with tolerance to drought; 3: Fusarium wilt- and Cercospora leaf spot-resistant varieties; 4: Ascochyta blight-resistant varieties; 5: Photo- and thermo-insensitive varieties;6: Stem borer/midge-tolerant cultivars; 7: Striga-resistant varieties and hybrids; 8: Drought-tolerant/resistant variety and short-duration (early-maturing) variety 9: Fusarium wilt- and root rots-resistant varieties; 10: Validate and promote water management options; fertilizer regimes; 11: Cleisto varieties and maintenance breeding to reduce varietal degeneration due to out crossing; 12: Drought-tolerant varieties; 13: Integrated crop management options for soil fertility, water management, Striga, intercropping; 14: Pre and postharvest aflatoxin management practices including Good Agricultural Practices (GAP); 15: Varieties and hybrid parents with good establishment and respond well to drought especially terminal drought; 16: Drought-tolerant varieties; 17: Validate and promote water management options; fertilizer regimes; 18: Varieties tolerant to pod borers, pod fly, pod bugs and integrated pest management; 19: Varieties and hybrid parents with good establishment and that respond well to drought, especially terminal drought; 20: Early-maturing, drought-tolerant hybrids which can give stable yields under severe drought conditions; 21: Drought-tolerant varieties and integrated crop management; 22: Insect- (aphid, thrips, pod sucking bug, maruca) resistant lines and integrated crop management; 23: Rosette-resistant variety; 24: Genetically diverse dual-purpose hybrid parents/cultivars with high and stable yields with disease resistance (downy mildew and blast); 25: Early-maturing, drought-tolerant OPVs and hybrids which can give stable yields under severe drought conditions; 26: Moderately-resistant variety (for short-duration variety) and highly-resistant variety (for m [file pone.0314007.s011.docx]

S11 Table: Estimated closeness index and ranking of technologies in semi-arid eastern Africa

| Crops | Tech |  | | Matrix aij: criteria values | | |  | Normalized decision matrix Rij | | |  | Normalized decision matrix Vij | | |  | Si+ | Si- | Ci |  | Rank | | | | |
| --- | --- | --- | --- | --- | --- | --- | --- | --- | --- | --- | --- | --- | --- | --- | --- | --- | --- | --- | --- | --- | --- | --- | --- | --- |
|  |  |  | | BCR | Pov | Maln |  | BCR | Pov | Maln |  | BCR | Pov | Maln |  |  |  |  |  | Ci | BCR | Pov | Maln |  |
| Pigeon pea | 1 |  | | 7 | 5634 | -207417 |  | 0.1823 | 0.0375 | -0.9993 |  | 0.0850 | 0.0061 | -0.3703 |  | 0.1203 | 0.3775 | 0.7583 |  | 1 | 10 | 14 | 1 |  |
| Sorghum | 2 |  | | 14 | 91273 | -4897 |  | 0.3370 | 0.6080 | -0.0236 |  | 0.1571 | 0.0992 | -0.0087 |  | 0.3616 | 0.1743 | 0.3253 |  | 2 | 2 | 1 | 2 |  |
| Pigeon pea | 3 |  | | 14 | 7811 | 137 |  | 0.3456 | 0.0520 | 0.0007 |  | 0.1611 | 0.0085 | 0.0002 |  | 0.3815 | 0.1475 | 0.2788 |  | 3 | 1 | 10 | 31 |  |
| Chickpea | 4 |  | | 11 | 1298 | 138 |  | 0.2806 | 0.0086 | 0.0007 |  | 0.1308 | 0.0014 | 0.0002 |  | 0.3845 | 0.1169 | 0.2332 |  | 4 | 3 | 30 | 32 |  |
| Pigeon pea | 5 |  | | 11 | 5634 | 114 |  | 0.2768 | 0.0375 | 0.0006 |  | 0.1291 | 0.0061 | 0.0002 |  | 0.3834 | 0.1153 | 0.2312 |  | 5 | 4 | 13 | 29 |  |
| Sorghum | 6 |  | | 7 | 71286 | -3939 |  | 0.1796 | 0.4749 | -0.0190 |  | 0.0837 | 0.0775 | -0.0070 |  | 0.3721 | 0.1043 | 0.2190 |  | 6 | 11 | 2 | 3 |  |
| Sorghum | 7 |  | | 7 | 71286 | -3939 |  | 0.1787 | 0.4749 | -0.0190 |  | 0.0833 | 0.0775 | -0.0070 |  | 0.3722 | 0.1041 | 0.2185 |  | 7 | 12 | 2 | 3 |  |
| Groundnut | 8 |  | | 10 | 22628 | 146 |  | 0.2485 | 0.1507 | 0.0007 |  | 0.1159 | 0.0246 | 0.0003 |  | 0.3807 | 0.1048 | 0.2159 |  | 8 | 5 | 5 | 34 |  |
| Chickpea | 9 |  | | 9 | 1380 | 192 |  | 0.2303 | 0.0092 | 0.0009 |  | 0.1074 | 0.0015 | 0.0003 |  | 0.3871 | 0.0935 | 0.1945 |  | 9 | 6 | 29 | 37 |  |
| Finger millet | 10 |  | | 9 | 2558 | 77 |  | 0.2238 | 0.0170 | 0.0004 |  | 0.1043 | 0.0028 | 0.0001 |  | 0.3870 | 0.0905 | 0.1895 |  | 10 | 7 | 24 | 22 |  |
| Pigeon pea | 11 |  | | 9 | 4996 | 103 |  | 0.2214 | 0.0333 | 0.0005 |  | 0.1032 | 0.0054 | 0.0002 |  | 0.3866 | 0.0895 | 0.1879 |  | 11 | 8 | 15 | 25 |  |
| Pigeon pea | 12 |  | | 8 | 4360 | 95 |  | 0.2063 | 0.0290 | 0.0005 |  | 0.0962 | 0.0047 | 0.0002 |  | 0.3878 | 0.0824 | 0.1752 |  | 12 | 9 | 17 | 24 |  |
| Sorghum | 13 |  | | 5 | 47009 | -2565 |  | 0.1259 | 0.3131 | -0.0124 |  | 0.0587 | 0.0511 | -0.0046 |  | 0.3829 | 0.0679 | 0.1507 |  | 13 | 19 | 4 | 5 |  |
| Groundnut | 14 |  | | 7 | 2739 | 89 |  | 0.1769 | 0.0182 | 0.0004 |  | 0.0825 | 0.0030 | 0.0002 |  | 0.3908 | 0.0686 | 0.1494 |  | 14 | 13 | 23 | 23 |  |
| Pearl millet | 15 |  | | 6 | 18993 | 25 |  | 0.1484 | 0.1265 | 0.0001 |  | 0.0692 | 0.0206 | 0.0000 |  | 0.3896 | 0.0589 | 0.1314 |  | 15 | 15 | 6 | 7 |  |
| Chickpea | 16 |  | | 6 | 1224 | 168 |  | 0.1562 | 0.0082 | 0.0008 |  | 0.0728 | 0.0013 | 0.0003 |  | 0.3934 | 0.0589 | 0.1302 |  | 16 | 14 | 31 | 36 |  |
| Pearl millet | 17 |  | | 5 | 12370 | 72 |  | 0.1324 | 0.0824 | 0.0003 |  | 0.0617 | 0.0134 | 0.0001 |  | 0.3930 | 0.0496 | 0.1121 |  | 17 | 16 | 8 | 21 |  |
| Pigeon pea | 18 |  | | 5 | 3099 | 67 |  | 0.1315 | 0.0206 | 0.0003 |  | 0.0613 | 0.0034 | 0.0001 |  | 0.3955 | 0.0475 | 0.1072 |  | 18 | 17 | 20 | 20 |  |
| Finger millet | 19 |  | | 5 | 3041 | 25 |  | 0.1308 | 0.0203 | 0.0001 |  | 0.0610 | 0.0033 | 0.0000 |  | 0.3955 | 0.0472 | 0.1065 |  | 19 | 18 | 21 | 10 |  |
| Pearl millet | 20 |  | | 5 | 16567 | 25 |  | 0.1188 | 0.1104 | 0.0001 |  | 0.0554 | 0.0180 | 0.0000 |  | 0.3936 | 0.0451 | 0.1028 |  | 20 | 20 | 7 | 7 |  |
| Cowpea | 21 |  | | 5 | 4197 | 167 |  | 0.1140 | 0.0280 | 0.0008 |  | 0.0531 | 0.0046 | 0.0003 |  | 0.3975 | 0.0395 | 0.0903 |  | 21 | 21 | 18 | 35 |  |
| Cowpea | 22 |  | | 4 | 4555 | 196 |  | 0.1056 | 0.0303 | 0.0009 |  | 0.0493 | 0.0050 | 0.0004 |  | 0.3985 | 0.0357 | 0.0821 |  | 22 | 22 | 16 | 38 |  |
| Groundnut | 23 |  | | 4 | 7597 | 121 |  | 0.1027 | 0.0506 | 0.0006 |  | 0.0479 | 0.0083 | 0.0002 |  | 0.3980 | 0.0349 | 0.0807 |  | 23 | 23 | 11 | 30 |  |
| Pearl millet | 24 |  | | 4 | 11759 | 26 |  | 0.0921 | 0.0783 | 0.0001 |  | 0.0429 | 0.0128 | 0.0000 |  | 0.3983 | 0.0316 | 0.0735 |  | 24 | 24 | 9 | 11 |  |
| Finger millet | 25 |  | | 3 | 2555 | 26 |  | 0.0834 | 0.0170 | 0.0001 |  | 0.0389 | 0.0028 | 0.0000 |  | 0.4018 | 0.0251 | 0.0588 |  | 25 | 25 | 25 | 12 |  |
| Groundnut | 26 |  | | 3 | 7189 | 113 |  | 0.0791 | 0.0479 | 0.0005 |  | 0.0369 | 0.0078 | 0.0002 |  | 0.4014 | 0.0242 | 0.0568 |  | 26 | 27 | 12 | 28 |  |
| Groundnut | 27 |  | | 3 | 1193 | 24 |  | 0.0800 | 0.0079 | 0.0001 |  | 0.0373 | 0.0013 | 0.0000 |  | 0.4026 | 0.0234 | 0.0550 |  | 27 | 26 | 33 | 6 |  |
| Finger millet | 28 |  | | 3 | 1816 | 25 |  | 0.0788 | 0.0121 | 0.0001 |  | 0.0367 | 0.0020 | 0.0000 |  | 0.4026 | 0.0229 | 0.0538 |  | 28 | 28 | 28 | 9 |  |
| Cowpea | 29 |  | | 3 | 2844 | 140 |  | 0.0773 | 0.0189 | 0.0007 |  | 0.0361 | 0.0031 | 0.0003 |  | 0.4028 | 0.0223 | 0.0525 |  | 29 | 29 | 22 | 33 |  |
| Cowpea | 30 |  | | 3 | 1986 | 113 |  | 0.0743 | 0.0132 | 0.0005 |  | 0.0346 | 0.0022 | 0.0002 |  | 0.4034 | 0.0208 | 0.0491 |  | 30 | 30 | 26 | 26 |  |
| Chickpea | 31 |  | | 3 | 365 | 56 |  | 0.0682 | 0.0024 | 0.0003 |  | 0.0318 | 0.0004 | 0.0001 |  | 0.4046 | 0.0179 | 0.0424 |  | 31 | 31 | 35 | 18 |  |
| Finger millet | 32 |  | | 2 | 1198 | 59 |  | 0.0572 | 0.0080 | 0.0003 |  | 0.0267 | 0.0013 | 0.0001 |  | 0.4061 | 0.0128 | 0.0306 |  | 32 | 32 | 32 | 19 |  |
| Lentil | 33 |  | | 2 | 687 | 55 |  | 0.0521 | 0.0046 | 0.0003 |  | 0.0243 | 0.0007 | 0.0001 |  | 0.4070 | 0.0104 | 0.0249 |  | 33 | 33 | 34 | 17 |  |
| Groundnut | 34 |  | | 2 | 3188 | 53 |  | 0.0457 | 0.0212 | 0.0003 |  | 0.0213 | 0.0035 | 0.0001 |  | 0.4073 | 0.0081 | 0.0194 |  | 34 | 35 | 19 | 16 |  |
| Cowpea | 35 |  | | 2 | 1986 | 113 |  | 0.0462 | 0.0132 | 0.0005 |  | 0.0215 | 0.0022 | 0.0002 |  | 0.4077 | 0.0079 | 0.0189 |  | 35 | 34 | 26 | 26 |  |
| Lentil | 36 |  | | 1 | 264 | 37 |  | 0.0309 | 0.0018 | 0.0002 |  | 0.0144 | 0.0003 | 0.0001 |  | 0.4105 | 0.0006 | 0.0014 |  | 36 | 36 | 38 | 13 |  |
| Lentil | 37 |  | | 1 | 330 | 47 |  | 0.0298 | 0.0022 | 0.0002 |  | 0.0139 | 0.0004 | 0.0001 |  | 0.4107 | 0.0003 | 0.0007 |  | 37 | 37 | 36 | 14 |  |
| Lentil | 38 |  | | 1 | 330 | 47 |  | 0.0298 | 0.0022 | 0.0002 |  | 0.0139 | 0.0004 | 0.0001 |  | 0.4107 | 0.0003 | 0.0007 |  | 37 | 37 | 36 | 14 |  |
| Estimated weights: | | | 0.4662 | | 0.1632 | 0.3706 |  |  |  |  |  |  |  |  |  |  |  |  |  |  |  |  |  |  |
| Positive-ideal solution: | | |  | |  |  |  |  |  |  |  | 0.1611 | 0.0992 | -0.3703 |  |  |  |  |  |  |  |  |  |  |
| Negative-ideal solution: | | | | | |  |  |  |  |  |  | 0.0139 | 0.0003 | 0.0004 |  |  |  |  |  |  |  |  |  |  |

Tech:

1: Intercropping compatible-varieties and integrated crop management options; 2: Early-maturing varieties and hybrids with tolerance to drought; 3: Fusarium wilt- and Cercospora leaf spot-resistant varieties; 4: Ascochyta blight-resistant varieties; 5: Photo- and thermo-insensitive varieties;6: Stem borer/midge-tolerant cultivars; 7: Striga-resistant varieties and hybrids; 8: Drought-tolerant/resistant variety and short-duration (early-maturing) variety 9: Fusarium wilt- and root rots-resistant varieties; 10: Validate and promote water management options; fertilizer regimes; 11: Cleisto varieties and maintenance breeding to reduce varietal degeneration due to out crossing; 12: Drought-tolerant varieties; 13: Integrated crop management options for soil fertility, water management, Striga, intercropping; 14: Pre and postharvest aflatoxin management practices including Good Agricultural Practices (GAP); 15: Varieties and hybrid parents with good establishment and respond well to drought especially terminal drought; 16: Drought-tolerant varieties; 17: Validate and promote water management options; fertilizer regimes; 18: Varieties tolerant to pod borers, pod fly, pod bugs and integrated pest management; 19: Varieties and hybrid parents with good establishment and that respond well to drought, especially terminal drought; 20: Early-maturing, drought-tolerant hybrids which can give stable yields under severe drought conditions; 21: Drought-tolerant varieties and integrated crop management; 22: Insect- (aphid, thrips, pod sucking bug, maruca) resistant lines and integrated crop management; 23: Rosette-resistant variety; 24: Genetically diverse dual-purpose hybrid parents/cultivars with high and stable yields with disease resistance (downy mildew and blast); 25: Early-maturing, drought-tolerant OPVs and hybrids which can give stable yields under severe drought conditions; 26: Moderately-resistant variety (for short-duration variety) and highly-resistant variety (for medium- and long-duration varieties) to early and late leaf spot; 27: Soil fertility management for P and other nutrients (N, Ca) including chemical/organic fertilizers application; 28: Downy mildew- and smut-resistant dual-purpose OPVs and hybrid parents; 29: Low P-tolerant varieties and integrated crop management; 30: Striga-resistant varieties and integrated crop management; 31: Waterlogging-tolerant varieties and management practices; 32: OPVs with host plant resistance to Striga hermonthica; 33: Drought-tolerant varieties; 34: Low P-tolerant/efficient variety; 35: Disease-resistant varieties and integrated crop management; 36: Weed management; 37: Ascochyta blight-resistant varieties; 38: Rust-resistant varieties
