# Supplementary material for: Multi-dimensional impact assessment for priority setting of agricultural technologies: An application of TOPSIS for the drylands of sub-Saharan Africa and South Asia
Source: PLoS One. 2024 Nov 21;19(11):e0314007. doi: 10.1371/journal.pone.0314007 (PMC11581267; doi:10.1371/journal.pone.0314007)
Supplement: S12 Table — Tech: 1: Early-maturing varieties and hybrids with tolerance to drought; 2: Varieties and hybrids with resistance to Striga; 3: Medium- to late-maturing anthracnose-resistant cultivars; 4: Soil fertility management for P and other nutrients (N, Ca) including chemical/organic fertilizers application; 5: Integrated crop management options for soil fertility, water management, Striga, intercropping; 6: Photo- and thermo-insensitive varieties; 7: Cleisto varieties and maintenance breeding to reduce varietal degeneration due to outcrossing; 8: Varieties resistant to Fusarium wilt and Cercospora leaf spot; 9: Drought-tolerant varieties; 10: Weed control; 11: Varieties tolerant to warm temperatures; 12: Intercropping-compatible varieties and integrated crop management options; 13: Pre and postharvest aflatoxin management practices including Good Agricultural Practices (GAP); 14: Disease-resistant varieties and integrated pest management and crop management practices; 15: Drought-tolerant/resistant variety and short-duration (early- maturing) variety; 16: Drought-tolerant varieties and integrated crop management; 17: Lines resistant to insects (aphid, thrips, pod sucking bug, maruca) and integrated pest management including biological control; 18: Varieties tolerant to pod borers, pod fly, pod bugs and integrated pest management; 19: Alectra-resistant varieties and integrated crop management; 20: Low P-tolerant varieties and integrated crop management; 21: Rosette-resistant variety; 22: Moderately-resistant (for short-duration variety) and highly- resistant variety (for medium- and long-duration varieties) to early and late leaf spot; 23: Disease-resistant varieties and integrated crop management; 24: Integrated soil fertility management. (DOCX) [file pone.0314007.s012.docx]

S12 Table: Estimated closeness index and ranking of technologies in dry sub-humid eastern Africa

| Crops | Tech |  | Matrix aij: criteria values | | |  | Normalized decision matrix Rij | | |  | Normalized decision matrix Vij | | |  | Si+ | Si- | Ci |  | Rank | | | | |
| --- | --- | --- | --- | --- | --- | --- | --- | --- | --- | --- | --- | --- | --- | --- | --- | --- | --- | --- | --- | --- | --- | --- | --- |
|  |  |  | BCR | Pov | Maln |  | BCR | Pov | Maln |  | BCR | Pov | Maln |  |  |  |  |  | Ci | BCR | Pov | Maln |  |
| Sorghum | 1 |  | 25 | 195222 | -6201 |  | 0.5091 | 0.7134 | -0.0004 |  | 0.2173 | 0.1395 | -0.0002 |  | 0.0000 | 0.4457 | 1.0000 |  | 1 | 1 | 1 | 1 |  |
| Sorghum | 2 |  | 12 | 114819 | -3657 |  | 0.2454 | 0.4196 | -0.0003 |  | 0.1047 | 0.0820 | -0.0001 |  | 0.1264 | 0.3943 | 0.7573 |  | 2 | 3 | 2 | 2 |  |
| Sorghum | 3 |  | 12 | 114819 | -3657 |  | 0.2426 | 0.4196 | -0.0003 |  | 0.1036 | 0.0820 | -0.0001 |  | 0.1274 | 0.3940 | 0.7556 |  | 3 | 4 | 2 | 2 |  |
| Groundnut | 4 |  | 15 | 32145 | 190 |  | 0.3043 | 0.1175 | 0.0000 |  | 0.1299 | 0.0230 | 0.0000 |  | 0.1457 | 0.3925 | 0.7293 |  | 4 | 2 | 5 | 20 |  |
| Sorghum | 5 |  | 9 | 75659 | -2348 |  | 0.1738 | 0.2765 | -0.0002 |  | 0.0742 | 0.0541 | -0.0001 |  | 0.1667 | 0.3844 | 0.6976 |  | 5 | 12 | 4 | 4 |  |
| Pigeon pea | 6 |  | 12 | 16571 | 143 |  | 0.2415 | 0.0606 | 0.0000 |  | 0.1031 | 0.0118 | 0.0000 |  | 0.1713 | 0.3858 | 0.6925 |  | 6 | 5 | 10 | 13 |  |
| Pigeon pea | 7 |  | 12 | 14693 | 128 |  | 0.2325 | 0.0537 | 0.0000 |  | 0.0992 | 0.0105 | 0.0000 |  | 0.1749 | 0.3850 | 0.6876 |  | 7 | 6 | 13 | 11 |  |
| Pigeon pea | 8 |  | 11 | 12825 | 113 |  | 0.2219 | 0.0469 | 0.0000 |  | 0.0947 | 0.0092 | 0.0000 |  | 0.1789 | 0.3841 | 0.6822 |  | 8 | 7 | 14 | 9 |  |
| Pigeon pea | 9 |  | 11 | 12825 | 113 |  | 0.2139 | 0.0469 | 0.0000 |  | 0.0913 | 0.0092 | 0.0000 |  | 0.1813 | 0.3835 | 0.6790 |  | 9 | 8 | 14 | 9 |  |
| Pigeon pea | 10 |  | 10 | 14693 | 131 |  | 0.1920 | 0.0537 | 0.0000 |  | 0.0819 | 0.0105 | 0.0000 |  | 0.1870 | 0.3820 | 0.6714 |  | 10 | 10 | 12 | 12 |  |
| Pigeon pea | 11 |  | 10 | 10965 | 98 |  | 0.1971 | 0.0401 | 0.0000 |  | 0.0841 | 0.0078 | 0.0000 |  | 0.1873 | 0.3823 | 0.6712 |  | 11 | 9 | 17 | 8 |  |
| Pigeon pea | 12 |  | 9 | 16571 | 143 |  | 0.1861 | 0.0606 | 0.0000 |  | 0.0794 | 0.0118 | 0.0000 |  | 0.1879 | 0.3817 | 0.6701 |  | 12 | 11 | 10 | 13 |  |
| Groundnut | 13 |  | 8 | 16859 | 81 |  | 0.1558 | 0.0616 | 0.0000 |  | 0.0665 | 0.0120 | 0.0000 |  | 0.1974 | 0.3801 | 0.6581 |  | 13 | 14 | 9 | 7 |  |
| Soybean | 14 |  | 8 | 5243 | -228 |  | 0.1693 | 0.0192 | 0.0000 |  | 0.0723 | 0.0037 | 0.0000 |  | 0.1987 | 0.3806 | 0.6570 |  | 14 | 13 | 23 | 5 |  |
| Groundnut | 15 |  | 7 | 25770 | 195 |  | 0.1404 | 0.0942 | 0.0000 |  | 0.0599 | 0.0184 | 0.0000 |  | 0.1986 | 0.3796 | 0.6566 |  | 15 | 17 | 6 | 21 |  |
| Cowpea | 16 |  | 7 | 17709 | 238 |  | 0.1472 | 0.0647 | 0.0000 |  | 0.0628 | 0.0127 | 0.0000 |  | 0.1999 | 0.3797 | 0.6551 |  | 16 | 15 | 8 | 22 |  |
| Cowpea | 17 |  | 7 | 19232 | 554 |  | 0.1395 | 0.0703 | 0.0000 |  | 0.0595 | 0.0137 | 0.0000 |  | 0.2018 | 0.3794 | 0.6528 |  | 17 | 18 | 7 | 23 |  |
| Pigeon pea | 18 |  | 7 | 9115 | 79 |  | 0.1365 | 0.0333 | 0.0000 |  | 0.0583 | 0.0065 | 0.0000 |  | 0.2073 | 0.3791 | 0.6465 |  | 18 | 19 | 19 | 6 |  |
| Cowpea | 19 |  | 5 | 8348 | 159 |  | 0.0908 | 0.0305 | 0.0000 |  | 0.0388 | 0.0060 | 0.0000 |  | 0.2229 | 0.3779 | 0.6290 |  | 19 | 20 | 21 | 16 |  |
| Cowpea | 20 |  | 4 | 8926 | 149 |  | 0.0825 | 0.0326 | 0.0000 |  | 0.0352 | 0.0064 | 0.0000 |  | 0.2255 | 0.3778 | 0.6262 |  | 20 | 21 | 20 | 15 |  |
| Groundnut | 21 |  | 4 | 11362 | 179 |  | 0.0795 | 0.0415 | 0.0000 |  | 0.0339 | 0.0081 | 0.0000 |  | 0.2256 | 0.3778 | 0.6262 |  | 21 | 22 | 16 | 19 |  |
| Groundnut | 22 |  | 3 | 10752 | 168 |  | 0.0586 | 0.0393 | 0.0000 |  | 0.0250 | 0.0077 | 0.0000 |  | 0.2331 | 0.3777 | 0.6183 |  | 22 | 24 | 18 | 18 |  |
| Cowpea | 23 |  | 3 | 8348 | 159 |  | 0.0607 | 0.0305 | 0.0000 |  | 0.0259 | 0.0060 | 0.0000 |  | 0.2334 | 0.3777 | 0.6181 |  | 23 | 23 | 21 | 16 |  |
| Soybean | 24 |  | 7 | 2802 | 13859215 |  | 0.1426 | 0.0102 | 1.0000 |  | 0.0609 | 0.0020 | 0.3777 |  | 0.4314 | 0.0358 | 0.0767 |  | 24 | 16 | 24 | 24 |  |
| Estimated weights: | | | 0.4268 | 0.1955 | 0.3777 |  |  |  |  |  |  |  |  |  |  |  |  |  |  |  |  |  |  |
| Positive-ideal solution: | | | |  |  |  |  |  |  |  | 0.2173 | 0.1395 | -0.0002 |  |  |  |  |  |  |  |  |  |  |
| Negative-ideal solution: | | | | |  |  |  |  |  |  | 0.0250 | 0.0020 | 0.3777 |  |  |  |  |  |  |  |  |  |  |

Tech:

1: Early-maturing varieties and hybrids with tolerance to drought; 2: Varieties and hybrids with resistance to Striga; 3: Medium- to late-maturing anthracnose-resistant cultivars; 4: Soil fertility management for P and other nutrients (N, Ca) including chemical/organic fertilizers application; 5: Integrated crop management options for soil fertility, water management, Striga, intercropping; 6: Photo- and thermo-insensitive varieties; 7: Cleisto varieties and maintenance breeding to reduce varietal degeneration due to outcrossing; 8: Varieties resistant to Fusarium wilt and Cercospora leaf spot; 9: Drought-tolerant varieties; 10: Weed control; 11: Varieties tolerant to warm temperatures; 12: Intercropping-compatible varieties and integrated crop management options; 13: Pre and postharvest aflatoxin management practices including Good Agricultural Practices (GAP); 14: Disease-resistant varieties and integrated pest management and crop management practices; 15: Drought-tolerant/resistant variety and short-duration (early- maturing) variety; 16: Drought-tolerant varieties and integrated crop management; 17: Lines resistant to insects (aphid, thrips, pod sucking bug, maruca) and integrated pest management including biological control; 18: Varieties tolerant to pod borers, pod fly, pod bugs and integrated pest management; 19: Alectra-resistant varieties and integrated crop management; 20: Low P-tolerant varieties and integrated crop management; 21: Rosette-resistant variety; 22: Moderately-resistant (for short-duration variety) and highly- resistant variety (for medium- and long-duration varieties) to early and late leaf spot; 23: Disease-resistant varieties and integrated crop management; 24: Integrated soil fertility management
