# Supplementary material for: Multi-dimensional impact assessment for priority setting of agricultural technologies: An application of TOPSIS for the drylands of sub-Saharan Africa and South Asia
Source: PLoS One. 2024 Nov 21;19(11):e0314007. doi: 10.1371/journal.pone.0314007 (PMC11581267; doi:10.1371/journal.pone.0314007)
Supplement: S13 Table — Tech: 1: Varieties resistant to Fusarium wilt and Cercospora leaf spot; 2: Intercropping-compatible varieties and integrated crop management options; 3: Cleisto varieties and maintenance breeding to reduce varietal degeneration due to outcrossing; 4: Drought-tolerant varieties; 5: Varieties tolerant to pod borers, pod fly, pod bugs and integrated pest management; 6: Photo- and thermo-insensitive varieties; 7: Low P-tolerant varieties and integrated crop management; 8: Disease-resistant varieties and integrated crop management; 9: Alectra-resistant varieties and integrated crop management; 10: Drought-tolerant varieties and integrated crop management; 11: Lines resistant to insects (aphid, thrips, pod sucking bug, maruca) and integrated pest management including biological control; 12: Integrated crop management options for soil fertility, water management, Striga, intercropping; 13: Varieties and hybrids with resistance to Striga; 14: Medium- to late-maturing anthracnose-resistant cultivars; 15: Early-maturing varieties and hybrids with tolerance to drought. (DOCX) [file pone.0314007.s013.docx]

S13 Table: Estimated closeness index and ranking of technologies in semi-arid southern Africa

| Crops | Tech |  | Matrix aij: criteria values | | |  | Normalized decision matrix Rij | | |  | Normalized decision matrix Vij | | |  | Si+ | Si- | Ci |  | Rank | | | |
| --- | --- | --- | --- | --- | --- | --- | --- | --- | --- | --- | --- | --- | --- | --- | --- | --- | --- | --- | --- | --- | --- | --- |
|  |  |  | BCR | Pov | Maln |  | BCR | Pov | Maln |  | BCR | Pov | Maln |  |  |  |  |  | Ci | BCR | Pov | Maln |
| Pigeon pea | 1 |  | 8 | 52505 | -27 |  | 0.3968 | 0.5985 | -0.1906 |  | 0.0507 | 0.1724 | -0.1113 |  | 0.0000 | 0.5085 | 1.0000 |  | 1 | 1 | 1 | 1 |
| Pigeon pea | 2 |  | 6 | 37870 | -22 |  | 0.3199 | 0.4317 | -0.1558 |  | 0.0409 | 0.1244 | -0.0910 |  | 0.0531 | 0.4757 | 0.8996 |  | 2 | 4 | 2 | 2 |
| Pigeon pea | 3 |  | 6 | 33579 | -19 |  | 0.3028 | 0.3828 | -0.1365 |  | 0.0387 | 0.1103 | -0.0797 |  | 0.0707 | 0.4617 | 0.8671 |  | 3 | 6 | 3 | 3 |
| Pigeon pea | 4 |  | 7 | 29309 | -17 |  | 0.3474 | 0.3341 | -0.1182 |  | 0.0444 | 0.0962 | -0.0691 |  | 0.0873 | 0.4491 | 0.8372 |  | 4 | 2 | 4 | 4 |
| Pigeon pea | 5 |  | 2 | 12834 | -9 |  | 0.1232 | 0.1463 | -0.0657 |  | 0.0157 | 0.0421 | -0.0384 |  | 0.1533 | 0.4114 | 0.7285 |  | 5 | 14 | 8 | 6 |
| Pigeon pea | 6 |  | 2 | 8590 | -12 |  | 0.0920 | 0.0979 | -0.0888 |  | 0.0118 | 0.0282 | -0.0519 |  | 0.1608 | 0.4243 | 0.7252 |  | 6 | 15 | 14 | 5 |
| Cowpea | 7 |  | 4 | 9721 | 10 |  | 0.2233 | 0.1108 | 0.0678 |  | 0.0285 | 0.0319 | 0.0396 |  | 0.2074 | 0.3334 | 0.6165 |  | 7 | 8 | 11 | 9 |
| Cowpea | 8 |  | 3 | 9006 | 10 |  | 0.1685 | 0.1027 | 0.0677 |  | 0.0215 | 0.0296 | 0.0396 |  | 0.2098 | 0.3331 | 0.6135 |  | 8 | 13 | 13 | 8 |
| Cowpea | 9 |  | 3 | 6288 | 9 |  | 0.1816 | 0.0717 | 0.0639 |  | 0.0232 | 0.0206 | 0.0373 |  | 0.2142 | 0.3352 | 0.6101 |  | 9 | 11 | 15 | 7 |
| Cowpea | 10 |  | 7 | 15920 | 15 |  | 0.3436 | 0.1815 | 0.1077 |  | 0.0439 | 0.0523 | 0.0629 |  | 0.2117 | 0.3128 | 0.5963 |  | 10 | 3 | 6 | 10 |
| Cowpea | 11 |  | 6 | 17137 | 20 |  | 0.3197 | 0.1953 | 0.1404 |  | 0.0408 | 0.0563 | 0.0820 |  | 0.2258 | 0.2940 | 0.5656 |  | 11 | 5 | 5 | 11 |
| Sorghum | 12 |  | 3 | 9239 | 47 |  | 0.1686 | 0.1053 | 0.3375 |  | 0.0215 | 0.0303 | 0.1972 |  | 0.3409 | 0.1758 | 0.3402 |  | 12 | 12 | 12 | 12 |
| Sorghum | 13 |  | 4 | 11152 | 57 |  | 0.2098 | 0.1271 | 0.4060 |  | 0.0268 | 0.0366 | 0.2372 |  | 0.3748 | 0.1370 | 0.2677 |  | 13 | 9 | 9 | 13 |
| Sorghum | 14 |  | 4 | 11152 | 57 |  | 0.2012 | 0.1271 | 0.4060 |  | 0.0257 | 0.0366 | 0.2372 |  | 0.3748 | 0.1369 | 0.2675 |  | 14 | 10 | 9 | 13 |
| Sorghum | 15 |  | 5 | 13572 | 90 |  | 0.2446 | 0.1547 | 0.6375 |  | 0.0313 | 0.0446 | 0.3724 |  | 0.5007 | 0.0309 | 0.0581 |  | 15 | 7 | 7 | 15 |
| Estimated weights: | | | 0.1278 | 0.2881 | 0.5842 |  |  |  |  |  |  |  |  |  |  |  |  |  |  |  |  |  |
| Positive-ideal solution: | | |  |  |  |  |  |  |  |  | 0.0507 | 0.1724 | -0.1113 |  |  |  |  |  |  |  |  |  |
| Negative-ideal solution: | | | | |  |  |  |  |  |  | 0.0118 | 0.0206 | 0.3724 |  |  |  |  |  |  |  |  |  |

Tech:

1: Varieties resistant to Fusarium wilt and Cercospora leaf spot; 2: Intercropping-compatible varieties and integrated crop management options; 3: Cleisto varieties and maintenance breeding to reduce varietal degeneration due to outcrossing; 4: Drought-tolerant varieties; 5: Varieties tolerant to pod borers, pod fly, pod bugs and integrated pest management; 6: Photo- and thermo-insensitive varieties; 7: Low P-tolerant varieties and integrated crop management; 8: Disease-resistant varieties and integrated crop management; 9: Alectra-resistant varieties and integrated crop management; 10: Drought-tolerant varieties and integrated crop management; 11: Lines resistant to insects (aphid, thrips, pod sucking bug, maruca) and integrated pest management including biological control; 12: Integrated crop management options for soil fertility, water management, Striga, intercropping; 13: Varieties and hybrids with resistance to Striga; 14: Medium- to late-maturing anthracnose-resistant cultivars; 15: Early-maturing varieties and hybrids with tolerance to drought
