# Supplementary material for: Multi-dimensional impact assessment for priority setting of agricultural technologies: An application of TOPSIS for the drylands of sub-Saharan Africa and South Asia
Source: PLoS One. 2024 Nov 21;19(11):e0314007. doi: 10.1371/journal.pone.0314007 (PMC11581267; doi:10.1371/journal.pone.0314007)
Supplement: S14 Table — Tech: 1: Rosette-resistant variety; 2: Drought-tolerant/resistant variety and short-duration (early- maturing) variety; 3: Early-maturing varieties and hybrids with tolerance to drought; 4: Soil fertility management for P and other nutrients (N, Ca) including chemical/organic fertilizers application; 5: Drought-tolerant varieties and crop management and water conservation practices; 6: Pre and postharvest aflatoxin management practices including Good Agricultural Practices (GAP); 7: Disease-resistant varieties and integrated pest management and crop management practices; 8: Use of good quality seed, appropriate seeding rate and row spacing; 9: Medium- to late-maturing anthracnose-resistant cultivars; 10: Integrated crop management options for soil fertility, water management, Striga, intercropping; 11: Varieties and hybrids with resistance to Striga; 12: Matching varieties that fit the growing period; 13: Lines resistant to insects (aphid, thrip, pod sucking bug, maruca) and integrated crop management; 14: Drought-tolerant varieties and integrated crop management; 15: Moderately-resistant variety (for short-duration variety) and highly-resistant variety (for medium- and long-duration varieties) to early and late leaf spot; 16: Disease-resistant varieties and integrated crop management; 17: Low P-tolerant varieties and integrated crop management; 18: Striga and Alectra-resistant varieties and integrated crop management; 19: Low P-tolerant/efficient variety; 20: Breeding for resistance to parasitic weeds and integrated weed management; 21: Use of inoculant and fertilizers, especially Phosphorus; 22: Establish optimum planting window and awareness creation. (DOCX) [file pone.0314007.s014.docx]

S14 Table: Estimated closeness index and ranking of technologies in dry sub-humid southern Africa

| Crops | Tech |  | Matrix aij: criteria values | | |  | Normalized decision matrix Rij | | |  | Normalized decision matrix Vij | | |  | Si+ | Si- | Ci |  | Rank | | | | |
| --- | --- | --- | --- | --- | --- | --- | --- | --- | --- | --- | --- | --- | --- | --- | --- | --- | --- | --- | --- | --- | --- | --- | --- |
|  |  |  | BCR | Pov | Maln |  | BCR | Pov | Maln |  | BCR | Pov | Maln |  |  |  |  |  | Ci | BCR | Pov | Maln |  |
| Groundnut | 1 |  | 13 | 200792 | 2 |  | 0.4621 | 0.6801 | 0.0000 |  | 0.0715 | 0.1997 | 0.0000 |  | 0.0001 | 0.4408 | 0.9998 |  | 1 | 1 | 1 | 7 |  |
| Sorghum | 2 |  | 8 | 121888 | 2 |  | 0.2810 | 0.4129 | 0.0000 |  | 0.0435 | 0.1212 | 0.0000 |  | 0.0833 | 0.4087 | 0.8306 |  | 2 | 4 | 2 | 10 |  |
| Groundnut | 3 |  | 10 | 67906 | 97 |  | 0.3534 | 0.2300 | 0.0002 |  | 0.0547 | 0.0675 | 0.0001 |  | 0.1332 | 0.3979 | 0.7492 |  | 3 | 2 | 3 | 20 |  |
| Soybean | 4 |  | 6 | 65675 | 3 |  | 0.2066 | 0.2225 | 0.0000 |  | 0.0320 | 0.0653 | 0.0000 |  | 0.1401 | 0.3956 | 0.7385 |  | 4 | 8 | 4 | 11 |  |
| Groundnut | 5 |  | 8 | 61193 | -52 |  | 0.2670 | 0.2073 | -0.0001 |  | 0.0413 | 0.0609 | -0.0001 |  | 0.1421 | 0.3957 | 0.7358 |  | 5 | 6 | 5 | 2 |  |
| Soybean | 6 |  | 5 | 55924 | 2 |  | 0.1731 | 0.1894 | 0.0000 |  | 0.0268 | 0.0556 | 0.0000 |  | 0.1509 | 0.3939 | 0.7231 |  | 6 | 11 | 6 | 8 |  |
| Soybean | 7 |  | 8 | 42644 | -41 |  | 0.2719 | 0.1444 | -0.0001 |  | 0.0421 | 0.0424 | -0.0001 |  | 0.1600 | 0.3936 | 0.7110 |  | 7 | 5 | 8 | 4 |  |
| Sorghum | 8 |  | 7 | 36783 | -68 |  | 0.2267 | 0.1246 | -0.0002 |  | 0.0351 | 0.0366 | -0.0001 |  | 0.1671 | 0.3926 | 0.7014 |  | 8 | 7 | 12 | 1 |  |
| Sorghum | 9 |  | 5 | 37360 | 67 |  | 0.1572 | 0.1265 | 0.0002 |  | 0.0243 | 0.0372 | 0.0001 |  | 0.1692 | 0.3918 | 0.6983 |  | 9 | 12 | 10 | 18 |  |
| Sorghum | 10 |  | 4 | 37360 | 66 |  | 0.1299 | 0.1265 | 0.0002 |  | 0.0201 | 0.0372 | 0.0001 |  | 0.1705 | 0.3916 | 0.6967 |  | 10 | 14 | 9 | 17 |  |
| Soybean | 11 |  | 3 | 37360 | 67 |  | 0.1163 | 0.1265 | 0.0002 |  | 0.0180 | 0.0372 | 0.0001 |  | 0.1711 | 0.3915 | 0.6959 |  | 11 | 15 | 10 | 18 |  |
| Cowpea | 12 |  | 5 | 33223 | -43 |  | 0.1886 | 0.1125 | -0.0001 |  | 0.0292 | 0.0330 | -0.0001 |  | 0.1719 | 0.3919 | 0.6950 |  | 12 | 10 | 14 | 3 |  |
| Cowpea | 13 |  | 4 | 33138 | 38 |  | 0.1347 | 0.1122 | 0.0001 |  | 0.0208 | 0.0330 | 0.0001 |  | 0.1743 | 0.3913 | 0.6919 |  | 13 | 13 | 15 | 16 |  |
| Groundnut | 14 |  | 3 | 30766 | 33 |  | 0.1142 | 0.1042 | 0.0001 |  | 0.0177 | 0.0306 | 0.0000 |  | 0.1775 | 0.3911 | 0.6879 |  | 14 | 16 | 17 | 15 |  |
| Cowpea | 15 |  | 2 | 31314 | 2 |  | 0.0723 | 0.1061 | 0.0000 |  | 0.0112 | 0.0311 | 0.0000 |  | 0.1790 | 0.3910 | 0.6859 |  | 15 | 20 | 16 | 9 |  |
| Cowpea | 16 |  | 2 | 19491 | 23 |  | 0.0794 | 0.0660 | 0.0001 |  | 0.0123 | 0.0194 | 0.0000 |  | 0.1898 | 0.3904 | 0.6729 |  | 16 | 19 | 18 | 14 |  |
| Cowpea | 17 |  | 2 | 16821 | 21 |  | 0.0862 | 0.0570 | 0.0001 |  | 0.0133 | 0.0167 | 0.0000 |  | 0.1920 | 0.3903 | 0.6703 |  | 17 | 17 | 19 | 13 |  |
| Groundnut | 18 |  | 2 | 12092 | 19 |  | 0.0842 | 0.0410 | 0.0000 |  | 0.0130 | 0.0120 | 0.0000 |  | 0.1966 | 0.3902 | 0.6650 |  | 18 | 18 | 21 | 12 |  |
| Soybean | 19 |  | 1 | 13889 | 1 |  | 0.0349 | 0.0470 | 0.0000 |  | 0.0054 | 0.0138 | 0.0000 |  | 0.1973 | 0.3902 | 0.6642 |  | 19 | 21 | 20 | 6 |  |
| Soybean | 20 |  | 1 | 5456 | -21 |  | 0.0349 | 0.0185 | -0.0001 |  | 0.0054 | 0.0054 | 0.0000 |  | 0.2052 | 0.3901 | 0.6553 |  | 20 | 22 | 22 | 5 |  |
| Soybean | 21 |  | 10 | 49075 | 279778 |  | 0.3503 | 0.1662 | 0.7071 |  | 0.0542 | 0.0488 | 0.3901 |  | 0.4187 | 0.0653 | 0.1349 |  | 21 | 3 | 7 | 21 |  |
| Groundnut | 22 |  | 6 | 35465 | 279778 |  | 0.2008 | 0.1201 | 0.7071 |  | 0.0311 | 0.0353 | 0.3901 |  | 0.4254 | 0.0394 | 0.0847 |  | 22 | 9 | 13 | 21 |  |
| Estimated weights: | | | 0.1547 | 0.2936 | 0.5517 |  |  |  |  |  |  |  |  |  |  |  |  |  |  |  |  |  |  |
| Positive-ideal solution: | | | |  |  |  |  |  |  |  | 0.0715 | 0.1997 | -0.0001 |  |  |  |  |  |  |  |  |  |  |
| Negative-ideal solution: | | | | |  |  |  |  |  |  | 0.0054 | 0.0054 | 0.3901 |  |  |  |  |  |  |  |  |  |  |

Tech: 1: Rosette-resistant variety; 2: Drought-tolerant/resistant variety and short-duration (early- maturing) variety; 3: Early-maturing varieties and hybrids with tolerance to drought; 4: Soil fertility management for P and other nutrients (N, Ca) including chemical/organic fertilizers application; 5: Drought-tolerant varieties and crop management and water conservation practices; 6: Pre and postharvest aflatoxin management practices including Good Agricultural Practices (GAP); 7: Disease-resistant varieties and integrated pest management and crop management practices; 8: Use of good quality seed, appropriate seeding rate and row spacing; 9: Medium- to late-maturing anthracnose-resistant cultivars; 10: Integrated crop management options for soil fertility, water management, Striga, intercropping; 11: Varieties and hybrids with resistance to Striga; 12: Matching varieties that fit the growing period; 13: Lines resistant to insects (aphid, thrip, pod sucking bug, maruca) and integrated crop management; 14: Drought-tolerant varieties and integrated crop management; 15: Moderately-resistant variety (for short-duration variety) and highly-resistant variety (for medium- and long-duration varieties) to early and late leaf spot; 16: Disease-resistant varieties and integrated crop management; 17: Low P-tolerant varieties and integrated crop management; 18: Striga and Alectra-resistant varieties and integrated crop management; 19: Low P-tolerant/efficient variety; 20: Breeding for resistance to parasitic weeds and integrated weed management; 21: Use of inoculant and fertilizers, especially Phosphorus; 22: Establish optimum planting window and awareness creation
