# Supplementary material for: Multi-dimensional impact assessment for priority setting of agricultural technologies: An application of TOPSIS for the drylands of sub-Saharan Africa and South Asia
Source: PLoS One. 2024 Nov 21;19(11):e0314007. doi: 10.1371/journal.pone.0314007 (PMC11581267; doi:10.1371/journal.pone.0314007)
Supplement: S15 Table — Tech: 1: Varieties resistant to Fusarium wilt and root rots; 2: Drought-tolerant varieties; 3: Herbicide-tolerant varieties to control weeds; 4: Varieties resistant to Fusarium wilt and Cercospora leaf spot; 5: Genetically diverse dual-purpose hybrid parents/cultivars with high and stable yields with disease resistance (downy mildew and blast); 6: Breeding for early-maturing, drought-tolerant hybrids which can give stable yields under severe drought conditions; 7: Heat-tolerant varieties; 8 Pod borer-tolerant varieties and integrated pest management; 9: Soil fertility management for P and other nutrients (N, Ca) including chemical/organic fertilizers application; 10: Integrated crop management; 11: Sterility mosaic disease-resistant varieties; 12: Intercropping compatible-varieties and integrated crop management options; 13: Drought-tolerant varieties; 14: Integrated crop management; 15: Varieties tolerant to pod borers, pod fly, pod bugs and integrated pest management; 16: Genetic base diversification; 17: Pre and postharvest aflatoxin management practices including Good Agricultural Practices (GAP); 18: Varieties resistant to wilt and root rots and integrated pest management; 19: Cultivars tolerant to head bugs and grain mold; 20: Shoot fly-resistant cultivars; 21: Charcoal rot-resistant cultivars; 22: Early-maturing varieties and hybrids with tolerance to drought; 23: Drought-tolerant varieties; 24: Varieties resistant to diseases (foliar fungal, bud necrosis, soil borne); 25: Genetically diverse dual-purpose hybrid parents/cultivars with high and stable yields; 26: Integrated crop management practices; 27: Breeding for early-maturing, drought-tolerant OPVs and hybrids which can give stable yields under severe drought conditions; 28: Breeding for downy mildew- and smut-resistant dual-purpose OPVs and hybrid parents; 29: Herbicide-tolerant varieties to control weeds; 30: Heat-tolerant varieties; 31: Low P-tolerant/efficient variety. (DOCX) [file pone.0314007.s015.docx]

S15 Table: Estimated closeness index and ranking of technologies in semi-arid south Asia

| Crops | Tech |  | | Matrix aij: criteria values | | |  | Normalized decision matrix Rij | | |  | Normalized decision matrix Vij | | |  | Si+ | Si- | Ci |  | Rank | | | | |
| --- | --- | --- | --- | --- | --- | --- | --- | --- | --- | --- | --- | --- | --- | --- | --- | --- | --- | --- | --- | --- | --- | --- | --- | --- |
|  |  |  | | BCR | Pov | Maln |  | BCR | Pov | Maln |  | BCR | Pov | Maln |  |  |  |  |  | Ci | BCR | Pov | Maln |  |
| Chickpea | 1 |  | | 17 | 145277 | -14864 |  | 0.3708 | 0.5039 | -0.5029 |  | 0.0957 | 0.1801 | -0.1934 |  | 0.0000 | 0.2773 | 1.0000 |  | 1 | 1 | 1 | 1 |  |
| Chickpea | 2 |  | | 11 | 100898 | -11652 |  | 0.2354 | 0.3500 | -0.3942 |  | 0.0607 | 0.1251 | -0.1516 |  | 0.0774 | 0.2024 | 0.7233 |  | 2 | 7 | 3 | 2 |  |
| Chickpea | 3 |  | | 12 | 67265 | -11652 |  | 0.2577 | 0.2333 | -0.3942 |  | 0.0665 | 0.0834 | -0.1516 |  | 0.1093 | 0.1818 | 0.6246 |  | 3 | 6 | 5 | 2 |  |
| Pigeonpea | 4 |  | | 15 | 53702 | -10065 |  | 0.3358 | 0.1863 | -0.3405 |  | 0.0866 | 0.0666 | -0.1310 |  | 0.1299 | 0.1659 | 0.5609 |  | 4 | 3 | 8 | 4 |  |
| Pearl millet | 5 |  | | 10 | 125451 | -4312 |  | 0.2261 | 0.4352 | -0.1459 |  | 0.0583 | 0.1555 | -0.0561 |  | 0.1444 | 0.1713 | 0.5425 |  | 5 | 8 | 2 | 10 |  |
| Pearl millet | 6 |  | | 7 | 77973 | -2654 |  | 0.1476 | 0.2705 | -0.0898 |  | 0.0381 | 0.0966 | -0.0345 |  | 0.1885 | 0.1054 | 0.3586 |  | 6 | 12 | 4 | 14 |  |
| Chickpea | 7 |  | | 2 | 18581 | -7557 |  | 0.0475 | 0.0645 | -0.2557 |  | 0.0123 | 0.0230 | -0.0983 |  | 0.2016 | 0.1007 | 0.3331 |  | 7 | 29 | 17 | 5 |  |
| Chickpea | 8 |  | | 4 | 30228 | -6241 |  | 0.0881 | 0.1049 | -0.2111 |  | 0.0227 | 0.0375 | -0.0812 |  | 0.1956 | 0.0899 | 0.3150 |  | 8 | 20 | 15 | 6 |  |
| Groundnut | 9 |  | | 16 | 44384 | -504 |  | 0.3584 | 0.1540 | -0.0171 |  | 0.0925 | 0.0550 | -0.0066 |  | 0.2249 | 0.0999 | 0.3076 |  | 9 | 2 | 12 | 29 |  |
| Pearl millet | 10 |  | | 6 | 58409 | -2243 |  | 0.1318 | 0.2026 | -0.0759 |  | 0.0340 | 0.0724 | -0.0292 |  | 0.2058 | 0.0807 | 0.2818 |  | 10 | 14 | 6 | 16 |  |
| Pigeonpea | 11 |  | | 4 | 12181 | -6156 |  | 0.0825 | 0.0423 | -0.2083 |  | 0.0213 | 0.0151 | -0.0801 |  | 0.2135 | 0.0823 | 0.2781 |  | 11 | 22 | 22 | 7 |  |
| Pigeonpea | 12 |  | | 2 | 12181 | -6156 |  | 0.0525 | 0.0423 | -0.2083 |  | 0.0136 | 0.0151 | -0.0801 |  | 0.2163 | 0.0814 | 0.2734 |  | 12 | 28 | 23 | 7 |  |
| Lentil | 13 |  | | 14 | 15112 | -2477 |  | 0.3197 | 0.0524 | -0.0838 |  | 0.0825 | 0.0187 | -0.0322 |  | 0.2285 | 0.0827 | 0.2658 |  | 13 | 4 | 21 | 15 |  |
| Finger millet | 14 |  | | 6 | 51219 | -1674 |  | 0.1240 | 0.1777 | -0.0566 |  | 0.0320 | 0.0635 | -0.0218 |  | 0.2171 | 0.0697 | 0.2431 |  | 14 | 15 | 9 | 20 |  |
| Pigeonpea | 15 |  | | 5 | 18199 | -4716 |  | 0.1120 | 0.0631 | -0.1595 |  | 0.0289 | 0.0226 | -0.0614 |  | 0.2161 | 0.0680 | 0.2394 |  | 15 | 18 | 19 | 9 |  |
| Sorghum | 16 |  | | 4 | 56170 | -851 |  | 0.0863 | 0.1948 | -0.0288 |  | 0.0223 | 0.0696 | -0.0111 |  | 0.2255 | 0.0703 | 0.2376 |  | 16 | 21 | 7 | 23 |  |
| Groundnut | 17 |  | | 9 | 32354 | -1820 |  | 0.2121 | 0.1122 | -0.0616 |  | 0.0547 | 0.0401 | -0.0237 |  | 0.2238 | 0.0648 | 0.2245 |  | 17 | 9 | 14 | 19 |  |
| Lentil | 18 |  | | 12 | 11567 | -2038 |  | 0.2649 | 0.0401 | -0.0689 |  | 0.0683 | 0.0143 | -0.0265 |  | 0.2368 | 0.0669 | 0.2202 |  | 18 | 5 | 24 | 17 |  |
| Sorghum | 19 |  | | 4 | 49832 | -727 |  | 0.0813 | 0.1729 | -0.0246 |  | 0.0210 | 0.0618 | -0.0095 |  | 0.2311 | 0.0622 | 0.2120 |  | 19 | 23 | 10 | 27 |  |
| Sorghum | 20 |  | | 3 | 49831 | -739 |  | 0.0764 | 0.1729 | -0.0250 |  | 0.0197 | 0.0618 | -0.0096 |  | 0.2314 | 0.0620 | 0.2112 |  | 20 | 25 | 11 | 26 |  |
| Sorghum | 21 |  | | 3 | 43514 | -607 |  | 0.0592 | 0.1509 | -0.0205 |  | 0.0153 | 0.0539 | -0.0079 |  | 0.2383 | 0.0534 | 0.1829 |  | 21 | 27 | 13 | 28 |  |
| Sorghum | 22 |  | | 5 | 18200 | -2939 |  | 0.1165 | 0.0631 | -0.0994 |  | 0.0301 | 0.0226 | -0.0382 |  | 0.2306 | 0.0487 | 0.1744 |  | 22 | 17 | 18 | 12 |  |
| Pigeonpea | 23 |  | | 2 | 6751 | -3466 |  | 0.0423 | 0.0234 | -0.1173 |  | 0.0109 | 0.0084 | -0.0451 |  | 0.2422 | 0.0457 | 0.1587 |  | 23 | 30 | 27 | 11 |  |
| Groundnut | 24 |  | | 7 | 21187 | -1148 |  | 0.1671 | 0.0735 | -0.0388 |  | 0.0431 | 0.0263 | -0.0149 |  | 0.2414 | 0.0452 | 0.1577 |  | 24 | 10 | 16 | 21 |  |
| Finger millet | 25 |  | | 4 | 8018 | -2791 |  | 0.0924 | 0.0278 | -0.0944 |  | 0.0238 | 0.0099 | -0.0363 |  | 0.2425 | 0.0404 | 0.1427 |  | 25 | 19 | 26 | 13 |  |
| Groundnut | 26 |  | | 7 | 15877 | -1129 |  | 0.1541 | 0.0551 | -0.0382 |  | 0.0398 | 0.0197 | -0.0147 |  | 0.2466 | 0.0391 | 0.1370 |  | 26 | 11 | 20 | 22 |  |
| Finger millet | 27 |  | | 4 | 5716 | -1977 |  | 0.0802 | 0.0198 | -0.0669 |  | 0.0207 | 0.0071 | -0.0257 |  | 0.2523 | 0.0291 | 0.1033 |  | 27 | 24 | 28 | 18 |  |
| Finger millet | 28 |  | | 6 | 9172 | 0 |  | 0.1385 | 0.0318 | 0.0000 |  | 0.0357 | 0.0114 | 0.0000 |  | 0.2636 | 0.0291 | 0.0996 |  | 28 | 13 | 25 | 31 |  |
| Lentil | 29 |  | | 6 | 3510 | -795 |  | 0.1232 | 0.0122 | -0.0269 |  | 0.0318 | 0.0044 | -0.0103 |  | 0.2617 | 0.0258 | 0.0898 |  | 29 | 16 | 29 | 24 |  |
| Lentil | 30 |  | | 3 | 2079 | -762 |  | 0.0600 | 0.0072 | -0.0258 |  | 0.0155 | 0.0026 | -0.0099 |  | 0.2676 | 0.0123 | 0.0439 |  | 30 | 26 | 30 | 25 |  |
| Groundnut | 31 |  | | 1 | 1320 | -421 |  | 0.0321 | 0.0046 | -0.0142 |  | 0.0083 | 0.0016 | -0.0055 |  | 0.2735 | 0.0055 | 0.0196 |  | 31 | 31 | 31 | 30 |  |
| Estimated weights: | | | 0.2580 | | 0.3573 | 0.3847 |  |  |  |  |  |  |  |  |  |  |  |  |  |  |  |  |  |  |
| Positive-ideal solution: | | |  | |  |  |  |  |  |  |  | 0.0957 | 0.1801 | -0.1935 |  |  |  |  |  |  |  |  |  |  |
| Negative-ideal solution: | | | | | |  |  |  |  |  |  | 0.0083 | 0.0016 | 0.0000 |  |  |  |  |  |  |  |  |  |  |

Tech:

1: Varieties resistant to Fusarium wilt and root rots; 2: Drought-tolerant varieties; 3: Herbicide-tolerant varieties to control weeds; 4: Varieties resistant to Fusarium wilt and Cercospora leaf spot; 5: Genetically diverse dual-purpose hybrid parents/cultivars with high and stable yields with disease resistance (downy mildew and blast); 6: Breeding for early-maturing, drought-tolerant hybrids which can give stable yields under severe drought conditions; 7: Heat-tolerant varieties; 8 Pod borer-tolerant varieties and integrated pest management; 9: Soil fertility management for P and other nutrients (N, Ca) including chemical/organic fertilizers application; 10: Integrated crop management; 11: Sterility mosaic disease-resistant varieties; 12: Intercropping compatible-varieties and integrated crop management options; 13: Drought-tolerant varieties; 14: Integrated crop management; 15: Varieties tolerant to pod borers, pod fly, pod bugs and integrated pest management; 16: Genetic base diversification; 17: Pre and postharvest aflatoxin management practices including Good Agricultural Practices (GAP); 18: Varieties resistant to wilt and root rots and integrated pest management; 19: Cultivars tolerant to head bugs and grain mold; 20: Shoot fly-resistant cultivars; 21: Charcoal rot-resistant cultivars; 22: Early-maturing varieties and hybrids with tolerance to drought; 23: Drought-tolerant varieties; 24: Varieties resistant to diseases (foliar fungal, bud necrosis, soil borne); 25: Genetically diverse dual-purpose hybrid parents/cultivars with high and stable yields; 26: Integrated crop management practices; 27: Breeding for early-maturing, drought-tolerant OPVs and hybrids which can give stable yields under severe drought conditions; 28: Breeding for downy mildew- and smut-resistant dual-purpose OPVs and hybrid parents; 29: Herbicide-tolerant varieties to control weeds; 30: Heat-tolerant varieties; 31: Low P-tolerant/efficient variety
