# Supplementary material for: Multi-dimensional impact assessment for priority setting of agricultural technologies: An application of TOPSIS for the drylands of sub-Saharan Africa and South Asia
Source: PLoS One. 2024 Nov 21;19(11):e0314007. doi: 10.1371/journal.pone.0314007 (PMC11581267; doi:10.1371/journal.pone.0314007)
Supplement: S16 Table — Tech: 1: Varieties resistant to Fusarium wilt and root rots; 2: Botrytis gray mold-resistant varieties; 3: Pod borer-tolerant varieties and integrated pest management; 4: Drought-tolerant varieties; 5 Herbicide-tolerant varieties to control weeds; 6: Drought-tolerant varieties; 7: Stemphylium blight-resistant varieties and integrated pest management; 8: Heat-tolerant varieties. (DOCX) [file pone.0314007.s016.docx]

S16 Table: Estimated closeness index and ranking of technologies in dry sub-humid South Asia

| Crops | Tech |  | Matrix aij: criteria values | | |  | Normalized decision matrix Rij | | |  | Normalized decision matrix Vij | | |  | Si+ | Si- | Ci |  | Rank | | | |
| --- | --- | --- | --- | --- | --- | --- | --- | --- | --- | --- | --- | --- | --- | --- | --- | --- | --- | --- | --- | --- | --- | --- |
|  |  |  | BCR | Pov | Maln |  | BCR | Pov | Maln |  | BCR | Pov | Maln |  |  |  |  |  | Ci | BCR | Pov | Maln |
| Chickpea | 1 |  | 12 | 206145 | -16919 |  | 0.6087 | 0.7540 | -0.6930 |  | 0.1487 | 0.2777 | -0.2685 |  | 0.0000 | 0.4019 | 1.0000 |  | 1 | 1 | 1 | 1 |
| Chickpea | 2 |  | 9 | 159868 | -13256 |  | 0.4722 | 0.5847 | -0.5429 |  | 0.1153 | 0.2153 | -0.2104 |  | 0.0915 | 0.3104 | 0.7723 |  | 2 | 2 | 2 | 2 |
| Chickpea | 3 |  | 5 | 75874 | -9234 |  | 0.2568 | 0.2775 | -0.3782 |  | 0.0627 | 0.1022 | -0.1466 |  | 0.2303 | 0.1775 | 0.4353 |  | 3 | 6 | 3 | 3 |
| Chickpea | 4 |  | 0 | 14447 | -6817 |  | 0.0011 | 0.0528 | -0.2792 |  | 0.0003 | 0.0195 | -0.1082 |  | 0.3382 | 0.0999 | 0.2279 |  | 4 | 8 | 6 | 4 |
| Lentil | 5 |  | 8 | 13726 | -690 |  | 0.4071 | 0.0502 | -0.0283 |  | 0.0994 | 0.0185 | -0.0110 |  | 0.3687 | 0.0996 | 0.2127 |  | 5 | 3 | 7 | 7 |
| Lentil | 6 |  | 5 | 16776 | -967 |  | 0.2829 | 0.0614 | -0.0396 |  | 0.0691 | 0.0226 | -0.0154 |  | 0.3681 | 0.0704 | 0.1606 |  | 6 | 4 | 4 | 5 |
| Lentil | 7 |  | 5 | 14662 | -836 |  | 0.2626 | 0.0536 | -0.0342 |  | 0.0641 | 0.0197 | -0.0133 |  | 0.3726 | 0.0649 | 0.1483 |  | 7 | 5 | 5 | 6 |
| Lentil | 8 |  | 3 | 6781 | -560 |  | 0.1607 | 0.0248 | -0.0229 |  | 0.0392 | 0.0091 | -0.0089 |  | 0.3892 | 0.0390 | 0.0910 |  | 8 | 7 | 8 | 8 |
| Estimated weights: | | | 0.2442 | 0.3683 | 0.3875 |  |  |  |  |  |  |  |  |  |  |  |  |  |  |  |  |  |
| Positive-ideal solution: | | |  |  |  |  |  |  |  |  | 0.1487 | 0.2777 | -0.2685 |  |  |  |  |  |  |  |  |  |
| Negative-ideal solution: | | | | |  |  |  |  |  |  | 0.0003 | 0.0091 | -0.0089 |  |  |  |  |  |  |  |  |  |

Tech:

1: Varieties resistant to Fusarium wilt and root rots; 2: Botrytis gray mold-resistant varieties; 3: Pod borer-tolerant varieties and integrated pest management; 4: Drought-tolerant varieties; 5 Herbicide-tolerant varieties to control weeds; 6: Drought-tolerant varieties; 7: Stemphylium blight-resistant varieties and integrated pest management; 8: Heat-tolerant varieties
